# The Octocoral Trait Database: a global database of trait information for octocoral species

**DOI:** 10.1038/s41597-024-04307-8

**Published:** 2025-01-15

**Authors:** D. Gómez-Gras, C. Linares, N. Viladrich, Y. Zentner, J. Grinyó, A. Gori, C. S. McFadden, K. E. Fabricius, J. S. Madin

**Affiliations:** 1 Hawai‘i Institute of Marine Biology, University of Hawai‘i at Mānoa, Kāne’ohe, Hawai‘i USA; 2 Departament Evolutionary Biology, Ecology and Environmental Sciences, Universitat de Barcelona (UB), Barcelona, Spain; 3 Institut de Recerca de la Biodiversitat (IRBio), Universitat de Barcelona (UB), Barcelona, Spain; 4 Institut de Ciències Del Mar (ICM-CSIC), Barcelona, Spain; 5 Department of Ocean System Sciences, NIOZ Royal Netherlands Institute for Sea Research and Utrecht University, Den Burg, the Netherlands; 6 Department of Biology, Harvey Mudd College, Claremont, California USA; 7 Australian Institute of Marine Science, Townsville, Queensland Australia

**Keywords:** Community ecology, Marine biology, Biodiversity

## Abstract

Trait-based approaches are revolutionizing our understanding of high-diversity ecosystems by providing insights into the principles underlying key ecological processes, such as community assembly, species distribution, resilience, and the relationship between biodiversity and ecosystem functioning. In 2016, the Coral Trait Database advanced coral reef science by centralizing trait information for stony corals (i.e., Subphylum Anthozoa, Class Hexacorallia, Order Scleractinia). However, the absence of trait data for soft corals, gorgonians, and sea pens (i.e., Class Octocorallia) limits our understanding of ecosystems where these organisms are significant members and play pivotal roles. To address this gap, we introduce the Octocoral Trait Database, a global, open-source database of curated trait data for octocorals. This database houses species- and individual-level data, complemented by contextual information that provides a relevant framework for analyses. The inaugural dataset, OctocoralTraits v2.2, contains over 97,500 global trait observations across 98 traits and over 3,500 species. The database aims to evolve into a steadily growing, community-led resource that advances future marine science, with a particular emphasis on coral reef research.

## Background and Summary

Traits - the measurable properties of organisms - capture the biology and morphology of species and have been used for centuries to infer species boundaries based on shared characteristics^1^. Traits also dictate organismal performance and its interaction with the ecological niche^2,3^. Thus, exploring patterns of trait diversity across organisms and species also brings us closer to understanding the general principles that determine, among other processes, the patterns of abundance and distribution of species^4,5^, their evolution^6^, their resilience (e.g., resistance and recovery capacity)^7,8^, or their contribution to ecosystem functioning and services^9,10^. Consequently, the use of trait-based approaches in ecology has exponentially increased in recent decades, leading both to the development of new methods and an increase in the necessity of trait data among the scientific community^11^. The compilation of trait data across the Tree of Life remains nonetheless a major challenge^12^. In fact, trait databases are still restricted to relatively few taxonomic groups of mostly terrestrial organisms (e.g.^13–20^), and are even rarer in other systems traditionally less accessible to scientists, such as the marine realm. Nevertheless, considerable effort has recently been made to compile trait data for some marine groups (e.g., stony corals^21^, algae^22^, and fish^23^, among others). However, trait data gaps still exist for most taxa, and even when data are available for some species, it is often difficult and time-consuming to integrate them within a single study^24^. This is in part because trait information mostly comes from scattered sources that are often difficult to find or access, and often including different languages (e.g., taxonomic descriptions, local identification guides, grey literature, etc.). In addition, traits are often measured using differing methodologies and standards, and relevant contextual information is often lacking. This highlights the need for new trait databases that simplify the process for neglected yet ecologically important marine taxa.

Coral-dominated ecosystems are some of the most biodiverse ecosystems on Earth^25^. They provide millions of people with innumerable services including food provision, coastal protection, or recreation^26^. Yet, coral-dominated ecosystems are being severely transformed by global change, leading them into uncharted territories^27–29^. To advance the science of these systems more rapidly at this time of accelerating change, the Coral Trait Database was launched in 2016^21^, which centralized diverse trait information for stony corals (i.e., Sub-Phylum Anthozoa, Class Hexacorallia, Order Scleractinia) into a single, open-access repository. The Coral Trait Database became the basis for research and education that has advanced coral reef science worldwide (e.g.^8,27,30,31^). However, these advances are limited because of the lack of information for the anthozoan Class Octocorallia, which significantly contributes to the biodiversity and functioning of many coral-dominated marine ecosystems.

Octocorals are a class of anthozoans that host more than 3,500 nominal species of mainly non-stony corals (e.g., soft corals, sea fans and sea pens) distributed across more than 75 families^32^. They can be found from the shallow waters to the deep sea and across all marine ecoregions^33^. Octocorals differ from hexacorals (i.e., scleractinians) in many traits; most notably they have polyps with eight pinnate tentacles rather than six or 6-fold non-pinnate tentacles, and they tend to have unconsolidated sclerites or semi-rigid skeletons rather than the hard skeletons of Scleractinia^34,35^. Like hexacorals, octocorals also provide critical functions to ecosystems and services to human societies. Octocorals can be foundation species that form three-dimensional habitats that are home, refuge, and spawning grounds for many associated taxa, which in turn increases biodiversity, stability, and food provision to humans^36–40^. Their complex morphologies can also locally modify water flows and light intensity, which may favor the settlement of sessile invertebrate larvae over opportunistic macroalgae, thus further promoting assemblage stability and resilience^41,42^. Octocorals can also directly influence nutrient cycling and the trophic network by acting as food source (e.g., for nudibranchs), and as suspension feeders that capture particulate organic matter and plankton from the water column^43–47^. Moreover, although octocorals are not typically considered reef-builders, they can contribute to the carbon cycle and reef calcification processes by photosynthesis and by fixing calcium carbonate (CaCO_3_) in their sclerites and skeletal structures^48–50^. Skeletons of some octocoral species such as *Corallium rubrum* have been used for jewelry by humans since ancient times^51^. Octocorals are also important sources of pharmaceutical products and have a high aesthetic value that enhances dive tourism and inspiration^52,53^.

Under intensifying global change, many octocoral species are playing important roles in terms of reef reconfiguration processes. For instance, in the Caribbean Sea, where stony corals have generally suffered marked declines in abundance following cumulative climatic impacts, most octocoral species have prevailed^54–56^. Similarly, some octocorals are becoming the new dominant groups in some impacted reefs of the Western and Central Indo-Pacific following a decline in hard coral cover^57,58^. Conversely, in other regions such as the Mediterranean Sea, habitat-forming octocorals are experiencing population collapses following recurrent marine heatwaves^59^.

Octocorals and their traits are thus an undeniably crucial piece of the puzzle to understand how coral-dominated ecosystems function, how they are being transformed by global change, and how we can improve their management in the Anthropocene. Here, we introduce the Octocoral Trait Database, a global, open-source database of curated trait data for octocorals. We release its first data descriptor, OctocoralTraits v2.2, which has been integrated with those data of the scleractinian corals in the Coral Trait Database (www.coraltraits.org), and hosts species- and individual-level data alongside contextual data that provide relevant framing for analyses. The primary goals of the new database are: (i) to aggregate diverse information on octocoral traits into a single open-access repository that ensures transparent and accurate archiving of coral trait data, (ii) to promote the appropriate crediting of original data sources, (iii) to continue engaging the reef coral research community in the gathering and quality control of trait data to facilitate future coral reef research, and iv) to expand the current stony coral version of the Coral Trait Database with octocoral data to promote the advancement of marine science, with a particular emphasis on coral reefs. Here, we publish a first global data release that contains a sample with more than 97,500 error-checked trait observations, including over 148,000 trait measurements across 128 traits (30 of which are contextual) and over 3500 valid octocoral species. Trait observations were compiled across all marine ecoregions and across all depth zones, from the deep sea to the shallow waters. Furthermore, while OctocoralTraits v2.2 serves as a static data descriptor, we envision it as an evolving data product that will continue to grow collaboratively to further facilitate the quantification of trait variation among coral species in the Anthropocene.

## Methods

### Ontology of the data descriptor

**Fig. 1:**
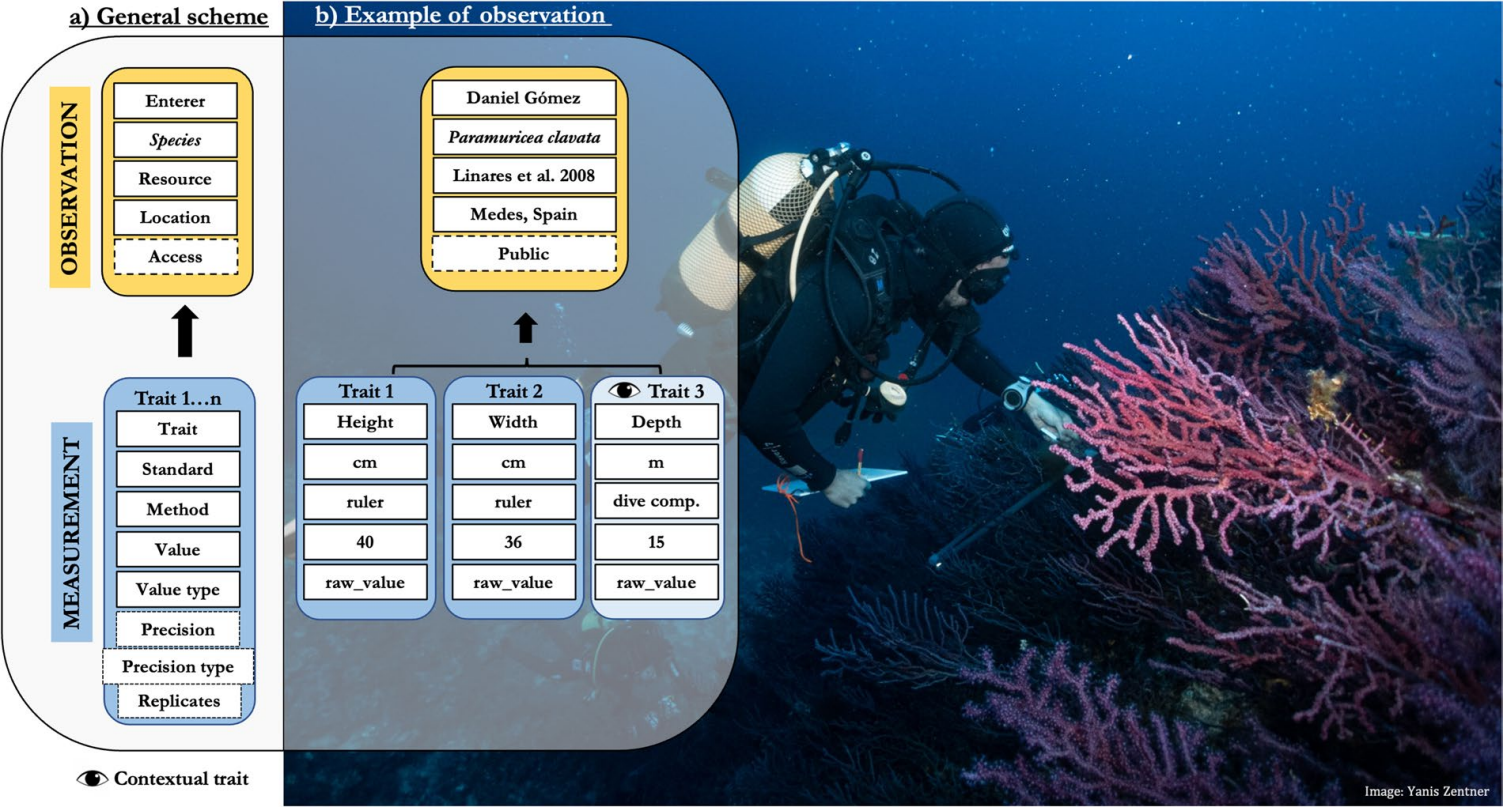
(**a**) General scheme on how data is structured in the database following the Observation-Measurement scheme^61^ and Madin *et al*.^21^. (**b**) Example of an observation. Each observation contains data for the data enterer, the species of interest, the scientific source, the type of access (i.e., optional variable that data enterers can control to keep data private before publication) and the location. In addition, each observation binds one or various measurements that apart from specifying the name of the measured trait (e.g., height of the colony), include information about: the standard used to measure it (e.g., m, cm…etc), the method used (e.g., ruler), the actual value measured, and the value type (e.g., raw data for a single measurement, expert/group opinion for single/consensus view of experts, mean, median, range, maximum, or minimum…). The number of replicates and estimates of precision (e.g., standard error, standard deviation…) can also be specified when applicable. Finally, one or multiple contextual traits (e.g., water depth, habitat type…), that might be relevant for explaining intra-specific variation in the trait/s of interest can also be associated to the observation.

The structure of OctocoralTraits v2.2 matches that of the data descriptor published by Madin *et al*.^21^ for scleractinian corals, as well as those of other similar trait databases, such as AusTraits^60^. Specifically, it follows the principles of the Observation and Measurement Ontology^61^, where observations at the individual or species level bind associated measurements and may provide context for other observations (Fig. 1). For example, recording both the height and width of the same octocoral colony would be considered a single observation with two distinct measurements, each representing a different trait of the coral. If the water depth is also noted, it remains part of the same observation but is categorized as a contextual trait, as it does not directly pertain to the colony itself.

### Traits selection

OctocoralTraits v2.2 has been compiled to populate the Class Octocorallia within the Coral Trait Database with the first global data descriptor. To achieve this goal, we first identified a set of relevant octocoral traits based on: i) traits that were already present for scleractinian corals in the Coral Traits database and that also apply to octocorals, and ii) new traits identified by the team as relevant for octocorals in terms of their biomechanical, morphological, physiological, biogeographical, ecological, conservation and/or reproductive characteristics. The selected traits include both individual-level traits (i.e., heritable features of organisms) that are measured at the individual level and can potentially vary within species (e.g., growth rate, colony height) and species-level traits that are properties of species as entities and, therefore, invariant within species (e.g., species depth range, conservation status). The complete list of 98 selected traits can be found in Table S1, along with their definitions and detailed information on their types of associated variables (e.g., numeric vs categorical) and allowed values. Table S2 includes information on the 30 contextual traits (e.g., habitat characteristics such as depth), which provide additional information about the specific conditions under which individual-level traits were quantified. This information is crucial for understanding trait variation^21^.

### Data acquisition

OctocoralTraits v2.2 was assembled using only scientific sources, ensuring peer-reviewed level of quality control. Specifically, in April 2022, we searched the Web of Science for scientific works using the general query: “octocoral*” OR “soft coral” OR “sea pen” OR “gorgonian” OR “sea fan”. We conducted a single, general search to balance the creation of an initial global dataset of octocoral trait data for the Coral Trait Database with maintaining a manageable workload for the team. This search yielded over 11,000 scientific sources, which were then manually screened at the abstract level using the *abstrackr* software^62^ to exclude sources unrelated to the traits or organisms of interest. This process led to an initial selection of 2,360 potentially relevant publications. However, many of them were subsequently excluded upon further review for not being related to the organisms or traits of interest. For example, many sources mentioned ‘soft corals’ in the abstract but referred to organisms outside of the Class Octocorallia, such as zoanthids.

From the selected sources, the data extraction process primarily involved capturing values from text and result tables. In the few cases where information was only available in figures, data were extracted by establishing a scale based on axis values using ImageJ2 software^63^. Finally, as data compilation progressed, some additional articles of interest—those not captured by the initial search but referenced within reviewed sources—were also included, along with some newly published articles unavailable at the time of the initial search. Similarly, although the initial search focused primarily on publications in English, some relevant publications in other languages (e.g., Spanish, German) were also included where team members were sufficiently confident with those languages. The complete list of 796 used data sources can be found at Supp. Table S3^32,36,49,64–859^.

This final list of data sources included published research articles, taxonomic monographs, scientific books, theses, identification guides, published conference abstracts and reports. Moreover, for the geographical traits’ marine province and marine realm, trait data compilation from the aforementioned sources was supplemented by information from the online, public, scientific platform OBIS (https://obis.org^846^). Specifically, we first retrieved occurrence data from the platform using the robis R package^860^ in RStudio software v2023.06.0 + 421^861^, utilizing the Aphia_ID code of each species as the taxon ID. Spatial outliers were then flagged with the CoordinateCleaner R package^862^ and manually examined for accuracy by consulting original sources. Verified data were subsequently classified into realms and/or provinces using the meowR package^863^ and incorporated into the data descriptor.

### Species taxonomy

For the static OctocoralTraits v2.2 used in this descriptor, all trait observations are associated with accepted species of octocorals. The accepted name and taxonomy for each species were determined based on the most authoritative and up-to-date lists of octocoral species, namely the 2024 World List of Octocorallia^864^ and the World Register of Marine Species (WORMS, as of May 2024^865^) (http://www.marinespecies.org). In cases where source publications contained trait data for combinations that are no longer valid (e.g., *Pseudopterogorgia americana*), species names were updated in the database to reflect the currently accepted name (e.g., *Antillogorgia americana*). To ensure the traceability of taxonomic updates, all trait observations also include information on the Aphia ID code for the original species that appeared in the scientific source from which trait data was obtained. These Aphia ID codes are linked to the WORMS platform.

### Data integration

To progressively integrate trait data from various scientific sources into a unified, structured dataset, we implemented a data processing workflow following Madin *et al*.^21^. Specifically, we: (i) manually assigned observation IDs to link related measurements, (ii) reformatted location coordinates to the decimal degree system when necessary, (iii) standardized terminology for traits, locations, methodologies, and standards across sources, (iv) updated taxonomy as needed (see previous section for details), and (v) harmonized values for categorical traits, as terminology can vary significantly among sources (refer to Table S1 for accepted values). A notable challenge was the extensive variety of nomenclature used in the scientific literature to describe growth forms across the Octocorallia tree of life, with different terms often describing the same concepts. To address this, we introduced a hierarchical classification system that aims to facilitate the use of octocoral morphologies in ecological studies by consolidating hundreds of terms into a manageable set of ecologically meaningful morphologies. It includes seven high-level Types of Growth (Trait 1) based on spatial occupation patterns, further subdivided into 17 basic (Trait 2) and 32 detailed (Trait 3) growth forms for finer resolution (see Tables S4-S11 for detailed descriptions). For example, a coral described as “fan-shaped” by an author in the original source would be hierarchically classified as “erect branched” (Trait 1; high level), “arborescent” (Trait 2; medium level), and “branched planar” (Trait 3; fine level) under the Type of Growth, Growth Form Basic, and Growth Form Detailed traits, respectively. Additionally, a fourth trait, labeled simply as Growth Form (Trait 4), retains the original terminology from the data source to maintain traceability.

A similar challenge was encountered with the vast existing terminology for octocoral skeletons. Here, we built upon the work of McFadden *et al*.^32^ to propose a summarized classification comprising 20 basic categories that encompass the observed variation in this trait across octocorals (see accepted values for the “Type of Skeleton” trait in Table S1).

Lastly, for quantitative traits (e.g., colony height), where standardization to a preferred unit is straightforward, we did not enforce a specific unit, leaving the choice of which standard to use up to end users.

## Data Records

### Access

The OctocoralTraits v2.2 data descriptor is publicly accessible as a ZIP file deposited in Zenodo^866^. This ZIP file, named OctocoralTraits, contains a primary core table in .csv format (OctocoralTraits_v2_2.csv) with trait observations linked to measurements, along with multiple interrelated tables (also in .csv format) that provide additional details on both the observations (e.g., location, species, resource) and the measurements (e.g., trait, methodology, standard, value type, precision type). Specific information about each of these core and interrelated tables is provided in Tables 1–8.

**Table 1:** Description of the core OctocoralTraits v2.2 table containing observations binding measurements.

Variable	Value
**observation_id**	A unique ID assigned to each trait observation to link trait measurements. This ID was manually assigned during the data compilation process.
**user_id**	An ID for the user who integrated the data into the data descriptor. This ID can be cross-referenced with the user_id.csv interrelated table.
**access**	A boolean value (0; private, 1; public) indicating that all entries in the OctocoralTraits v2.2 data descriptor are public.
**specie_id** / **specie_name**	An ID / name for each octocoral species in the data descriptor, cross-referenced with the species_id.csv table. The species name follows the octocoral classification available in WoRMS as of May 2024.
**family_molecule**	The valid family name for each octocoral species, based on the latest octocoral classification derived from molecular techniques.
**location_id / location_name**	A unique ID / name for each location where trait data was collected, allowing for cross-referencing with the comprehensive list of locations in the related table location_id.csv.
**latitude / longitude**	Two columns with the coordinates of each location expressed in decimal degrees.
**resource_id**	An ID for the primary scientific source from which trait data was compiled. It can be cross-referenced with the table resource_id.csv.
**resource_secondary_id**	An ID for the secondary scientific source from which trait data was compiled, if any.
**measurement_id**	An ID for each trait measurement.
**trait_id / trait_name**	An ID / name for each trait for which data was compiled. It can be cross-referenced with the table trait_id.csv, which contains the complete list of traits and their definitions.
**trait_category**	Whether a trait is Biomechanical, Morphological, Geographical, Ecological, Reproductive, Physiological, about the conservation status of the species, or Contextual.
**standard_id / standard_unit**	An ID / name for each measurement unit used. It can be cross-referenced with the table standard_id.csv, which contains the complete list of units and their definitions.
**methodology_id / methodology_name**	An ID / name for each methodology reported to have been used to measure a trait. It can be cross-referenced with the interrelated table methodology_id.csv, which contains the complete list of methods and their description.
**value**	The actual value measured for a given trait.
**value type_id / value_type**	An ID / name for each categorical type of value used. It can be cross-referenced with the interrelated table value_type_id.csv, which contains the complete list of value types.
**precision**	The level of uncertainty associated with the value if it is made up from more than one measurement. For range, the lower value is given in this column.
**precision_type_id / precision_type_name**	An ID / name for each kind of uncertainty that the precision estimate (above) corresponds with (e.g., standard error, range…etc).
**precision_upper**	It captures the maximum (upper) value if range is used as precision_type
**replicates**	The number of measurements (replicates) that were used to calculate the value. The field is left blank when equal to one (e.g., a raw_value).
**notes**	Any relevant comment regarding a particular measurement has been included as notes.
**Original Aphia ID in publication**	The Aphia_ID code of the original species as it appeared in the publication from which the trait data was obtained. This ensures traceability of taxonomy changes, even when the combination is no longer valid, and the species has been reclassified.

**Table 2:** Structure of the Locations table, which contains details about each site where trait observations were made.

Variable	Value
**location_id**	An ID for each location in which trait data was collected. It can be cross-referenced with the location_id column from the core OctocoralTraits v2.2 table (described in Table 1). Since this data descriptor was intended to be integrated with existing data for Scleractinian corals in the Coral Trait Database, we developed a code to prevent duplicates. Specifically, whenever a location was already present, the same ID number was used in this data descriptor. For locations not previously present, new numbers were assigned in order and preceded by the letters “l” (e.g., l1, l2, l3).
**location_name**	A unique name for each location in which trait data was collected.
**latitude / longitude**	Two columns describing the latitudinal and longitudinal coordinates of each location in decimal degrees format. Except for global and regional estimates (i.e., Mediterranean Sea), locations have coordinates associated, which can be exact or representative of a larger area (e.g., Lizard Island, NE Australia) when exact coordinates are not reported in the study.

**Table 3:** Structure of the Species table, which contains details about each species for which traits were quantified.

Variable	Value
**specie_id**	An ID for each octocoral species in the data descriptor. It can be cross-referenced with the specie_id column from the core OctocoralTraits v2.2 table (described in Table 1).
**master_species**	The valid species name associated to a unique species_id. It is based on the most up to date classification of octocorals, reflected in WORMS.
**family_molecule**	The valid family name to which a given octocoral species belongs. It is based on the most up to date classification of octocorals based on molecular techniques, as reflected in WORMS.
**aphia_ID**	The aphia_ID code of each valid octocoral species present in the OctocoralTraits v2.2 data descriptor.

**Table 4:** Structure of the Resources table, which contains details about each primary and/or secondary scientific source from which trait data were compiled.

Variable	Value
**resource_id**	An ID for the scientific source from which trait data was compiled. It can be cross-referenced with the resource_id (for primary sources) and resource_secondary_id (for secondary sources) columns from the core OctocoralTraits v2.2 table (described in Table 1).
**primary_secondary**	Whether the resource_id of the previous column refers to a primary or secondary resource
**author**	The author list of the publication
**year**	The year in which the work was published
**title**	The title of the publication
**resource type**	Whether the resource is a published article, book, PhD thesis, MSc thesis, identification guide, short note, report or online datasets.
**doi_ISBN**	When available, unique digital identifier DOI associated to the publications. For public resources without DOI and open access platforms (e.g., OBIS), the URL link to the publication/platform is given. For the case of identification guides, the ISBN is given.
**resource_journal**	For works published in journals, the name of the journal.
**resource_volume_pages**	For data published in journals, the volume and pages where the data was published.

**Table 5:** Structure of the Trait table, which contains details about each measured trait.

Variable	Value
**trait_name**	The name for each trait for which data was compiled.
**traitclass_id**	Whether a trait is Biomechanical, Morphological, Geographical, Ecological, Reproductive, Physiological, about the Conservation Status of the species, or Contextual.
**trait_description**	The definition of a given trait in the data descriptor.
**accepted_values**	The values that are accepted for a given trait (see Table S1), which in the case of categorical traits may be restricted.
**trait_id**	An ID for each trait on the data descriptor. It can be cross-referenced with trait_id column from the core OctocoralTraits v2.2 table (described in Table 1). Since this data descriptor was intended to be integrated with existing data for Scleractinians in the Coral Trait Database, we developed a code to prevent duplicates. Specifically, whenever a trait was already present, the same ID number was used in this data descriptor. For traits not previously present, new numbers were assigned in order and preceded by the letters “t “(e.g., t1,t2, t3).
**editor**	The editor that is responsible for that trait.
**trait_editor_id**	An ID for each trait editor. It can be cross-referenced with the trait_editor_id.csv interrelated table, where additional information from the editors is shown.

**Table 6:** Structure of the Methodologies table, which contains details about each methodology used to quantify trait data.

Variable	Value
**methodology_id**	An ID for each methodology reported to have been used to measure a trait. It can be cross-referenced with the methodology_id column from the core OctocoralTraits v2.2 table (described in Table 1). Since this data descriptor was intended to be integrated with existing data for Scleractinians in the Coral Trait Database, we developed a code to prevent duplicates. Specifically, whenever a methodology was already present, the same ID number was used in this data descriptor. For methodologies not previously present, new numbers were assigned in order and preceded by the letters “mt “(e.g., mt1,mt2, mt3)
**methodology_name**	The specific name associated to each methodology_id
**method_description**	A brief description for each methodology.

**Table 7:** Structure of the Standards table, which contains details about the standards and units used to quantify trait data.

Variable	Value
**standard_id**	An ID for each measurement unit used. It can be cross-referenced with the standard_id column from the core OctocoralTraits v2.2 table (described in Table 1). Since this data descriptor was intended to be integrated with existing data for Scleractinians in the Coral Trait Database, we developed a code to prevent duplicates. Specifically, whenever a measurement unit was already present, the same ID number was used in this data descriptor. For measurement units not previously present, new numbers were assigned in order and preceded by the letters “st” (e.g., st1, st2, st3).
**standard_name**	The name of the standard (e.g., density, area…etc).
**Standard_unit**	A unique name for each measurement unit associated to the standard_id column.

**Table 8:** Structure of the Precision table, which contains details about the Precision estimates used to quantify the uncertainties associated to the trait measurements.

Variable	Value
**precision_type_id**	An ID for each kind of uncertainty that the precision estimate (above) corresponds with. It can be cross-referenced with the precision_type_id column from the core OctocoralTraits v2.2 table (described in Table 1).
**precision_type_name**	The name associated to each precision_type_id. Allowed values are: 95_ci, range, standard_deviation and standard_error.

### Data coverage

**Fig. 2:**
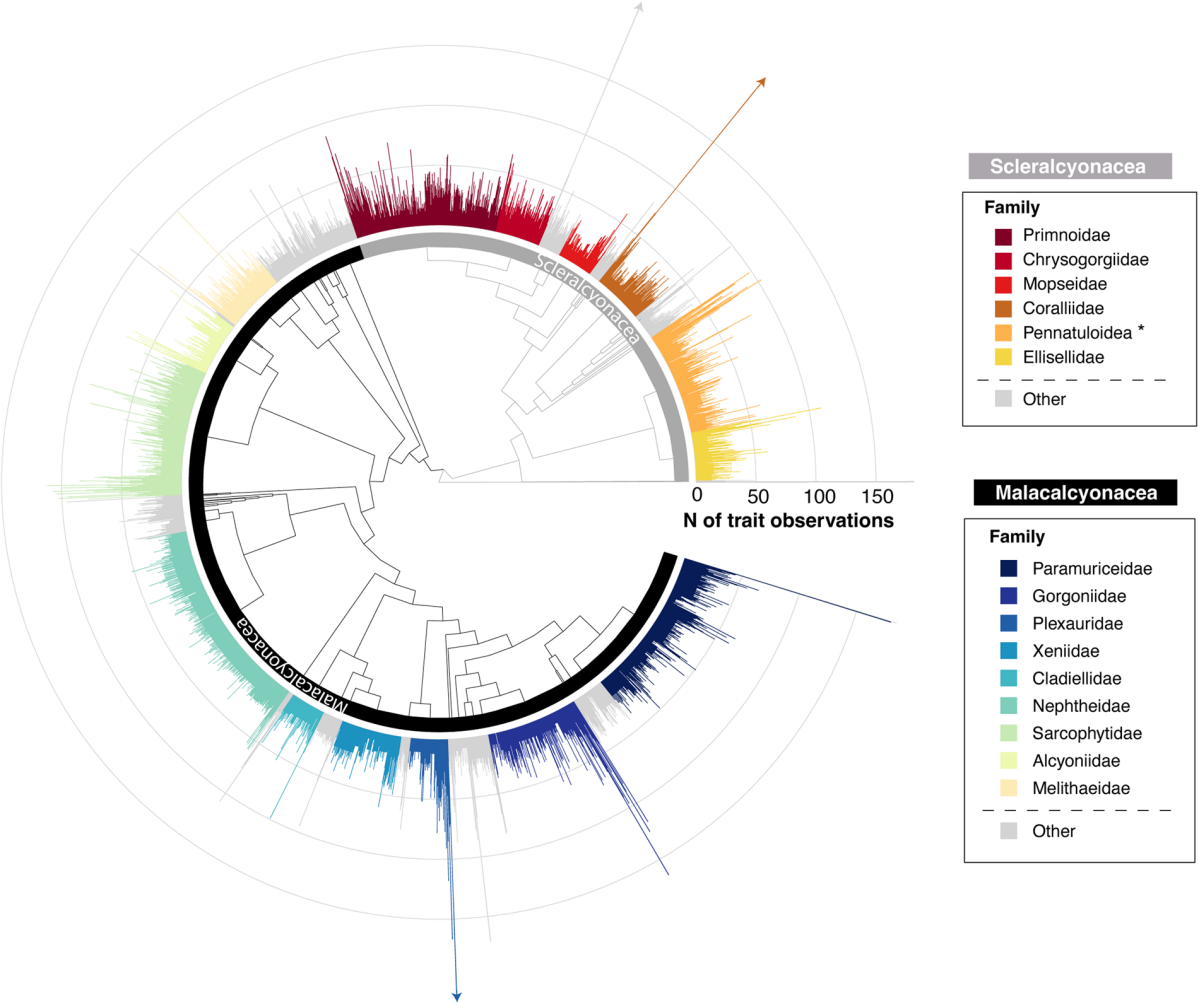
Species-complete tree with phylogenetic distribution of trait data coverage (i.e., number of trait observations per species). The 15 largest families of the Class Octocorallia are labeled by colour. The two orders within the class, Scleralcyonacea and Malacalcyonacea, are also indicated. Arrowheads indicate that, for this first data release, *Keratoisis grayi* (4708 records), *Muricea californica* (437 records) and *Corallium rubrum* (280 records) have a disproportionately larger number of trait observations that do not fit in the plot. The phylogenetic tree was added to this figure solely for visualization purposes, organizing families by evolutionary relationships to enhance the interpretability of trait distributions across lineages, without implying further phylogenetic analysis. It corresponds to an adaptation of the family-resolved Maximum likelihood tree of Octocorallia inferred from 1059 bp alignment of mitochondrial gene mtMutS^32^, with species being incorporated as polytomies. (*) To facilitate visualization, all sea pens have been grouped into a single Pennatuloidea superfamily. Data for species belonging to genera that are currently *incertae sedis* have not been included in the figure. Finally, a complementary figure showing the phylogenetic distribution of trait data coverage as the number of different traits with data per species can be found in Fig. S1.

**Fig. 3:**
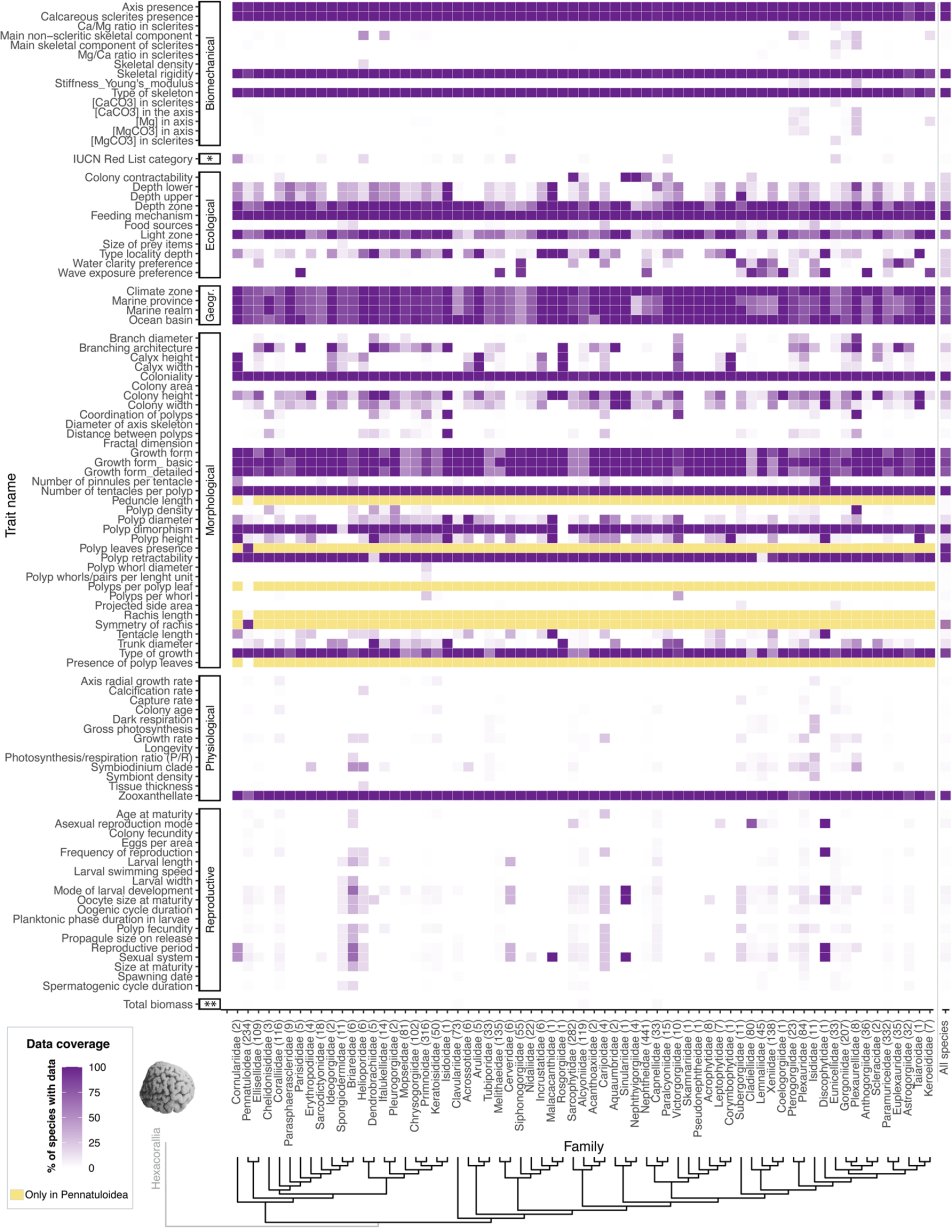
Family-complete heatmap with phylogenetic distribution of trait data coverage for each trait of interest. Parentheses following family names indicate the number of species within a given family. Purple cells correspond with the traits of interest released in this data descriptor, with color gradient indicating the % of species with data for a given trait, within a given family. Dark yellow cells indicate traits that are only possible in sea pens (e.g., the number of polyps per polyp leaf) and therefore which are not possible in other octocorals. (*) refers to Conservation trait category, while (**) refers to Stoichiometric trait category. To facilitate visualization, all sea pens have also been grouped into a single Pennatuloidea superfamily (as in Fig. 2). See Table S1 for the trait definitions and Fig. S2 for trait data coverage across genera whose family assignment is *incertae sedis*. The phylogenetic tree used to order families across the x axis corresponds to the family-resolved Maximum likelihood tree of Octocorallia inferred from 1059 bp alignment of mitochondrial gene mtMutS^32^. As for Fig. 2, it was added solely for visualization purposes, organizing families by evolutionary relationships to enhance the interpretability of trait distributions across lineages, without implying further phylogenetic analysis.

**Fig. 4:**
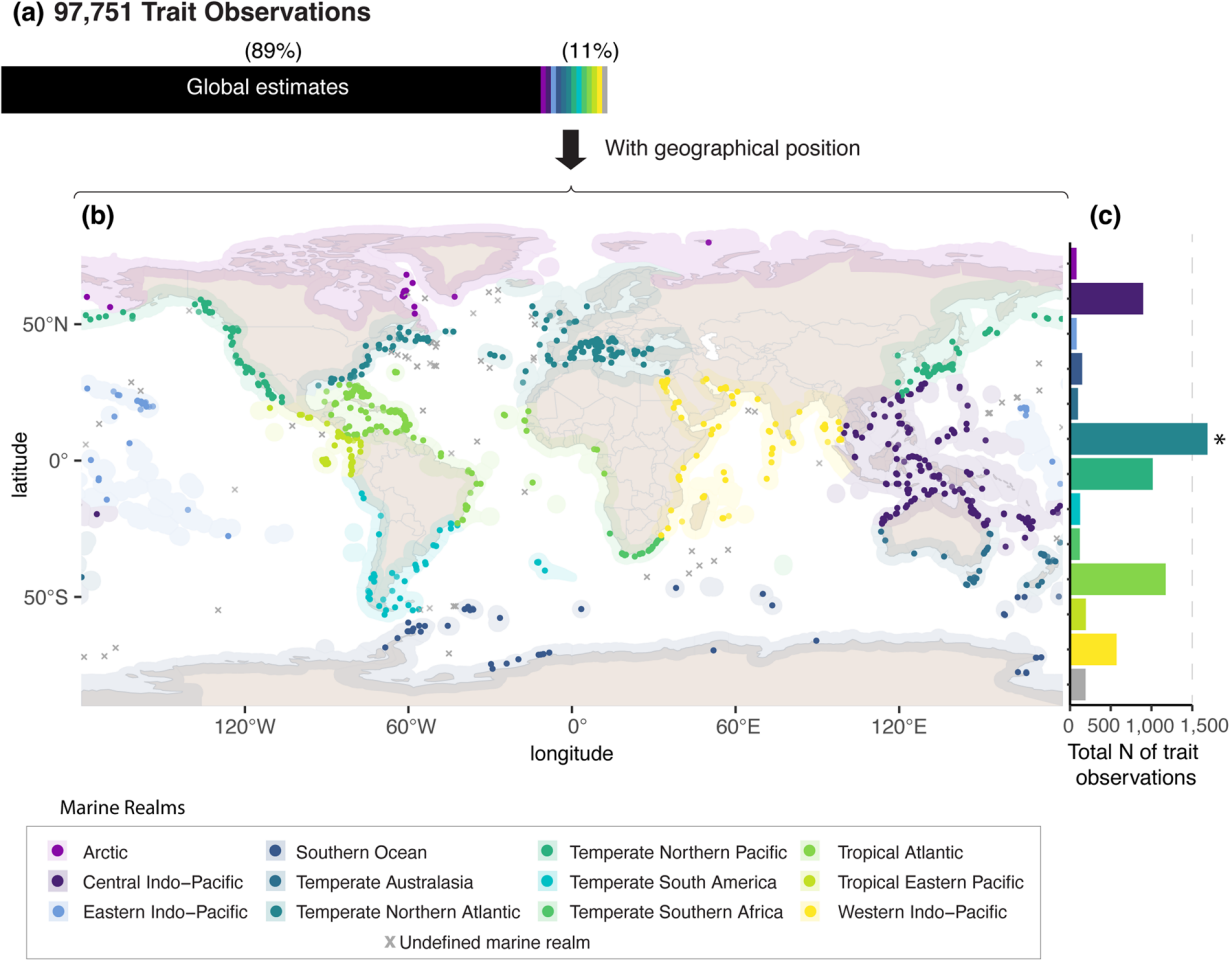
(**a**) Relative percentage of compiled trait observations among global and georeferenced estimates. (**b**) Geographic distribution of georeferenced trait data across marine realms. (**c**) Total number of trait observations per marine realm. (*) in panel c denotes that the number of trait observations for that region is higher than shown in the plot (i.e., Temperate Northern Atlantic; 5,633 records). Additional notes: As mentioned in Table 3, Global estimates are associated with traits that do not (or very rarely) vary within species regardless of the geographical context. Thus, a global estimate for a given trait can be assumed to be fairly or totally constant across the species. For instance, the presence of a skeletal axis is an inherent characteristic of gorgonians regardless of where the trait is quantified. Apart from the global estimates, OctocoralTraits v2.2 contains over 10,000 data points of geo-referenced records. These records are associated with traits that may vary within species depending on local/regional environmental parameters. Therefore, the geographic context of the trait observation is needed to provide relevant framing for analysis.

**Fig. 5:**
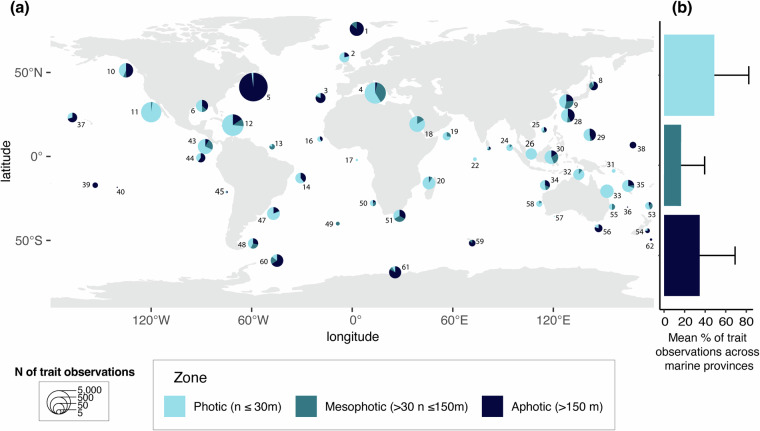
(**a**) Bathymetric (i.e., across light zones) distribution of compiled trait data across the 54 marine provinces where data has been collected. Numbers have been added to each marine province with data, corresponding exactly to the list provided by Spalding *et al*.^868^. Specifically: 1. Arctic, 2. Northern European Seas, 3. Lusitanian, 4. Mediterranean Sea, 5. Cold Temperate Northwest Atlantic, 6. Warm Temperate Northwest Atlantic, 8. Cold Temperate Northwest Pacific, 9. Warm Temperate Northwest Pacific, 10. Cold Temperate Northeast Pacific, 11. Warm Temperate Northeast Pacific, 12. Tropical Northwestern Atlantic, 13. North Brazil Shelf, 14. Tropical Southwestern Atlantic, 16. West African Transition, 17. Gulf of Guinea, 18. Red Sea and Gulf of Aden, 19. Somali/Arabian, 20. Western Indian Ocean, 22. Central Indian Ocean Islands, 24. Andaman, 25. South China Sea, 26. Sunda Shelf, 28. South Kuroshio, 29. Tropical Northwestern Pacific, 30. Western Coral Triangle, 31. Eastern Coral Triangle, 32. Sahul Shelf, 33. Northeast Australian Shelf, 34. Northwest Australian Shelf, 35. Tropical Southwestern Pacific, 36. Lord Howe and Norfolk Islands, 37. Hawaii, 38. Marshall, Gilbert, and Ellis Islands, 39. Central Polynesia, 40. Southeast Polynesia, 43. Tropical East Pacific, 44. Galapagos, 45. Warm Temperate Southeastern Pacific, 47. Warm Temperate Southwestern Atlantic, 48. Magellanic, 50. Benguela, 51. Agulhas, 53. Northern New Zealand, 54. Southern New Zealand, 55. East Central Australian Shelf, 56. Southeast Australian Shelf, 57. Southwest Australian Shelf, 58. West Central Australian Shelf, 59. Subantarctic Islands, 60. Scotia Sea, 61. Continental High Antarctic, 62. Subantarctic New Zealand. (**b**) Mean percentage (±SD) of trait observations per light zone across the 54 provinces with data.

The OctocoralTraits v2.2 data release integrates more than 97,500 error-checked trait observations and over 148,000 trait measurements across 128 traits. These data encompass over 3,500 valid octocoral species and 75 families, with observations spanning all marine ecoregions and depth zones, from deep-sea habitats to shallow waters. OctocoralTraits v2.2 therefore offers a broad phylogenetic (Figs. 2 and 3), geographic (Fig. 4), and bathymetric (Fig. 5) coverage. However, considerable data gaps persist, since many species lack comprehensive trait data (Fig. 2), some traits are underrepresented across octocoral families (e.g., growth rate) (Fig. 3), and certain realms (e.g., polar) and depth zones (e.g., mesophotic) remain poorly sampled across the scientific community (Figs. 4 and 5).

## Technical Validation

OctocoralTraits v2.2 is a curated data descriptor developed to contribute to the Coral Traits Database. Therefore, it has undergone the same editorial control and quality assurance processes established for that initiative (see https://www.coraltraits.org/procedures and Madin *et al*.^21^). Specifically, all compiled data comes from scientific sources that have been published (e.g., articles) or undergone rigorous peer review (e.g., PhD theses), ensuring that data accuracy was validated before integration. Moreover, to address potential unnoticed errors in prior validation and those that may arise during the harmonization of the data into a unified, comprehensive dataset, additional measures were taken to ensure a fully curated version for publication. First, whenever data from a new source was compiled, the .csv files were manually reviewed to confirm that all variables had the correct and intended data types. Following the finalization of data compilation, custom R scripts (available with the data descriptor on the Zenodo repository^866^) were applied to: i) detect duplicate measurements and/or observations, ii) identify inconsistencies in data structure (e.g., an observation linked to multiple species, locations, or resources), and iii) detect and flag outliers in quantitative traits and invalid values in categorical traits. Flagged values were then manually checked against the original sources by trait editors, and when necessary, original authors were consulted. Any confirmed errors identified through the technical validation process were corrected or removed, resulting in the curated OctocoralTraits v2.2 version released here.

## Usage Notes

By hosting the dataset on the public repository Zenodo, we adhere to FAIR principles^867^ and enable reuse under a CC-BY license with appropriate attribution, specifically citing this data descriptor. Additionally, the descriptor includes direct references to all original scientific sources used to compile the global harmonized dataset, found in the resource_id.csv table (also referenced in this manuscript^64–859^;). We therefore encourage users to reference the original sources whenever feasible, along with this data descriptor. Finally, the OctocoralTraits data descriptor is an evolving data product, continuously linked to ongoing data entry and validation. We encourage users to access the latest versions of OctocoralTraits files, which will be regularly updated in the same Zenodo repository^866^. Furthermore, since OctocoralTraits v2.2 is part of the Coral Trait Database collaborative effort, subsequent new versions of the dataset will also be accessible and downloadable at (www.coraltraits.org).

Despite the meticulous curation process aimed at identifying and rectifying potential errors, the inherent complexity of data acquisition and compilation may warrant an additional layer of scrutiny from end-users. It is recommended, therefore, that users apply standard validation procedures and cross-referencing methodologies to ensure the accuracy and reliability of the data for their specific analyses. This precautionary measure, which may include consulting referenced original sources or the corresponding author of this publication in case of doubt, aligns with the best practices in data utilization, reinforcing the robustness of the database for scientific inquiry and research endeavors. Finally, special caution is advised when using data from geographical traits (e.g., marine realms and provinces), particularly those derived from the OBIS platform, as not all observations on that platform have been verified for accurate species identification or up-to-date taxonomy.

## Supplementary information

Supplementary information

## Data Availability

All code used for curating the OctocoralTraits v2.2 data descriptor, as well as for generating the figures included in this manuscript is publicly available alongside the compiled data descriptor at the following Zenodo repository^866^: (10.5281/zenodo.14228404). Additionally, the code used to build the online dynamic product, which also contains data for scleractinian corals (www.coraltraits.org), can be found at (https://github.com/jmadinlab/coraltraits2).
