# Supplementary material for: The Octocoral Trait Database: a global database of trait information for octocoral species
Source: Sci Data. 2025 Jan 15;12:82. doi: 10.1038/s41597-024-04307-8 (PMC11735844; doi:10.1038/s41597-024-04307-8)
Supplement: Supplementary file 1 — Supplementary information [file 41597_2024_4307_MOESM1_ESM.pdf]

Supplementary information for the article: The Octocoral Trait Database: a global database of trait information for octocoral species

Gómez-Gras D*[1,2,3], Linares C[2,3], Viladrich N[2,3], Zentner Y[2,3] , Grinyó J[4,5], Gori A[2,3] , McFadden CS[6], Fabricius KE[7], Madin J-S[1]

1. Hawai'i Institute of Marine Biology (HIMB) , Kaneohe, Hawaii, United States
2. Departament Evolutionary Biology, Ecology and Environmental Sciences, Universitat de Barcelona (UB), Barcelona, Spain
3. Institut de Recerca de la Biodiversitat (IRBio), Universitat de Barcelona (UB), Barcelona, Spain,
4. Institut de Ciències Del Mar (ICM-CSIC), Barcelona, Spain
5. Department of Ocean System Sciences, NIOZ Royal Netherlands Institute for Sea Research and Utrecht University, Den Burg, the Netherlands
6. Department of Biology, Harvey Mudd College, Claremont, California, United States
7. Australian Institute of Marine Science, Townsville, Queensland, Australia

Supplementary Tables

- **Table S1.** Basic information (i.e., class, name, definition, variable type and accepted values) for the 98 traits of interest included in the OctocoralTraits v2.2 data descriptor
- **Table S2.** Basic information (i.e., class, name, definition, variable type and accepted values) for all contextual traits included in the OctocoralTraits v2.2 data descriptor
- **Table S3.** Reference list for all data sources across traits
- **Table S4.** Hierarchical structure of the morphological classification system proposed for octocorals by Gómez-Gras D and Fabricius K.
- **Table S5-S11:** Definition of each trait category for the Growth Form (basic) and Growth Form (detailed) traits, within each of the different octocoral Types of Growth (i.e., erect branched, erect unbranched, massive, sub-massive, horizontal unbranched, horizontal branched and solitary/pseudo-solitary).

Supplementary Figures

- **Figure S1.** Species-complete tree with phylogenetic distribution of trait data coverage as number of different traits with data per species.
- **Figure S2.** Distribution of trait data coverage across genera whose family assignation is *incertae sedis*.

Supplementary Tables

Table S1. Basic information (i.e., category, name, definition, type of variable and accepted values) for the 98 traits of interest included in the OctocoralTraits v2.2 data descriptor. The different trait categories capture various aspects of octocoral biology and ecology: **Biomechanical** (i.e., traits related to their skeletal structure and mechanics, such as the type of skeleton, which influence stability and resistance to environmental forces), **Conservation** (traits related to the quantification of their conservation status; e.g., IUCN Red List category), **Ecological** (traits that describe their environmental preferences and interactions within the ecosystem; e.g., feeding mechanism), **Geographical** (traits related to their spatial distribution; e.g., marine province), **Morphological** (traits that outline their physical appearance and structure, such as growth form), **Physiological** (traits that relate to their metabolic processes and organic functions, such as the growth and calcification rates), **Reproductive** (traits related to their reproduction), **Stoichiometric** (traits related to their elemental composition, like total biomass, providing data on the energy and nutrient content within coral tissues), and **Contextual** traits, that provide additional context to the specific trait observations. * Denotes traits that were already present in the stony-coral version of the Coral Trait Database.

**Category**	**Trait**	**Definition**	**Type of variable (in bold) and accepted values**
Biomechanical	Axis presence	Whether a species has an axis or not. The presence of an internal axis determines one of the most important features of octocoral skeletons.	**Categorical (binary)** (0 = no consolidated axis, 1= consolidated axis present)
	Calcareous sclerites presence	Whether a species has calcareous sclerites or not. The presence or absence of sclerites determines one of the most important features of octocoral skeletons.	**Categorical (binary)** (0 = no calcareous sclerites, 1= calcareous sclerites present)
	Ca/Mg ratio in sclerites	The Calcium/Magnesium ratio in sclerites.	**Numeric**
	Main non-scleritic skeletal component	The main $CaCO_3$ polymorph present in the skeleton, without considering sclerites.	**Categorical (unrestricted)**: Aragonite, Amorphous Carbonate Hydroxiapatite, High Mg Calcite, Mg Calcite…etc.
	Main skeletal component of sclerites	The main $CaCO_3$ polymorph present in sclerites. Typically, calcite.	**Categorical (unrestricted)**: Aragonite, Calcite, Vaterite
	Mg/Ca ratio in sclerites	The Magnesium/Calcium ratio in sclerites.	**Numeric**
	Skeletal density *	The material density of coral skeleton.	**Numeric**
	Skeletal rigidity	The rigidity of a coral colony/individual, based on the type of skeleton.	**Categorical (restricted)**:<br>1. Soft (i.e., species with just a hydrostatic skeleton in which the whole colony is capable of retracting or deflating or greatly changing its volume)<br>2. Semi-rigid (i.e., species with an axis in which the colony itself is not capable of retraction/deflation).<br>3. Rigid (i.e., species with a totally hard skeleton that is heavily calcified).
	Stiffness (Young's modulus)	Measures stiffness as the resistance of a material to elastic deformation under load.	**Numeric**
	Type of skeleton	The type of skeleton present in each octocoral species. The extensive nomenclature found for octocoral skeletons in the scientific literature has been harmonized into 20 categories by McFadden CS and Gómez-Gras D.	**Categorical (restricted)**:<br>1. Hard aragonite skeleton (e.g., *Heliopora*)<br>2. Axial-like layer (i.e., unconsolidated axis) formed by sclerites, with coenenchyme subdivided into medulla and cortex (e.g., *Briareum*).<br>3. Axis of consolidated but unfused sclerites (e.g., *Paragorgia*).

			4. Axis of partially fused sclerites embedded in protein (e.g., *Rosgorgia*).<br>5. Axis of fused sclerites cemented together within a solid calcareous matrix (e.g., *Corallium*).<br>6. Jointed axis with solid calcareous inter-nodes (fused or consolidated sclerites within a calcite matrix) alternating with proteinaceous nodes with sclerites (e.g., *Parisis*).<br>7. Axis of unfused sclerites embedded in protein, with hollow cross-chambered central core (*Ideogorgia)*.<br>8a. Proteinaceous axis with hollow, cross-chambered central core (unmineralized) (e.g., *Eunicella*).<br>8b. Proteinaceous axis with hollow, cross-chambered central core containing non-scleritic carbonate hydroxylapatite (CHAp) (e.g. *Gorgonia*).<br>9. Proteinaceous axis with hollow, cross chambered central core and non-scleritic calcareous loculi (e.g., *Plexaurellidae*).<br>10. Jointed axis with proteinaceous nodes that lack sclerites alternating with solid, non-scleritic calcareous internodes (e.g., *Mopsea)*.<br>11a. Solid (scleroproteinaceous) axis with radially-layered non-scleritic calcareous material (e.g., *Ellisella* and those sea pens with axis).<br>11b. Solid (scleroproteinaceous) axis with concentrically-layered non-scleritic calcareous material<br>(e.g., *Chrysogorgia*).<br>11c. Solid (scleroproteinaceous) axis with concentrically-layered non-scleritic aragonite (e.g, *Plumigorgia)*.<br>12. Solid proteinaceous axis (unmineralized), colony lacking sclerites or other calcareous material (e.g., *Trichogorgia capensis*).<br>13. No axis, no sclerites, tissue covered by thin periderm or cuticular envelope (e.g.,*Cornularia*).<br>14. No axis, unconsolidated sclerites embedded in tissue (e.g., *Alcyonium*).<br>15. No axis, longitudinally disposed sclerites surround canals to form rigid branches (e.g., *Siphonogorgia*).<br>16. No axis, calcified structures (e.g., polyp tubes, branches and/or membranes) derived of fused sclerites (e.g., *Tubipora*).<br>17. No axis, no sclerites, tissue without periderm or cuticular envelope (e.g., *Altumia*)
	[CaCO3] in sclerites	The concentration of Calcium Carbonate in sclerites.	**Numeric**
	[CaCO3] in axis	The concentration of Calcium Carbonate in the skeletal axis.	**Numeric**
	[Mg] in axis	The concentration of Magnesium in the axis.	**Numeric**
	[MgCO3] in axis	The concentration of Magnesium Carbonate in the skeletal axis.	**Numeric**
	[MgCO3] in sclerites	The concentration of Magnesium Carbonate in sclerites.	**Numeric**
Conservation	IUCN Red List Category*	Red list categories as defined by the IUCN Red List of Threatened Species.	**Categorical (restricted)**: Least Concern (LC), Near Threatened (NT), Vulnerable (Vu), Endangered (EN), Critically Endangered (CR), Data Deficient (DD)
Ecological	Colony contractability	Some soft octocoral species can contract at the colony level.	**Categorical (binary)** (0= No, 1 = Yes)
	Depth lower*	The maximum (deepest) observed depth of a species. Data are a mix of individual-level local observations and species-level global estimates based on expert opinion.	**Numeric**

	Depth upper*	The minimum (shallowest) observed depth of a species. Data are a mix of individual-level local observations and species-level global estimates based on expert opinion.	**Numeric**
	Depth zone	The depth zone in which a species is typically found.	**Categorical (restricted)**: Shallow (0 to 50 m), Moderately deep (51 to 200 m), Deep (> 200 m)
	Feeding mechanism	The mechanism used by each octocoral species to obtain food.	**Categorical (restricted)**: Suspension feeder. For future descriptor versions, new subcategories may be added to provide more detail.
	Food sources	Food sources of octocoral species.	**Categorical (unrestricted)**: POM, diatoms, zooplankton…etc.
	Light zone	The light zone where the species can be found, in relation to depth.	**Categorical (restricted)**: Photic (0 to 30 m), Mesophotic (31 to 150 m), Aphotic (deeper than 150 m)
	Size of prey items	The size of the prey of a coral.	**Numeric**
	Type locality depth	The depth at which a type specimen of a species (typically the holotype) was collected.	**Numeric**
	Water clarity preference*	Preferred water clarity environment. Derived mostly from expert opinions (e.g., Fabricius and Alderslade, 2001).	**Categorical (restricted)**:<br>1. Clear (i.e., the species are mostly found in clear, transparent waters).<br>2. Turbid (i.e., the species is mostly found in turbid waters)<br>3. Both (i.e., the species is typically found in either clear or turbid environments).
	Wave exposure preference*	Preferred hydrodynamic exposure environment (e.g., Fabricius and Alderslade, 2001).	**Categorical (restricted)**:<br>1. Protected (i.e., the species is typically found in protected enviroments where the impact of waves is none or minimum).<br>2. Exposed (i.e., the species is found mostly in exposed environments where the action of waves can be intense).<br>3. Broad (i.e., the species can be found in either protected or exposed environments).
Geographical	Climate zone	The climatic zone where an octocoral species can be found. Species can have multiple climatic zones associated.	**Categorical (restricted)**: Polar, Temperate, Tropical
	Marine province	The marine province (as defined by Spalding et al. 2007) in which a species can be found. Caution should be taken when using these data, especially when derived from online platforms such as OBIS, where not all observations have been verified to match correct species or taxonomy updates.	**Categorical (restricted)**: Complete list of marine provinces can be found in Spalding et al. 2007
	Marine realm	The marine realm (as defined by Spalding et al. 2007) in which a species can be found. Caution should be taken when using these data, especially when derived from online platforms such as OBIS, where not all observations have been verified to match correct species or taxonomy updates.	**Categorical (restricted)**: Complete list of marine realms can be found in Spalding et al. 2007
	Ocean basin*	The ocean basin in which a species is found. Indian and Pacific Oceans are grouped as "pacific". Caution should be taken when using these data, especially when derived from online platforms such as OBIS, where not all observations have been verified to match correct species or taxonomy updates.	**Categorical (restricted)**: Atlantic, Pacific
Morphological	Branch diameter	In a branched colony, the diameter of branches.	**Numeric**
	Branching architecture	The branching pattern in branched colonies.	**Categorical (unrestricted)**: e.g., Net-like, dichotomous, pseudo-dichotomous, pinnate, irregular, leaf-like, lobate, glomerate…etc.
	Calyx height	In octocoral species with calyces, the height of the calyx that contains the polyp.	**Numeric**
	Calyx width	In octocoral species with calyces, the diameter of the calyx that contains the polyp.	**Numeric**
	Coloniality*	Whether mature individuals of a species are colonial or solitary	**Categorical (restricted)**: Colonial, Solitary.
	Colony area*	The size of a colony. Typically associated with a trait that is expected to vary with colony size. The default is planar projected area.	**Numeric**
	Colony height*	The height of a coral colony from the bottom to the top.	**Numeric**
	Colony width*	The width of a coral colony	**Numeric**

	Coordination of polyps	This trait gives information about how polyps are distributed within the colony.	**Categorical (unrestricted)**: In whorls, isolated, in pairs, bi-serially, in clumps…etc.
	Diameter of axis skeleton	The axis diameter in species with internal axis	**Numeric**
	Distance between polyps	The distance between two polyps in a coral colony. Typically measured as the distance between the centers of the two polyps (or calyces).	**Numeric**
	Fractal dimension	A geometrical index of colony complexity based in measures of the change in rugosity with changing scale of measurement.	**Numeric**
	Growth form*	The growth form of a coral colony measured in the field or during an experiment. The range of growth form categories is not constrained (i.e., growth forms should be named as they were in the original resource or dataset), and therefore the number of categories will become large.	**Categorical (unrestricted)**
	Growth form (broad) (Gómez-Gras and Fabricius)	A subclassification of the Type of growth trait into broad groups depending on basic morphological features such as the presence of stem/stalk, the presence of stolons, lobes…etc. * The specific definition used for each growth form (broad) category can be found in Tables S4-S11.	**Categorical (restricted)**:<br>1.1. Arborescent<br>1.2. Erect branched (tangled)<br>1.3. Feather like<br>2.1. Erect unbranched (simple)<br>2.2. Erect unbranched (capitate)<br>3.1. Massive<br>4.1. Sub-massive (lobate-digitate)<br>4.2. Sub-massive (columnar)<br>4.3. Sub-massive (branched)<br>4.4. Sub-massive (foliose)<br>4.5 Sub-massive (with domed capitulum)<br>4.6. Sub-massive (plate-like)<br>5.1. Membranous (sheet-like)<br>5.2. Encrusting<br>6.1 Encrusting (stoloniferous)<br>6.2. Horizontal branched with axial skeletal support<br>7.1 Solitary/Pseudo-solitary
	Growth form (detailed) (Gómez-Gras & Fabricius)	A subclassification of the Growth form (broad) trait in which the coarse morphological groups are further subdivided into more detailed groups depending on complex morphological features such as the plane and type of branching, type of capitulum etc. * The specific definition used for each growth form (constrained) category can be found in Tables S4-S11.	**Categorical (restricted)**:<br>1.1.1. Branched planar (simple)<br>1.1.2. Branched planar (multi-plane)<br>1.1.3. Bushy/Tree-like (TYPE: rigid/semi-rigid)<br>1.1.4. Bushy/Tree-like (TYPE: soft)<br>1.1.5. Bottlebrush<br>1.1.6. Sparsely branched<br>1.1.7. Arborescent with terminal branching<br>1.1.8. Arborescent lobate<br>1.1.9. Arborescent with domed capitula<br>1.2.1. Erect branched tangled (stoloniferous)<br>1.2.2. Erect branched tangled (with axial skeletal support)<br>1.3.1. Feather-like<br>2.1.1. Flagelliform/filiform<br>2.1.2. Erect unbranched (pen-like)<br>2.1.3. Digitiform<br>2.2.1. Erect unbranched (with domed capitulum)<br>2.2.2. Erect unbranched (with disc-shaped capitulum)<br>2.2.3. Erect unbranched (with terminal cluster of polyps)<br>3.1.1. Massive<br>4.1.1. Sub-massive (lobate/digitate)<br>4.2.1. Sub-massive (columnar)<br>4.3.1. Sub-massive (branched)<br>4.4.1. Sub-massive (foliose)<br>4.5.1. Sub-massive (with domed capitulum)<br>4.6.1. Sub-massive (plate-like)<br>5.1.1. Membranous (sheet-like)<br>5.2.1. Encrusting (simple)<br>5.2.2. Encrusting (digitate/lobate/folded)<br>5.2.3. Encrusting (with verrucae)<br>6.1.1. Encrusting (stoloniferous)<br>6.2.1. Horizontal branching with axial skeletal support<br>7.1.1. Solitary/Pseudo-solitary

	Number of pinnules per tentacle	The number of pinnules in octocoral tentacles	**Numeric**
	Number of tentacles per polyp	The number of tentacles per polyp	**Numeric**
	Peduncle length	The length of the peduncle in sea pens	**Numeric**
	Polyp density	The number of polyps per length or area units.	**Numeric**
	Polyp diameter	The diameter of the polyp. Different authors give different measurements and proxies for this trait (e.g., some report the size of the contracted polyps, while others report the size of the entire polyp, including tentacles, or do not specify it). Thus, observation notes in the data descriptor and original sources should be checked when using this trait for comparative analyses among species.	**Numeric**
	Polyp dimorphism	If the colony has one type of polyp or more	**Binomial** (0= monomorphic polyps, 1= dimorphic polyps) * The few species with trimorphic polyps are included in type 1).
	Polyp height	The height of the polyp (sometimes referred as length). Different authors give different measurements and proxies for this trait (e.g., some report the size of the contracted polyps, whereas others report the size of the entire polyp, including tentacles). In some cases, this information is not given. Number of measured replicates is often lacking as well. Thus, observation notes in the database and original sources should be checked when using this trait for comparative analyses among species.	**Numeric**
	Polyp retractability	Whether polyps can retract into the coenenchyme or not.	**Binomial** (0= non-retractable, 1= retractable)
	Polyp whorls/pairs per length unit	The number of polyp whorls or pairs per length unit.	**Numeric**
	Polyps per polyp leaf	In sea pens with polyp leaves, the number of polyps in each polyp leaf.	**Numeric**
	Polyps per whorl	In species with polyp whorls, the number of polyps in each whorl	**Numeric**
	Polyp leaves presence	For sea pens only. Whether a species has polyp leaves or not.	**Binomial** (0=polyp leaves absent, 1= polyp leaves present)
	Projected side area	The projected area when seen from the side, perpendicular to the main growth direction of the colony.	**Numeric**
	Rachis length	The length of the rachis in sea pens.	**Numeric**
	Symmetry of rachis	For sea pens only. Whether a species has a symmetrical rachis or not.	**Categorical (restricted)**: Radial, Bilateral
	Tentacle length	The length of the polyp tentacles	**Numeric**
	Trunk diameter	Diameter of the basal trunk in octocorals, including both axis skeleton and tissue. For simplicity, we use the word trunk to refer also to stems, stalks and rods.	**Numeric**
	Type of growth (Gómez-Gras & Fabricius)	Basic classification system in which the very diverse growth forms described in the scientific literature are classified into very coarse groups depending on space occupation patterns (erect, horizontal, massive/submassive) and the presence/absence of branches. * The specific definition used for each Type of growth category can be found in Table S4.	**Categorical (restricted)**:<br>1. Erect branched<br>2. Erect unbranched<br>3. Massive<br>4. Submassive<br>5. Horizontal unbranched<br>6. Horizontal branched<br>7. Solitary/Pseudo-solitary
	Polyp whorl diameter	In species with whorls, the diameter of the whorl	**Numeric**
Physiological	Axis radial growth rate	The axial radial growth rate in species with axis	**Numeric**
	Calcification rate*	The rate at which $CaCO3$ (i.e., aragonite or calcite) is laid down per unit of skeletal surface area. When using this data, be aware that this trait is measured in numerous ways.	**Numeric**
	Capture rate	The capture rate of coral colonies/individuals.	**Numeric**
	Colony age	The age of the colony at the time of measurement and/or dead.	**Numeric**
	Dark respiration*	The rate of oxygen consumption measured in the darkness per unit of skeletal surface area. Values may come from both light enhanced and dark acclimated dark respiration.	**Numeric**

	Gross photosynthesis*	The rate of oxygen production measured in the light per unit of skeletal surface area. This includes oxygen consumption due to light respiration.	**Numeric**
	Growth rate*	Typically, the yearly extension for branching and massive corals, or simple linear extension. Growth rate is sometimes measured using different dimensions (e.g., diameter and radius of the colony) or over shorter periods of time (e.g., month), which are indicated by measurement standards and methodologies, and so values may need to be standardised before comparisons among measurements can be made.	**Numeric**
	Longevity	The life-expectancy of a given species	**Numeric**
	Photosynthesis/ respiration ratio (P/R)*	Defined as ratio of net oxygen produced: oxygen produced by photosynthesis (P) to oxygen released in total ecosystem respiration (R)	**Numeric**
	Symbiodinium clade*	The genetic identity of Symbiodinium found in coral tissue at the clade level (broad level of major symbiont taxa). This is typically identified using regions of the nuclear ribosomal DNA, but other regions are also used.	**Categorical (unrestricted)**: e.g., A, B, C, D, G…etc.
	Symbiont density*	The number of symbionts per polyp or unit surface	**Numeric**
	Tissue thickness*	The distance from the external surface to the internal surface of the coral tissue.	**Numeric**
	Zooxanthellate*	Is the species zooxanthellate?	**Categorical (binary)** (0= No, 1=Yes)
Reproductive	Age at maturity*	The age at which a coral is observed to start producing gametes and spawning.	**Numeric**
	Asexual reproduction mode	The mode of asexual reproduction observed in a species, if any.	**Categorical (unrestricted)**: e.g., Polyp bail-out, colony fission, fragmentation, budding, autotomy, parthenogenesis, runners…etc.
	Colony fecundity*	It can be estimated in different ways, such as the sum of egg and testes volume per polyp multiplied by the total number of polyps, or by estimating the number of eggs in colonies.	**Numeric**
	Eggs per area*	The number of oocytes per surface unit.	**Numeric**
	Frequency of reproduction	Whether species reproduce following annual cycles or in a continuous manner.	**Categorical (restricted)**: Annual, continuous
	Larval length	The length of the larvae.	**Numeric**
	Larval swimming speed*	The swimming speed, typically the maximum, of coral larvae.	**Numeric**
	Larval width	The width of the larvae.	**Numeric**
	Mode of larval development*	The mode of larval development classified as either a brooder, broadcast spawner or surface brooder.	**Categorical (restricted)**:<br>1. Brooder (i.e., fertilization is internal and colonies release planulae larvae)<br>2. Surface Brooder (i.e., gametes are released before fertilization, but remain on the colony surface of the female until they are fertilized and brooded).<br>3. Broadcast spawner (i.e., gametes are released for external fertilization and the planulae develop in the plankton).
	Oocyte size at maturity *	The diameter of mature oocytes in a population. Determined by histology or dissection or measuring the size of eggs once released from the colony in broadcast spawners.	**Numeric**
	Oogenic cycle duration	The duration of the oogenic cycle. For stony corals, this trait appears as length oogenic cycle max.	**Numeric**
	Planktonic phase duration in larvae	The duration of the planktonic phase in larvae.	**Numeric**
	Polyp fecundity*	The number or volume of eggs (oocytes) or larvae in a polyp.	**Numeric**
	Propagule size on release*	The size of eggs or planula larvae on release.	**Numeric**
	Reproductive period	The breeding and/or spawning period of a coral	**Categorical (unrestricted)**: e.g., May to June, Winter…etc.
	Sexual system*	Whether polyps within a single colony of a given species can produce gametes of only one sex or of both sexes at the same time.	**Categorical (restricted)**:<br>1. Gonochoric (i.e., a species that presents colonies having polyps with gametes that are only from one sex (either male or female), but not both within the same colony).

			2. Hermaphroditic (i.e., a species that presents colonies in which different polyps can bear both female and male gametes).<br>3. Mixed sexuality (i.e., a species where colonies can be either gonochoric or hermaphroditic).<br>4. Gonochoric with rare hermaphroditism (i.e., a species where colonies are typically gonochoric, rarely hermaphroditic).
	Size at maturity*	The colony size, typically height or diameter at which a coral is observed to start reproducing.	**Numeric**
	Spawning date*	The date a coral colony was observed to spawn.	**Date** in format Day/Month/Year
	Spermatogenic cycle duration *	The duration of the spermatogenic cycle. For stony corals, this trait appears as length spermatogenic cycle.	**Numeric**
Stoichiometric	Total biomass*	The dry weight of holobiont tissue, typically reported as mass per unit of skeletal surface area of a colony.	**Numeric**

Table S2. Basic information (i.e., class, name, definition, type and accepted values) for all contextual traits included in the OctocoralTraits v2.2 data descriptor * Denotes traits that were already present in the stony-coral version of the Coral Trait Database.

Category	Trait	Definition	Type of trait (in bold) and accepted values
Contextual	Water depth *	The water depth at which a coral trait was measured.	**Numeric**
	Water temperature*	Water temperature is a contextual trait of the environment in which a coral trait is measured.	**Numeric**
	Year *	The year in which an observation took place. The month or season in which an observation took place should be added separately.	**Numeric**
	Month *	The month of the year in which an observation took place. The year in which an observation took place should be added separately.	**Numeric**
	[Dissolved oxygen] *	The oxygen concentration in the water where the trait of interest was quantified.	**Numeric**
	[Phosphate]*	The phosphate concentration in the water where the trait of interest was quantified.	**Numeric**
	Salinity*	The salinity in the water where the trait of interest was quantified.	**Numeric**
	Colony bleached*	If measurements were taken of a bleached colony.	**Numeric**
	Type of branch	The type of branch (new) or (old) can affect the growth rate.	**Categorical (restricted):** New, old.
	Number of clipped branches	The number of branches that have been clipped in a colony to simulate mortality, which potentially affects growth rate.	Numeric
	Branch order	The order of the branch may affect growth rate	**Categorical (unrestricted):** First, second, third…etc.
	Pollution level	Pollution may affect growth rates of species	**Categorical (restricted):** Low, High
	Flow rate*	Flow rate is a contextual trait of the environment in which a coral trait is measured.	**Numeric**
	Branch age class	In organisms with determinate modular growth, the age of branches may influence their growth.	**Categorical (restricted)**: 1 to 5
	[Silicate]	The concentration of silicate in the waters where the trait of interest was quantified.	**Numeric**

	pH	The acidity of the water where the trait of interest was quantified	**Numeric**
	Previously harvested	Whether measurements were taken in a previously harvested site or colony	**Binomial** (1= Yes, 0=No)
	Habitat type	The habitat type in which the measurements were taken.	**Categorical (unrestricted):** Any level can be added to: overhangs, cave interior, cave entrance, boulders, cobble, mud-sand, gravel, outcrop, wall, coralligenous concretion, soft bottom…etc.
	Colony gender	Whether measurements were taken in female, male or hermaphroditic colonies.	**Categorical (restricted):** Male, Female, Hermaphroditic
	Protection level	Whether a site in which trait measurements were taken was unprotected or protected (e.g., MPA).	**Categorical (restricted):** Unprotected, MPA
	Colony ID	The ID given to a colony whose traits have been measured	**Categorical (unrestricted):** Any given ID label could be included
	Oil spill impact	This contextual trait specifies if the trait measurements were taken in an impacted or unimpacted location by oil spills.	**Categorical (restricted)**: Impacted, unimpacted
	Years since harvest	The years that have happened since harvest of the coral colonies	**Numeric**
	Polyp type	In species with polyp dimorphism/trimorphism or axial/lateral colony configuration, the type of polyp that has been measured/examined.	**Categorical (unrestricted):** Siphonozooid, autozooid, central autozooid, lateral autozooid, mesozooid, primary (axial), secondary (lateral).
	Season	The season in which a trait was measured.	**Categorical (restricted):** Summer, Autumn, Winter, Spring
	Total number of polyps	The total number of polyps in a colony.	**Numeric**
	Prey concentration	The concentration of prey within the water.	**Numeric**
	Date	The date on which a trait was measured	**Date**
	Period of the day	The period of the day in which a trait was measured.	**Categorical (restricted):** Daytime, night
	Health status of the reef	Whether the reef in which a trait was quantified was in a healthy condition or not.	**Categorical (restricted):** Healthy, Degraded

Table S3. Reference list for all data sources across traits

Category	Trait	Resource_id at OctocoralTrait v2.2 (Reference number in the manuscript text)
Biomechanical	Axis presence	r497(526), r498(527), r640(669), r653(682), r836(859), r657(686), r697(726), r725(754), r800(824), r817(32), r124(165), r835(858)
	Calcareous sclerites presence	r17(75), r132(173), r136(177), r147(188), r158(198), r166(205), r170(209), r175(214), r291(325), r314(345), r331(362), r334(365), r333(364), r336(367), r337(368), r338(369), r339(370), r340(371), r345(376), r343(374), r344(375), r346(377), r348(379), r349(380), r350(381), r351(382), r352(383), r353(384), r354(385), r355(386), r356(387), r357(388), r358(389), r359(390), r360(391), r361(392), r362(393), r363(394), r364(395), r365(396), r366(397), r367(398), r368(399), r369(400), r370(401), r371(402), r375(406), r380(411), r378(409), r389(420), r394(424), r396(426), r397(427), r401(431), r402(432), r409(439), r410(440), r411(441), r412(442), r413(443), r416(446), r424(454), r425(455), r426(456), r430(460), r434(464), r435(465), r436(466), r438(468), r439(469), r440(470), r441(471), r442(472), r444(474), r445(475), r447(477), r448(478), r450(479), r451(480), r452(481), r453(482), r454(483), r455(484), r456(485), r457(486), r458(487), r459(488), r460(489), r461(490), r463(492), r464(493), r465(494), r466(495), r467(496), r468(497), r469(498), r470(499), r471(500), r473(502), r474(503), r475(504), r476(505), r477(506), r478(507), r479(508), r480(509), r481(510), r482(511), r483(512), r484(513), r485(514), r486(515), r821(848), r487(516), r488(517), r489(518), r490(519), r491(520), r492(521), r493(522), r494(523), r495(524), r496(525), r497(526), r498(527), r499(528), r500(529), r501(530), r502(531), r503(532), r504(533), r505(534), r506(535), r507(536), r508(537), r509(538), r510(539), r511(540), r512(541), r513(542), r514(543), r790(817), r830(857), r831(858), r832(859), r515(544), r516(545), r517(546), r518(547), r519(548), r520(549), r521(550), r522(551), r523(552), r524(553), r525(554), r526(555), r527(556), r528(557), r529(558), r530(559), r531(560), r532(561), r533(562), r534(563), r535(564), r536(565), r537(566), r538(567), r539(568), r540(569), r541(570), r542(571), r543(572), r544(573), r545(574), r547(576), r548(577), r549(578), r550(579), r551(580), r552(581), r554(583), r555(584), r556(585), r557(586), r558(587), r559(588), r560(589), r561(590), r562(591), r563(592), r564(593), r565(594), r566(595), r567(596), r568(597), r569(598), r570(599), r571(600), r572(601), r573(602), r574(603), r575(604), r576(605), r577(606), r578(607), r579(608), r580(609), r581(610), r582(611), r583(612), r584(613), r585(614), r586(615), r587(616), r588(617), r589(618), r590(619), r591(620), r592(621), r593(622), r594(623), r595(624), r596(625), r597(626), r598(627), r599(628), r600(629), r601(630), r602(631), r603(632), r604(633), r606(635), r607(636), r608(637), r609(638), r610(639), r611(640), r612(641), r613(642), r614(643), r615(644), r616(645), r617(646), r618(647), r619(648), r620(649), r621(650), r622(651), r623(652), r625(654), r626(655), r627(656), r628(657), r631(660), r632(661), r633(662), r634(663), r635(664), r636(665), r637(666), r638(667), r640(669), r642(671), r643(672), r644(673), r646(675), r647(676), r648(677), r649(678), r650(679), r651(680), r655(684), r656(685), r657(686), r658(687), r659(688), r660(689), r662(691), r663(692), r664(693), r665(694), r666(695), r667(696), r668(697), r669(698), r670(699), r671(700), r672(701), r673(702), r674(703), r676(705), r677(706), r685(714), r690(719), r691(720), r692(721), r693(722), r695(724), r697(726), r698(727), r725(754), r731(759), r734(762), r736(763), r740(767), r742(769), r747(774), r749(776), r797(821), r808(832), r809(833), r811(835), r812(836), r813(837), r814(838), r815(839), r816(840), r817(32), r124(165), r835(858), r446(476)
	Ca/Mg ratio in sclerites	r773(799), r833(856)
	Main non-scleritic skeletal component	r698(727), r785(809), r804(828), r807(831), r817(32), r798(822)
	Main skeletal component of sclerites	r694(723), r759(786), r773(799), r777(802), r778(803), r783(807), r811(835), r798(822)
	Mg/Ca ratio in sclerites	r783(807), r784(808)
	Skeletal density	r88(136)
	Skeletal rigidity	r439(469), r440(470), r640(669), r817(32)
	Stiffness (Young's modulus)	r781(805), r804(828), r807(831)
	Type of skeleton	r316(347), r333(364), r340(371), r341(372), r342(373), r343(374), r344(375), r345(376), r346(377), r348(379), r349(380), r350(381), r351(382), r352(383), r353(384), r354(385), r356(387), r357(388), r358(389), r359(390), r360(391), r363(394), r364(395), r365(396), r366(397), r368(399), r369(400), r370(401), r371(402), r375(406), r378(409), r820(843), r389(420), r401(431), r402(432), r412(442), r413(443), r424(454), r425(455), r430(460), r434(464), r435(465), r436(466), r438(468), r439(469), r440(470), r441(471), r444(474), r469(498), r502(531), r517(546), r532(561), r538(567), r568(597), r586(615), r628(657), r633(662), r638(667), r640(669), r648(677), r652(681), r657(686), r667(696), r697(726), r698(727), r725(754), r800(824), r805(829), r817(32), r653(682), r124(165)
	[CaCO3] in sclerites	r773(799)
	[CaCO3] in axis	r778(803), r804(828), r807(831)

	[Mg] in axis	r785(809), r804(828)
	[MgCO3] in axis	r804(828), r807(831)
	[MgCO3] in sclerites	r15(73), r773(799), r783(807)
Conservation	IUCN Red List Category	r373(404), r822(841)
Ecological	Colony contractability	r333(364), r436(466), r451(480)
	Depth lower	r17(75), r39(97), r52(109), r60(112), r76(124), r77(125), r81(129), r102(148), r122(163), r133(174), r144(185), r147(188), r148(189), r149(190), r94(141), r135(176), r166(205), r171(210), r193(231), r194(232), r201(239), r222(259), r225(262), r226(263), r228(265), r235(272), r240(277), r259(295), r264(299), r331(362), r332(363), r336(367), r337(368), r338(369), r339(370), r340(371), r341(372), r342(373), r333(364), r343(374), r345(376), r346(377), r348(379), r349(380), r350(381), r351(382), r352(383), r353(384), r354(385), r356(387), r357(388), r358(389), r359(390), r360(391), r361(392), r362(393), r363(394), r364(395), r365(396), r366(397), r367(398), r368(399), r369(400), r370(401), r371(402), r372(403), r379(410), r380(411), r381(412), r382(413), r383(414), r384(415), r385(416), r401(431), r402(432), r405(435), r407(437), r409(439), r410(440), r411(441), r412(442), r413(443), r415(445), r416(446), r419(449), r797(824), r422(452), r425(455), r430(460), r432(462), r442(472), r448(478), r452(481), r470(499), r450(479), r451(480), r472(501), r473(502), r474(503), r475(504), r476(505), r485(514), r487(516), r489(518), r490(519), r491(520), r492(521), r493(522), r494(523), r496(525), r505(534), r506(535), r509(538), r510(539), r513(542), r790(814), r830(853), r831(854), r832(855), r515(544), r518(547), r520(549), r521(550), r522(551), r523(552), r524(553), r532(561), r536(565), r537(566), r539(568), r544(573), r547(576), r551(580), r554(583), r557(586), r558(587), r559(588), r562(591), r563(592), r566(595), r569(598), r577(606), r579(608), r581(610), r582(611), r586(615), r587(616), r588(617), r589(618), r591(620), r593(622), r594(623), r597(626), r598(627), r599(628), r600(629), r601(630), r602(631), r603(632), r604(633), r606(635), r613(642), r617(646), r618(647), r619(648), r623(652), r625(654), r626(655), r627(656), r628(657), r637(666), r638(667), r640(669), r648(677), r649(678), r650(679), r655(684), r657(686), r659(688), r663(692), r664(693), r665(694), r666(695), r667(696), r672(701), r673(702), r677(706), r678(707), r681(710), r685(714), r688(717), r690(719), r692(721), r693(722), r697(726), r719(748), r721(750), r729(757), r730(758), r731(759), r740(767), r755(782), r775(800), r792(816), r793(817), r808(832), r812(836), r813(837), r814(838), r816(840), r820(843), r124(165), r256(292), r255(291)
	Depth upper	r17(75), r39(97), r52(109), r60(112), r77(125), r81(129), r102(148), r122(163), r133(174), r144(185), r147(188), r148(189), r149(190), r94(141), r135(176), r166(205), r171(210), r193(231), r194(232), r201(239), r222(259), r225(262), r226(263), r228(265), r235(272), r240(277), r259(295), r264(299), r331(362), r332(363), r336(367), r337(368), r338(369), r339(370), r341(372), r342(373), r333(364), r343(374), r345(376), r346(377), r348(379), r349(380), r350(381), r351(382), r352(383), r353(384), r354(385), r356(387), r357(388), r358(389), r359(390), r360(391), r361(392), r362(393), r363(394), r364(395), r365(396), r366(397), r367(398), r368(399), r369(400), r370(401), r371(402), r372(403), r379(410), r380(411), r382(413), r383(414), r384(415), r385(416), r386(417), r387(418), r388(419), r401(431), r402(432), r405(435), r407(437), r409(439), r410(440), r411(441), r412(442), r413(443), r415(445), r416(446), r419(449), r797(824), r422(452), r425(455), r430(460), r432(462), r442(472), r448(478), r452(481), r470(499), r450(479), r451(480), r472(501), r473(502), r474(503), r475(504), r476(505), r485(514), r487(516), r489(518), r490(519), r491(520), r492(521), r493(522), r494(523), r496(525), r505(534), r506(535), r509(538), r510(539), r513(542), r790(814), r830(853), r831(854), r832(855), r515(544), r518(547), r520(549), r521(550), r522(551), r523(552), r524(553), r532(561), r536(565), r537(566), r539(568), r544(573), r547(576), r551(580), r554(583), r557(586), r558(587), r559(588), r562(591), r563(592), r566(595), r569(598), r577(606), r579(608), r581(610), r582(611), r586(615), r587(616), r588(617), r589(618), r591(620), r593(622), r594(623), r597(626), r598(627), r599(628), r600(629), r601(630), r602(631), r603(632), r604(633), r606(635), r613(642), r617(646), r618(647), r619(648), r623(652), r625(654), r626(655), r627(656), r628(657), r637(666), r638(667), r640(669), r648(677), r649(678), r650(679), r655(684), r657(686), r659(688), r663(692), r664(693), r665(694), r666(695), r667(696), r672(701), r673(702), r677(706), r678(707), r681(710), r685(714), r688(717), r690(719), r692(721), r693(722), r697(726), r719(748), r721(750), r729(757), r730(758), r731(759), r740(767), r755(782), r792(816), r793(817), r808(832), r812(836), r813(837), r814(838), r816(840), r767(793), r820(843), r124(165), r256(292), r255(291)
	Depth zone	r333(364), r392(422), r394(424), r395(425), r396(426), r397(427), r398(428), r399(429), r400(430), r401(431), r402(432), r403(433), r404(434), r405(435), r406(436), r407(437), r409(439), r410(440), r411(441), r412(442), r413(443), r415(445), r416(446), r417(447), r418(448), r419(449), r797(824), r421(451), r422(452), r423(453), r424(454), r425(455), r426(456), r427(457), r428(458), r429(459), r430(460), r431(461), r432(462), r433(463), r434(464), r435(465), r436(466), r438(468), r440(470), r327(358), r441(471), r442(472), r443(473), r444(474), r445(475), r446(476), r447(477), r448(478), r450(479), r451(480), r452(481), r453(482), r454(483), r455(484), r456(485), r457(486), r458(487), r459(488), r460(489), r461(490), r462(491), r463(492), r464(493), r465(494), r466(495), r467(496), r468(497), r469(498), r470(499), r472(501), r473(502), r474(503), r475(504), r476(505), r478(507), r479(508), r480(509), r477(506), r482(511), r483(512), r484(513), r485(514), r486(515), r821(848), r487(516), r488(517), r489(518), r490(519), r491(520), r492(521), r493(522), r494(523), r495(524), r496(525), r497(526), r498(527), r499(528), r500(529), r501(530), r502(531), r503(532), r504(533), r505(534), r506(535), r507(536), r508(537), r509(538), r510(539), r511(540), r512(541), r513(542), r514(543), r790(817), r830(857), r831(858), r832(859), r515(544), r516(545), r517(546), r518(547), r519(548), r520(549), r521(550), r522(551),

		r523(552), r524(553), r525(554), r526(555), r527(556), r528(557), r529(558), r530(559), r531(560), r532(561), r533(562), r534(563), r535(564), r536(565), r537(566), r538(567), r539(568), r540(569), r541(570), r542(571), r543(572), r544(573), r545(574), r546(575), r547(576), r548(577), r549(578), r550(579), r551(580), r552(581), r553(582), r554(583), r555(584), r556(585), r557(586), r558(587), r559(588), r560(589), r561(590), r562(591), r563(592), r564(593), r565(594), r566(595), r567(596), r568(597), r569(598), r570(599), r571(600), r572(601), r573(602), r574(603), r575(604), r576(605), r577(606), r578(607), r579(608), r580(609), r581(610), r582(611), r583(612), r584(613), r585(614), r586(615), r587(616), r588(617), r589(618), r590(619), r591(620), r592(621), r593(622), r594(623), r595(624), r596(625), r597(626), r598(627), r599(628), r600(629), r601(630), r602(631), r603(632), r604(633), r605(634), r606(635), r607(636), r608(637), r609(638), r610(639), r611(640), r612(641), r613(642), r614(643), r615(644), r617(646), r618(647), r619(648), r620(649), r621(650), r622(651), r623(652), r625(654), r626(655), r627(656), r628(657), r629(658), r631(660), r632(661), r633(662), r634(663), r635(664), r636(665), r637(666), r638(667), r639(668), r640(669), r641(670), r642(671), r643(672), r644(673), r646(675), r647(676), r648(677), r649(678), r650(679), r651(680), r655(684), r656(685), r657(686), r658(687), r659(688), r660(689), r662(691), r663(692), r664(693), r665(694), r666(695), r667(696), r668(697), r669(698), r671(700), r672(701), r673(702), r674(703), r676(705), r677(706), r678(707), r681(710), r682(711), r683(712), r685(714), r686(715), r687(716), r688(717), r689(718), r690(719), r691(720), r692(721), r693(722), r695(724), r696(725), r697(726), r698(727), r709(738), r710(739), r719(748), r720(749), r721(750), r722(751), r725(754), r726(36), r727(755), r728(756), r729(757), r730(758), r731(759), r732(760), r733(761), r736(763), r739(766), r740(767), r741(768), r742(769), r744(771), r745(772), r747(774), r748(775), r749(776), r751(778), r755(782), r760(787), r767(793), r771(797), r772(798), r773(799), r775(800), r780(804), r789(813), r792(816), r793(817), r795(819), r805(829), r808(832), r809(833), r810(834), r812(836), r813(837), r814(838), r815(839), r816(840), r817(32), r820(843), r124(165), r823(846), r835(858), r806(830)
	Feeding mechanism	r553(582), r376(407)
	Food sources	r700(729), r701(730), r702(731), r703(732), r704(733), r705(734), r706(735), r707(736), r708(737), r710(739), r711(740), r712(741), r713(742), r714(743), r715(744), r716(745), r718(747), r723(752), r724(753), r750(777), r756(783), r758(785), r806(830), r811(835)
	Light zone	r333(364), r392(422), r394(424), r395(425), r396(426), r397(427), r398(428), r399(429), r400(430), r401(431), r402(432), r404(434), r405(435), r406(436), r407(437), r409(439), r410(440), r411(441), r412(442), r413(443), r415(445), r416(446), r417(447), r418(448), r419(449), r797(824), r422(452), r424(454), r425(455), r426(456), r427(457), r428(458), r429(459), r430(460), r431(461), r432(462), r433(463), r434(464), r435(465), r436(466), r438(468), r440(470), r327(358), r441(471), r442(472), r443(473), r444(474), r445(475), r446(476), r447(477), r448(478), r450(479), r451(480), r452(481), r453(482), r454(483), r455(484), r456(485), r457(486), r458(487), r459(488), r460(489), r461(490), r462(491), r463(492), r464(493), r465(494), r466(495), r467(496), r468(497), r469(498), r470(499), r472(501), r473(502), r474(503), r475(504), r476(505), r478(507), r479(508), r480(509), r477(506), r482(511), r483(512), r484(513), r485(514), r486(515), r821(848), r487(516), r488(517), r489(518), r490(519), r491(520), r492(521), r493(522), r494(523), r495(524), r496(525), r497(526), r498(527), r499(528), r500(529), r501(530), r502(531), r503(532), r504(533), r505(534), r506(535), r507(536), r508(537), r509(538), r510(539), r511(540), r512(541), r513(542), r514(543), r790(817), r830(857), r831(858), r832(859), r515(544), r516(545), r517(546), r518(547), r519(548), r520(549), r521(550), r522(551), r523(552), r524(553), r526(555), r527(556), r528(557), r529(558), r530(559), r531(560), r532(561), r533(562), r534(563), r535(564), r536(565), r537(566), r538(567), r539(568), r540(569), r541(570), r542(571), r543(572), r544(573), r546(575), r547(576), r548(577), r549(578), r550(579), r551(580), r552(581), r554(583), r556(585), r557(586), r558(587), r559(588), r560(589), r561(590), r562(591), r564(593), r565(594), r566(595), r567(596), r573(602), r574(603), r576(605), r577(606), r578(607), r579(608), r583(612), r584(613), r585(614), r586(615), r587(616), r588(617), r589(618), r591(620), r592(621), r593(622), r594(623), r597(626), r598(627), r599(628), r600(629), r602(631), r603(632), r604(633), r605(634), r606(635), r608(637), r612(641), r613(642), r619(648), r622(651), r623(652), r626(655), r628(657), r633(662), r636(665), r637(666), r643(672), r646(675), r647(676), r649(678), r650(679), r651(680), r655(684), r657(686), r659(688), r660(689), r663(692), r664(693), r665(694), r666(695), r667(696), r669(698), r672(701), r677(706), r678(707), r681(710), r682(711), r683(712), r685(714), r688(717), r689(718), r690(719), r691(720), r692(721), r693(722), r695(724), r696(725), r697(726), r698(727), r709(738), r710(739), r719(748), r720(749), r721(750), r722(751), r725(754), r726(36), r727(755), r728(756), r729(757), r730(758), r731(759), r732(760), r733(761), r736(763), r739(766), r740(767), r741(768), r742(769), r744(771), r745(772), r747(774), r748(775), r749(776), r751(778), r755(782), r760(787), r767(793), r771(797), r772(798), r773(799), r775(800), r780(804), r789(813), r792(816), r793(817), r805(829), r808(832), r809(833), r810(834), r812(836), r813(837), r814(838), r815(839), r816(840), r817(32), r820(843), r124(165), r823(846), r835(858), r806(830)
	Size of prey items	r701(730), r702(731), r704(733), r706(735), r708(737), r710(739), r712(741), r715(744), r717(746), r718(747), r806(830)
	Type locality depth	r375(406), r333(364), r378(409), r389(420), r392(422), r395(425), r396(426), r397(427), r409(439), r410(440), r411(441), r412(442), r413(443), r415(445), r416(446), r417(447), r418(448), r419(449), r797(824), r422(452), r423(453), r424(454), r425(455), r426(456), r427(457), r428(458), r430(460), r431(461), r432(462), r433(463), r434(464), r435(465), r438(468), r440(470), r441(471), r442(472), r443(473), r444(474), r445(475), r446(476), r447(477), r448(478), r451(480), r452(481), r453(482), r454(483), r455(484), r456(485), r457(486), r458(487), r459(488), r460(489), r463(492), r465(494), r466(495), r467(496), r468(497), r469(498), r450(479), r473(502), r474(503), r475(504), r478(507), r479(508), r480(509), r477(506), r482(511), r483(512), r484(513), r485(514), r486(515), r821(848), r487(516), r488(517), r489(518), r490(519), r491(520), r492(521), r493(522), r494(523), r496(525), r497(526), r498(527), r499(528), r501(530), r502(531), r504(533), r505(534), r506(535), r507(536), r508(537), r509(538), r510(539), r511(540), r512(541), r513(542), r514(543), r515(544), r516(545), r517(546), r519(548), r520(549), r521(550), r522(551), r523(552), r524(553), r526(555), r528(557), r529(558), r531(560), r532(561), r533(562), r534(563), r535(564), r536(565), r537(566), r538(567), r539(568), r540(569), r544(573),

		r545(574), r548(577), r549(578), r550(579), r551(580), r552(581), r555(584), r557(586), r558(587), r559(588), r560(589), r562(591), r563(592), r564(593), r565(594), r566(595), r567(596), r569(598), r570(599), r571(600), r572(601), r573(602), r574(603), r575(604), r576(605), r577(606), r578(607), r581(610), r582(611), r583(612), r584(613), r586(615), r587(616), r588(617), r589(618), r590(619), r591(620), r592(621), r593(622), r594(623), r595(624), r596(625), r597(626), r598(627), r600(629), r601(630), r602(631), r604(633), r606(635), r607(636), r608(637), r609(638), r611(640), r612(641), r613(642), r614(643), r615(644), r617(646), r618(647), r619(648), r620(649), r621(650), r622(651), r623(652), r625(654), r626(655), r628(657), r631(660), r633(662), r635(664), r636(665), r638(667), r640(669), r642(671), r643(672), r644(673), r646(675), r647(676), r648(677), r649(678), r650(679), r651(680), r658(687), r659(688), r660(689), r662(691), r663(692), r664(693), r665(694), r666(695), r667(696), r669(698), r671(700), r672(701), r673(702), r677(706), r678(707), r685(714), r690(719), r691(720), r692(721), r693(722), r695(724), r697(726), r698(727), r725(754), r742(769), r747(774), r749(776), r790(814), r808(832), r815(839), r816(840), r124(165), r835(858)
	Water clarity preference	r333(364), r389(420), r419(449)
	Wave exposure preference	r333(364), r397(427), r423(453), r820(843)
Geographical	Climate zone	r341(372), r340(371), r342(373), r333(364), r343(374), r344(375), r345(376), r346(377), r347(378), r348(379), r349(380), r350(381), r351(382), r352(383), r353(384), r354(385), r355(386), r356(387), r357(388), r358(389), r359(390), r360(391), r361(392), r362(393), r363(394), r364(395), r365(396), r366(397), r367(398), r368(399), r369(400), r370(401), r371(402), r372(403), r374(405), r378(409), r389(420), r392(422), r393(423), r394(424), r395(425), r396(426), r397(427), r398(428), r399(429), r400(430), r401(431), r402(432), r403(433), r404(434), r405(435), r406(436), r407(437), r408(438), r409(439), r410(440), r411(441), r412(442), r413(443), r414(444), r415(445), r416(446), r417(447), r418(448), r419(449), r797(821), r420(450), r421(451), r422(452), r423(453), r424(454), r427(457), r429(459), r430(460), r431(461), r432(462), r434(464), r435(465), r436(466), r438(468), r439(469), r440(470), r327(358), r441(471), r442(472), r443(473), r444(474), r445(475), r446(476), r447(477), r448(478), r450(479), r451(480), r452(481), r453(482), r454(483), r455(484), r456(485), r457(486), r458(487), r459(488), r460(489), r461(490), r462(491), r463(492), r464(493), r465(494), r466(495), r467(496), r468(497), r469(498), r470(499), r472(501), r473(502), r474(503), r475(504), r476(505), r478(507), r479(508), r480(509), r477(506), r481(510), r482(511), r483(512), r484(513), r485(514), r486(515), r821(844), r487(516), r488(517), r489(518), r490(519), r491(520), r492(521), r493(522), r494(523), r495(524), r496(525), r497(526), r498(527), r499(528), r500(529), r501(530), r502(531), r503(532), r504(533), r505(534), r506(535), r507(536), r508(537), r509(538), r510(539), r511(540), r512(541), r513(542), r514(543), r831(858), r832(859), r515(544), r516(545), r517(546), r518(547), r519(548), r520(549), r521(550), r522(551), r523(552), r524(553), r525(554), r526(555), r527(556), r528(557), r529(558), r530(559), r531(560), r532(561), r533(562), r534(563), r535(564), r536(565), r537(566), r538(567), r539(568), r540(569), r541(570), r542(571), r543(572), r544(573), r545(574), r546(575), r547(576), r548(577), r549(578), r550(579), r551(580), r552(581), r553(582), r554(583), r555(584), r556(585), r557(586), r558(587), r559(588), r560(589), r561(590), r562(591), r563(592), r564(593), r565(594), r566(595), r567(596), r568(597), r569(598), r570(599), r571(600), r572(601), r573(602), r574(603), r575(604), r576(605), r577(606), r578(607), r579(608), r580(609), r581(610), r582(611), r583(612), r584(613), r585(614), r586(615), r587(616), r588(617), r589(618), r590(619), r591(620), r592(621), r593(622), r594(623), r595(624), r596(625), r597(626), r598(627), r599(628), r600(629), r601(630), r602(631), r603(632), r604(633), r605(634), r606(635), r607(636), r608(637), r609(638), r610(639), r611(640), r612(641), r613(642), r614(643), r615(644), r616(645), r617(646), r618(647), r619(648), r620(649), r621(650), r622(651), r623(652), r625(654), r626(655), r627(656), r628(657), r629(658), r631(660), r632(661), r633(662), r634(663), r635(664), r636(665), r637(666), r638(667), r639(668), r640(669), r641(670), r642(671), r643(672), r644(673), r646(675), r647(676), r648(677), r649(678), r650(679), r651(680), r655(684), r656(685), r657(686), r658(687), r659(688), r660(689), r662(691), r663(692), r664(693), r665(694), r666(695), r667(696), r668(697), r669(698), r670(699), r671(700), r672(701), r673(702), r674(703), r676(705), r677(706), r678(707), r681(710), r682(711), r683(712), r685(714), r686(715), r687(716), r688(717), r689(718), r690(719), r691(720), r692(721), r693(722), r695(724), r696(725), r697(726), r698(727), r709(738), r710(739), r720(749), r721(750), r722(751), r725(754), r726(36), r727(755), r728(756), r729(757), r730(758), r731(759), r732(760), r733(761), r736(763), r739(766), r740(767), r741(768), r742(769), r744(771), r745(772), r747(774), r748(775), r749(776), r755(782), r760(787), r767(793), r771(797), r772(798), r773(799), r789(813), r790(814), r792(816), r793(817), r794(818), r795(819), r805(829), r808(832), r809(833), r812(836), r813(837), r814(838), r815(839), r816(840), r817(32), r819(842), r820(843), r124(165), r822(845), r823(846), r835(858)
	Marine province	r335(366), r336(367), r339(370), r343(374), r344(375), r348(379), r350(381), r351(382), r354(385), r356(387), r357(388), r358(389), r359(390), r360(391), r363(394), r364(395), r365(396), r366(397), r367(398), r368(399), r369(400), r370(401), r371(402), r374(405), r390(421), r333(364), r378(409), r389(420), r392(422), r393(423), r394(424), r395(425), r396(426), r397(427), r398(428), r399(429), r400(430), r401(431), r402(432), r403(433), r404(434), r405(435), r406(436), r407(437), r408(438), r409(439), r410(440), r411(441), r412(442), r413(443), r414(444), r415(445), r416(446), r417(447), r418(448), r419(449), r797(821), r420(450), r421(451), r422(452), r423(453), r424(454), r425(455), r426(456), r427(457), r428(458), r429(459), r430(460), r431(461), r432(462), r434(464), r435(465), r436(466), r438(468), r439(469), r440(470), r327(358), r441(471), r442(472), r443(473), r444(474), r445(475), r446(476), r447(477), r448(478), r450(479), r451(480), r452(481), r453(482), r454(483), r455(484), r456(485), r457(486), r458(487), r459(488), r460(489), r461(490), r462(491), r463(492), r464(493), r465(494), r466(495), r467(496), r468(497), r469(498), r470(499), r472(501), r473(502), r474(503), r475(504), r476(505), r478(507), r479(508), r480(509), r477(506), r481(510), r482(511), r483(512), r484(513), r485(514), r486(515), r821(848), r487(516), r488(517), r489(518), r490(519), r491(520), r492(521), r493(522), r494(523), r495(524), r496(525), r497(526), r498(527), r499(528), r500(529), r501(530), r502(531), r503(532), r504(533), r505(534), r506(535), r507(536), r508(537), r509(538), r510(539), r511(540), r512(541), r513(542), r514(543),

		r831(854), r832(855), r515(544), r516(545), r517(546), r518(547), r519(548), r520(549), r521(550), r522(551), r523(552), r524(553), r525(554), r526(555), r527(556), r528(557), r529(558), r530(559), r531(560), r532(561), r533(562), r534(563), r535(564), r536(565), r537(566), r538(567), r539(568), r540(569), r541(570), r542(571), r543(572), r544(573), r545(574), r546(575), r548(577), r549(578), r550(579), r551(580), r552(581), r553(582), r554(583), r555(584), r556(585), r557(586), r558(587), r559(588), r560(589), r561(590), r562(591), r563(592), r564(593), r565(594), r566(595), r567(596), r568(597), r569(598), r570(599), r571(600), r572(601), r573(602), r574(603), r575(604), r576(605), r577(606), r578(607), r579(608), r580(609), r581(610), r582(611), r583(612), r584(613), r585(614), r586(615), r587(616), r588(617), r589(618), r590(619), r591(620), r592(621), r593(622), r594(623), r595(624), r596(625), r597(626), r598(627), r599(628), r600(629), r601(630), r602(631), r603(632), r604(633), r605(634), r606(635), r607(636), r608(637), r609(638), r610(639), r611(640), r612(641), r613(642), r614(643), r615(644), r616(645), r617(646), r618(647), r619(648), r620(649), r621(650), r622(651), r623(652), r625(654), r626(655), r627(656), r628(657), r629(658), r631(660), r632(661), r633(662), r634(663), r635(664), r636(665), r637(666), r638(667), r639(668), r640(669), r641(670), r642(671), r643(672), r644(673), r646(675), r647(676), r648(677), r649(678), r650(679), r651(680), r655(684), r656(685), r657(686), r658(687), r659(688), r660(689), r662(691), r663(692), r664(693), r665(694), r666(695), r667(696), r668(697), r669(698), r670(699), r671(700), r672(701), r673(702), r674(703), r676(705), r677(706), r678(707), r681(710), r682(711), r683(712), r685(714), r686(715), r687(716), r688(717), r689(718), r690(719), r691(720), r692(721), r693(722), r695(724), r696(725), r697(726), r698(727), r709(738), r710(739), r720(749), r721(750), r722(751), r725(754), r726(36), r727(755), r728(756), r729(757), r730(758), r731(759), r732(760), r733(761), r736(763), r739(766), r740(767), r741(768), r742(769), r744(771), r745(772), r747(774), r748(775), r749(776), r755(782), r760(787), r767(793), r771(797), r772(798), r773(799), r789(813), r790(814), r792(816), r793(817), r794(818), r795(819), r805(829), r808(832), r809(833), r812(836), r813(837), r814(838), r815(839), r816(840), r817(32), r819(842), r820(843), r124(165), r822(845), r823(846), r835(858)
	Marine realm	r335(366), r333(364), r336(367), r337(368), r338(369), r339(370), r340(371), r341(372), r342(373), r343(374), r344(375), r348(379), r350(381), r351(382), r352(383), r353(384), r354(385), r356(387), r357(388), r358(389), r359(390), r360(391), r361(392), r362(393), r363(394), r364(395), r365(396), r366(397), r367(398), r368(399), r369(400), r370(401), r371(402), r372(403), r374(405), r390(421), r378(409), r389(420), r392(422), r393(423), r394(424), r395(425), r396(426), r397(427), r398(428), r399(429), r400(430), r401(431), r402(432), r403(433), r404(434), r405(435), r406(436), r407(437), r408(438), r409(439), r410(440), r411(441), r412(442), r413(443), r414(444), r415(445), r416(446), r417(447), r418(448), r419(449), r797(821), r420(450), r421(451), r422(452), r423(453), r424(454), r425(455), r426(456), r427(457), r428(458), r429(459), r430(460), r431(461), r432(462), r434(464), r435(465), r436(466), r438(468), r439(469), r440(470), r327(358), r441(471), r442(472), r443(473), r444(474), r445(475), r446(476), r447(477), r448(478), r450(479), r451(480), r452(481), r453(482), r454(483), r455(484), r456(485), r457(486), r458(487), r459(488), r460(489), r461(490), r462(491), r463(492), r464(493), r465(494), r466(495), r467(496), r468(497), r469(498), r470(499), r472(501), r473(502), r474(503), r475(504), r476(505), r478(507), r479(508), r480(509), r477(506), r481(510), r482(511), r483(512), r484(513), r485(514), r486(515), r821(848), r487(516), r488(517), r489(518), r490(519), r491(520), r492(521), r493(522), r494(523), r495(524), r496(525), r497(526), r498(527), r499(528), r500(529), r501(530), r502(531), r503(532), r504(533), r505(534), r506(535), r507(536), r508(537), r509(538), r510(539), r511(540), r512(541), r513(542), r514(543), r831(854), r832(855), r515(544), r516(545), r517(546), r518(547), r519(548), r520(549), r521(550), r522(551), r523(552), r524(553), r525(554), r526(555), r527(556), r528(557), r529(558), r530(559), r531(560), r532(561), r533(562), r534(563), r535(564), r536(565), r537(566), r538(567), r539(568), r540(569), r541(570), r542(571), r543(572), r544(573), r545(574), r546(575), r547(576), r548(577), r549(578), r550(579), r551(580), r552(581), r553(582), r554(583), r555(584), r556(585), r557(586), r558(587), r559(588), r560(589), r561(590), r562(591), r563(592), r564(593), r565(594), r566(595), r567(596), r568(597), r569(598), r570(599), r571(600), r572(601), r573(602), r574(603), r575(604), r576(605), r577(606), r578(607), r579(608), r580(609), r581(610), r582(611), r583(612), r584(613), r585(614), r586(615), r587(616), r588(617), r589(618), r590(619), r591(620), r592(621), r593(622), r594(623), r595(624), r596(625), r597(626), r598(627), r599(628), r600(629), r601(630), r602(631), r603(632), r604(633), r605(634), r606(635), r607(636), r608(637), r609(638), r610(639), r611(640), r612(641), r613(642), r614(643), r615(644), r616(645), r617(646), r618(647), r619(648), r620(649), r621(650), r622(651), r623(652), r625(654), r626(655), r627(656), r628(657), r629(658), r631(660), r632(661), r633(662), r634(663), r635(664), r636(665), r637(666), r638(667), r639(668), r640(669), r641(670), r642(671), r643(672), r644(673), r646(675), r647(676), r648(677), r649(678), r650(679), r651(680), r655(684), r656(685), r657(686), r658(687), r659(688), r660(689), r662(691), r663(692), r664(693), r665(694), r666(695), r667(696), r668(697), r669(698), r670(699), r671(700), r672(701), r673(702), r674(703), r676(705), r677(706), r678(707), r681(710), r682(711), r683(712), r685(714), r686(715), r687(716), r688(717), r689(718), r690(719), r691(720), r692(721), r693(722), r695(724), r696(725), r697(726), r698(727), r709(738), r710(739), r720(749), r721(750), r722(751), r725(754), r726(36), r727(755), r728(756), r729(757), r730(758), r731(759), r732(760), r733(761), r736(763), r739(766), r740(767), r741(768), r742(769), r744(771), r745(772), r747(774), r748(775), r749(776), r755(782), r760(787), r767(793), r771(797), r772(798), r773(799), r789(813), r790(814), r792(816), r793(817), r794(818), r795(819), r805(829), r808(832), r809(833), r812(836), r813(837), r814(838), r815(839), r816(840), r817(32), r819(842), r820(843), r124(165), r822(845), r823(846), r835(858)
	Ocean basin	r335(366), r333(364), r336(367), r337(368), r338(369), r339(370), r340(371), r341(372), r342(373), r343(374), r344(375), r345(376), r346(377), r347(378), r348(379), r349(380), r350(381), r351(382), r352(383), r353(384), r354(385), r355(386), r356(387), r357(388), r358(389), r359(390), r360(391), r361(392), r362(393), r363(394), r364(395), r365(396), r366(397), r367(398), r368(399), r369(400), r370(401), r371(402), r372(403), r374(405), r390(421), r378(409), r389(420), r392(422), r393(423), r394(424), r395(425), r396(426), r397(427), r398(428), r399(429), r400(430), r401(431), r402(432), r403(433), r404(434), r405(435), r406(436), r407(437), r408(438), r409(439), r410(440), r411(441), r417(447), r418(448), r434(464), r435(465), r436(466), r438(468), r439(469), r440(470), r327(358), r441(471), r442(472), r443(473), r444(474), r445(475), r446(476), r447(477), r448(478),

		r450(479), r451(480), r452(481), r453(482), r454(483), r455(484), r456(485), r457(486), r458(487), r459(488), r460(489), r461(490), r462(491), r463(492), r464(493), r465(494), r466(495), r467(496), r468(497), r469(498), r470(499), r472(501), r473(502), r474(503), r475(504), r476(505), r478(507), r479(508), r480(509), r477(506), r481(510), r482(511), r483(512), r484(513), r485(514), r486(515), r821(844), r487(516), r488(517), r489(518), r490(519), r491(520), r492(521), r493(522), r494(523), r495(524), r496(525), r497(526), r498(527), r499(528), r500(529), r501(530), r502(531), r503(532), r504(533), r505(534), r506(535), r507(536), r508(537), r509(538), r510(539), r511(540), r512(541), r513(542), r514(543), r790(817), r831(854), r832(859), r515(544), r516(545), r517(546), r518(547), r519(548), r520(549), r521(550), r522(551), r523(552), r524(553), r525(554), r526(555), r527(556), r528(557), r529(558), r530(559), r531(560), r532(561), r533(562), r534(563), r535(564), r536(565), r537(566), r538(567), r539(568), r540(569), r541(570), r542(571), r543(572), r544(573), r545(574), r546(575), r547(576), r548(577), r549(578), r550(579), r551(580), r552(581), r553(582), r554(583), r555(584), r556(585), r557(586), r558(587), r559(588), r560(589), r561(590), r562(591), r563(592), r564(593), r565(594), r566(595), r567(596), r568(597), r569(598), r570(599), r571(600), r572(601), r573(602), r574(603), r575(604), r576(605), r577(606), r578(607), r579(608), r580(609), r581(610), r582(611), r583(612), r584(613), r585(614), r586(615), r587(616), r588(617), r589(618), r590(619), r591(620), r592(621), r593(622), r594(623), r595(624), r596(625), r597(626), r598(627), r599(628), r600(629), r601(630), r602(631), r603(632), r604(633), r605(634), r606(635), r607(636), r608(637), r609(638), r610(639), r611(640), r612(641), r613(642), r614(643), r615(644), r616(645), r617(646), r618(647), r619(648), r620(649), r621(650), r622(651), r623(652), r625(654), r626(655), r627(656), r628(657), r629(658), r631(660), r632(661), r633(662), r634(663), r635(664), r636(665), r637(666), r638(667), r639(668), r640(669), r641(670), r642(671), r643(672), r644(673), r646(675), r647(676), r648(677), r649(678), r650(679), r651(680), r655(684), r656(685), r657(686), r658(687), r659(688), r660(689), r662(691), r663(692), r664(693), r665(694), r666(695), r667(696), r668(697), r669(698), r670(699), r671(700), r672(701), r673(702), r674(703), r676(705), r677(706), r678(707), r681(710), r682(711), r683(712), r685(714), r686(715), r687(716), r688(717), r689(718), r690(719), r691(720), r692(721), r693(722), r695(724), r696(725), r697(726), r698(727), r709(738), r710(739), r720(749), r721(750), r722(751), r725(754), r726(36), r727(755), r728(756), r729(757), r730(758), r731(759), r732(760), r733(761), r736(763), r739(766), r740(767), r741(768), r742(769), r744(771), r745(772), r747(774), r748(775), r749(776), r755(782), r760(787), r767(793), r771(797), r772(798), r773(799), r789(813), r792(816), r793(817), r794(818), r795(819), r797(821), r805(829), r808(832), r809(833), r812(836), r813(837), r814(838), r815(839), r816(840), r817(32), r819(842), r820(843), r124(165), r822(845), r823(846), r835(858)
Morphological	Branch diameter	r375(406), r642(671), r644(673), r647(676), r650(679), r656(685), r660(689), r663(692), r666(695), r672(701), r673(702), r679(708), r692(721), r693(722), r695(724), r743(770), r744(771), r749(7786, r790(814), r797(821), r805(829), r812(836), r815(839), r820(843), r124(165)
	Branching architecture	r311(342), r332(363), r335(366), r372(403), r336(367), r338(369), r339(370), r341(372), r342(373), r333(364), r352(383), r353(384), r354(385), r356(387), r357(388), r358(389), r359(390), r360(391), r361(392), r362(393), r363(394), r375(406), r392(422), r393(423), r394(424), r415(445), r797(824), r424(454), r425(455), r434(464), r327(358), r443(473), r444(474), r447(477), r448(478), r455(484), r456(485), r465(494), r470(499), r450(479), r451(480), r471(500), r467(496), r473(502), r475(504), r476(505), r478(507), r479(508), r480(509), r481(510), r483(512), r484(513), r485(514), r486(515), r821(844), r487(516), r488(517), r489(518), r490(519), r491(520), r492(521), r493(522), r494(523), r495(524), r496(525), r499(528), r500(529), r503(532), r504(533), r505(534), r506(535), r508(537), r512(541), r513(542), r514(543), r832(855), r516(545), r518(547), r520(549), r521(550), r522(551), r523(552), r526(555), r528(557), r529(558), r530(559), r531(560), r536(565), r545(574), r548(577), r550(579), r554(583), r558(587), r559(588), r562(591), r567(596), r569(598), r571(600), r572(601), r573(602), r575(604), r576(605), r577(606), r581(610), r582(611), r583(612), r586(615), r587(616), r588(617), r589(618), r591(620), r592(621), r594(623), r596(625), r597(626), r598(627), r600(629), r601(630), r603(632), r604(633), r608(637), r611(640), r612(641), r614(643), r619(648), r622(651), r623(652), r624(653), r625(654), r627(656), r628(657), r633(662), r634(663), r635(664), r636(665), r637(666), r638(667), r640(669), r641(670), r642(671), r643(672), r645(674), r647(676), r649(678), r650(679), r652(681), r655(684), r656(685), r660(689), r663(692), r664(693), r665(694), r666(695), r668(697), r669(698), r671(700), r672(701), r677(706), r678(707), r690(719), r692(721), r693(722), r695(724), r744(771), r749(7786), r753(780), r790(814), r795(819), r800(824), r809(833), r812(836), r816(840), r817(32), r818(841), r820(843), r124(165)
	Calyx height	r316(347), r319(350), r336(367), r341(372), r397(427), r409(439), r411(441), r413(443), r797(824), r423(453), r435(465), r444(474), r447(477), r448(478), r450(479), r452(481), r455(484), r463(492), r470(499), r485(514), r486(515), r821(844), r493(522), r494(523), r508(537), r509(538), r517(546), r524(553), r533(562), r536(565), r543(572), r545(574), r548(577), r557(586), r558(587), r559(588), r570(599), r571(600), r575(604), r577(606), r581(610), r583(612), r585(614), r588(617), r590(619), r593(622), r596(625), r608(637), r625(654), r633(662), r634(663), r635(664), r638(667), r640(669), r651(680), r659(688), r666(695), r672(701), r678(707), r692(721), r693(722), r698(727), r744(771), r770(797), r790(814), r815(839), r124(165)
	Calyx width	r316(347), r341(372), r435(465), r444(474), r447(477), r448(478), r450(479), r452(481), r455(484), r456(485), r463(492), r470(499), r485(514), r486(515), r821(844), r493(522), r494(523), r508(537), r509(538), r517(546), r524(553), r533(562), r536(565), r543(572), r548(577), r558(587), r559(588), r570(599), r572(601), r580(609), r583(612), r585(614), r588(617), r590(619), r593(622), r596(625), r599(628), r608(637), r633(662), r634(663), r635(664), r640(669), r642(671), r651(680), r659(688), r666(695), r676(705), r678(707), r679(708), r692(721), r693(722), r697(726), r727(755), r790(814), r815(839), r124(165)
	Coloniality	r377(408), r697(726), r817(32)
	Colony area	r108(153), r796(820)
	Colony height	r6(65), r7(66), r8(67), r12(70), r13(71), r16(74), r17(75), r21(79), r26(84), r28(86), r29(87), r37(95), r43(101), r45(103), r46(104), r55(111), r66(114), r20(78), r67(115), r35(93), r70(118), r73(121), r79(127), r80(128), r81(129), r82(130), r85(133), r86(134), r87(135), r90(138), r91(139), r95(142), r100(146), r99(145), r102(148),

		r103(149), r104(150), r105(151), r107(152), r114(155), r115(156), r116(157), r117(158), r118(159), r133(174), r137(178), r142(183), r138(179), r147(188), r148(189), r175(214), r187(225), r193(231), r216(253), r152(192), r218(255), r222(259), r224(261), r827(854), r235(272), r237(274), r239(276), r244(281), r247(284), r258(294), r264(299), r280(314), r292(326), r329(360), r331(362), r332(363), r335(366), r333(364), r336(367), r337(368), r338(369), r339(370), r341(372), r340(371), r342(373), r343(374), r344(375), r345(376), r346(377), r348(379), r349(380), r350(381), r351(382), r352(383), r353(384), r354(385), r355(386), r356(387), r357(388), r358(389), r359(390), r360(391), r361(392), r362(393), r363(394), r364(395), r365(396), r366(397), r367(398), r368(399), r369(400), r370(401), r371(402), r375(406), r380(411), r381(412), r382(413), r383(414), r384(415), r385(416), r386(417), r387(418), r388(419), r378(409), r389(420), r392(422), r393(423), r394(424), r395(425), r396(426), r397(427), r398(428), r399(429), r400(430), r401(431), r402(432), r403(433), r404(434), r405(435), r406(436), r407(437), r411(441), r412(442), r414(444), r416(446), r417(447), r418(448), r419(449), r797(824), r422(452), r423(453), r424(454), r425(455), r426(456), r427(457), r428(458), r430(460), r431(461), r433(463), r434(464), r438(468), r440(470), r441(471), r442(472), r444(474), r445(475), r446(476), r447(477), r448(478), r450(479), r451(480), r452(481), r453(482), r455(484), r456(485), r457(486), r458(487), r459(488), r460(489), r461(490), r463(492), r465(494), r466(495), r467(496), r468(497), r469(498), r470(499), r473(502), r829(856), r474(503), r475(504), r476(505), r478(507), r479(508), r477(506), r481(510), r482(511), r483(512), r485(514), r486(515), r821(844), r487(516), r488(517), r489(518), r490(519), r491(520), r492(521), r493(522), r494(523), r495(524), r496(525), r497(526), r498(527), r499(528), r500(529), r501(530), r502(531), r503(532), r504(533), r505(534), r506(535), r507(536), r508(537), r509(538), r510(539), r511(540), r512(541), r513(542), r514(543), r790(814), r830(853), r831(854), r832(855), r515(544), r516(545), r517(546), r518(547), r519(548), r520(549), r521(550), r522(551), r523(552), r524(553), r525(554), r526(555), r527(556), r528(557), r529(558), r531(560), r532(561), r533(562), r534(563), r535(564), r536(565), r537(566), r539(568), r540(569), r541(570), r542(571), r543(572), r544(573), r545(574), r546(575), r547(576), r548(577), r549(578), r550(579), r551(580), r552(581), r553(582), r554(583), r555(584), r557(586), r558(587), r559(588), r560(589), r562(591), r563(592), r564(593), r565(594), r566(595), r567(596), r568(597), r569(598), r570(599), r571(600), r572(601), r573(602), r575(604), r576(605), r577(606), r578(607), r579(608), r580(609), r581(610), r582(611), r583(612), r584(613), r585(614), r586(615), r587(616), r588(617), r589(618), r591(620), r592(621), r593(622), r594(623), r595(624), r596(625), r597(626), r598(627), r599(628), r600(629), r601(630), r603(632), r604(633), r606(635), r607(636), r608(637), r609(638), r611(640), r612(641), r613(642), r614(643), r615(644), r616(645), r617(646), r618(647), r619(648), r620(649), r621(650), r622(651), r623(652), r625(654), r626(655), r627(656), r628(657), r629(658), r631(660), r633(662), r634(663), r635(664), r636(665), r638(667), r640(669), r642(671), r643(672), r644(673), r646(675), r647(676), r648(677), r649(678), r650(679), r651(680), r656(685), r657(686), r658(687), r659(688), r660(689), r662(691), r663(692), r664(693), r665(694), r666(695), r669(698), r670(699), r671(700), r672(701), r673(702), r674(703), r675(704), r677(706), r678(707), r679(708), r682(711), r684(713), r685(714), r688(717), r690(719), r691(720), r692(721), r693(722), r695(724), r697(726), r698(727), r709(738), r713(742), r717(746), r721(750), r722(751), r725(754), r726(36), r729(757), r730(758), r731(759), r732(760), r733(761), r735(49), r738(765), r739(766), r740(767), r741(768), r742(769), r745(772), r749(776), r751(778), r752(779), r753(780), r754(781), r757(784), r760(787), r764(791), r765(792), r767(793), r768(794), r771(797), r776(801), r778(803), r780(804), r782(806), r789(813), r802(826), r803(827), r805(829), r806(830), r808(832), r810(834), r811(835), r813(837), r814(838), r815(839), r816(840), r818(841), r819(842), r820(843), r124(165), r14(72), r252(289), r835(858)
	Colony width	r6(65), r18(76), r80(128), r81(129), r118(159), r144(185), r138(179), r147(188), r153(193), r218(255), r264(299), r329(360), r335(366), r336(367), r338(369), r339(370), r341(372), r340(371), r345(376), r349(380), r352(383), r353(384), r362(393), r363(394), r375(406), r385(416), r386(417), r388(419), r333(364), r389(420), r392(422), r395(425), r396(426), r397(427), r398(428), r399(429), r400(430), r401(431), r402(432), r403(433), r404(434), r405(435), r406(436), r407(437), r411(441), r412(442), r414(444), r416(446), r417(447), r418(448), r419(449), r797(824), r422(452), r423(453), r424(454), r425(455), r426(456), r427(457), r428(458), r430(460), r431(461), r433(463), r434(464), r435(465), r438(468), r440(470), r441(471), r442(472), r445(475), r446(476), r447(477), r448(478), r451(480), r453(482), r455(484), r456(485), r457(486), r458(487), r459(488), r460(489), r461(490), r465(494), r466(495), r467(496), r468(497), r469(498), r470(499), r450(479), r473(502), r474(503), r475(504), r478(507), r482(511), r483(512), r485(514), r486(515), r821(844), r487(516), r489(518), r490(519), r491(520), r492(521), r493(522), r494(523), r496(525), r497(526), r498(527), r499(528), r500(529), r501(530), r502(531), r503(532), r504(533), r506(535), r507(536), r508(537), r510(539), r511(540), r512(541), r514(543), r515(544), r516(545), r517(546), r518(547), r519(548), r520(549), r521(550), r522(551), r524(553), r526(555), r527(556), r528(557), r529(558), r532(561), r533(562), r535(564), r536(565), r537(566), r540(569), r541(570), r543(572), r544(573), r545(574), r547(576), r548(577), r549(578), r550(579), r552(581), r557(586), r558(587), r559(588), r560(589), r563(592), r564(593), r565(594), r567(596), r568(597), r569(598), r571(600), r572(601), r575(604), r576(605), r577(606), r578(607), r579(608), r581(610), r582(611), r585(614), r586(615), r588(617), r589(618), r592(621), r593(622), r594(623), r595(624), r596(625), r597(626), r598(627), r599(628), r600(629), r601(630), r603(632), r604(633), r606(635), r607(636), r609(638), r610(639), r611(640), r612(641), r614(643), r615(644), r617(646), r618(647), r619(648), r620(649), r623(652), r625(654), r626(655), r627(656), r628(657), r631(660), r633(662), r634(663), r635(664), r636(665), r640(669), r642(671), r643(672), r646(675), r649(678), r650(679), r658(687), r660(689), r663(692), r665(694), r666(695), r670(699), r671(700), r672(701), r674(703), r677(706), r678(707), r684(713), r685(714), r688(717), r690(719), r692(721), r693(722), r697(726), r698(727), r725(754), r727(755), r731(759), r732(760), r739(766), r740(767), r741(768), r742(769), r790(814), r802(826), r806(830), r808(832), r815(839), r816(840), r818(841), r819(842), r820(843), r124(165)
	Coordination of polyps	r645(674), r664(693), r665(694), r666(695), r669(698), r670(699), r671(700), r672(701), r673(702), r677(706), r678(707), r691(720), r692(721), r693(722), r695(724), r697(726), r725(754), r742(769), r790(814), r797(821), r800(824), r809(833), r816(840), r124(165)
	Diameter of axis skeleton	r18(76), r45(103), r524(553), r576(605), r597(626), r623(652), r649(678), r665(694)

	Distance between polyps	r650(679), r663(692), r664(693), r667(696), r669(698), r670(699), r673(702), r677(706), r678(707), r691(720), r695(724), r697(726), r698(727), r742(769), r743(770), r762(789), r790(814), r800(824), r805(829), r809(833), r815(839), r124(165)
	Fractal dimension	r819(842)
	Growth form	r122(163), r143(184), r145(186), r147(188), r135(176), r192(230), r216(253), r257(293), r258(294), r263(298), r292(326), r295(328), r297(330), r309(340), r311(342), r314(345), r316(347), r331(362), r332(363), r333(364), r335(366), r372(403), r336(367), r337(368), r338(369), r339(370), r340(371), r341(372), r342(373), r343(374), r344(375), r345(376), r346(377), r348(379), r349(380), r350(381), r352(383), r353(384), r354(385), r355(386), r356(387), r357(388), r358(389), r359(390), r360(391), r361(392), r362(393), r363(394), r364(395), r365(396), r366(397), r367(398), r368(399), r369(400), r370(401), r371(402), r374(405), r375(406), r380(411), r381(412), r382(413), r383(414), r384(415), r385(416), r378(409), r389(420), r392(422), r393(423), r394(424), r395(425), r396(426), r397(427), r398(428), r399(429), r400(430), r401(431), r402(432), r403(433), r404(434), r405(435), r406(436), r407(437), r409(439), r410(440), r411(441), r412(442), r413(443), r414(444), r415(445), r416(446), r417(447), r418(448), r419(449), r797(821), r422(452), r423(453), r424(454), r425(455), r426(456), r427(457), r428(458), r430(460), r431(461), r433(463), r434(464), r435(465), r436(466), r438(468), r439(469), r440(470), r441(471), r442(472), r443(473), r444(474), r445(475), r446(476), r447(477), r448(478), r450(479), r451(480), r452(481), r453(482), r454(483), r455(484), r456(485), r457(486), r458(487), r459(488), r460(489), r461(490), r463(492), r465(494), r466(495), r468(497), r469(498), r470(499), r471(500), r467(496), r473(502), r474(503), r475(504), r476(505), r477(506), r478(507), r479(508), r480(509), r481(510), r482(511), r483(512), r484(513), r485(514), r486(515), r821(844), r487(516), r488(517), r489(518), r490(519), r491(520), r492(521), r493(522), r494(523), r495(524), r496(525), r497(526), r498(527), r500(529), r499(528), r501(530), r502(531), r503(532), r504(533), r505(534), r506(535), r507(536), r508(537), r509(538), r510(539), r511(540), r512(541), r513(542), r514(543), r790(814), r830(853), r831(854), r832(855), r515(544), r516(545), r517(546), r518(547), r519(548), r520(549), r521(550), r522(551), r523(552), r524(553), r525(554), r526(555), r527(556), r528(557), r529(558), r530(559), r531(560), r532(561), r533(562), r534(563), r536(565), r537(566), r538(567), r539(568), r540(569), r541(570), r542(571), r543(572), r544(573), r545(574), r546(575), r547(576), r548(577), r549(578), r550(579), r551(580), r552(581), r554(583), r555(584), r556(585), r557(586), r558(587), r559(588), r560(589), r561(590), r562(591), r563(592), r564(593), r565(594), r566(595), r567(596), r568(597), r569(598), r570(599), r571(600), r572(601), r573(602), r574(603), r575(604), r576(605), r577(606), r579(608), r580(609), r581(610), r582(611), r583(612), r584(613), r585(614), r586(615), r587(616), r588(617), r589(618), r590(619), r591(620), r592(621), r593(622), r594(623), r595(624), r596(625), r597(626), r598(627), r599(628), r600(629), r601(630), r603(632), r604(633), r606(635), r607(636), r608(637), r609(638), r610(639), r611(640), r612(641), r613(642), r614(643), r615(644), r616(645), r617(646), r618(647), r619(648), r620(649), r621(650), r622(651), r623(652), r624(653), r625(654), r626(655), r627(656), r628(657), r632(661), r633(662), r634(663), r635(664), r636(665), r637(666), r638(667), r640(669), r641(670), r642(671), r643(672), r644(673), r645(674), r646(675), r647(676), r648(677), r649(678), r650(679), r651(680), r652(681), r654(683), r655(684), r656(685), r657(686), r658(687), r659(688), r660(689), r661(690), r662(691), r663(692), r664(693), r665(694), r666(695), r667(696), r668(697), r669(698), r670(699), r671(700), r672(701), r673(702), r674(703), r676(705), r677(706), r678(707), r685(714), r690(719), r691(720), r692(721), r693(722), r695(724), r697(726), r698(727), r709(738), r721(750), r722(751), r725(754), r730(758), r731(759), r732(760), r733(761), r735(49), r736(763), r739(766), r740(767), r742(769), r744(771), r745(772), r747(774), r749(776), r751(778), r752(779), r753(780), r755(782), r767(793), r774(94), r786(810), r788(812), r792(816), r795(819), r800(824), r803(827), r805(829), r806(830), r808(832), r810(834), r812(836), r813(837), r814(838), r815(839), r816(840), r817(32), r818(841), r819(842), r820(843), r124(165), r256(292), r215(252), r255(291), r252(289), r254(290), r251(288), r835(858)
	Growth form (broad) (Gómez-Gras and Fabricius)	r122(163), r143(184), r145(186), r147(188), r135(176), r192(230), r216(253), r257(293), r258(294), r263(298), r292(326), r295(328), r297(330), r309(340), r311(342), r314(345), r316(347), r331(362), r332(363), r333(364), r335(366), r372(403), r336(367), r337(368), r338(369), r339(370), r340(371), r341(372), r342(373), r343(374), r344(375), r345(376), r346(377), r348(379), r349(380), r350(381), r352(383), r353(384), r354(385), r355(386), r356(387), r357(388), r358(389), r359(390), r360(391), r361(392), r362(393), r363(394), r364(395), r365(396), r366(397), r367(398), r368(399), r369(400), r370(401), r371(402), r374(405), r375(406), r380(411), r381(412), r382(413), r383(414), r384(415), r385(416), r378(409), r389(420), r392(422), r393(423), r394(424), r395(425), r396(426), r397(427), r398(428), r399(429), r400(430), r401(431), r402(432), r403(433), r404(434), r405(435), r406(436), r409(439), r410(440), r411(441), r412(442), r413(443), r414(444), r415(445), r416(446), r417(447), r418(448), r419(449), r797(821), r422(452), r423(453), r424(454), r425(455), r426(456), r427(457), r428(458), r431(461), r432(462), r433(463), r434(464), r435(465), r436(466), r438(468), r439(469), r440(470), r327(358), r441(471), r442(472), r443(473), r444(474), r445(475), r446(476), r447(477), r448(478), r451(480), r452(481), r453(482), r454(483), r455(484), r456(485), r457(486), r458(487), r459(488), r460(489), r461(490), r462(491), r463(492), r465(494), r466(495), r468(497), r469(498), r470(499), r450(479), r471(500), r467(496), r473(502), r474(503), r475(504), r476(505), r477(506), r478(507), r479(508), r480(509), r481(510), r482(511), r483(512), r484(513), r485(514), r486(515), r821(844), r487(516), r488(517), r489(518), r490(519), r491(520), r492(521), r493(522), r494(523), r495(524), r497(526), r498(527), r500(529), r499(528), r502(531), r503(532), r504(533), r505(534), r506(535), r507(536), r508(537), r509(538), r510(539), r511(540), r512(541), r513(542), r514(543), r790(814), r830(853), r831(854), r832(855), r515(544), r516(545), r517(546), r518(547), r519(548), r520(549), r521(550), r522(551), r523(552), r524(553), r525(554), r526(555), r527(556), r528(557), r529(558), r530(559), r531(560), r532(561), r533(562), r534(563), r535(564), r536(565), r537(566), r538(567), r539(568), r540(569), r541(570), r542(571), r543(572), r544(573), r545(574), r546(575), r547(576), r548(577), r549(578), r550(579), r551(580), r552(581), r553(582), r554(583), r555(584), r556(585), r557(586), r558(587), r559(588), r560(589), r561(590), r562(591), r563(592), r564(593), r565(594), r566(595), r567(596), r568(597), r569(598), r570(599), r571(600), r572(601), r573(602), r574(603), r575(604), r576(605), r577(606), r578(607), r579(608), r580(609), r581(610), r582(611), r583(612), r584(613), r585(614), r586(615), r587(616), r588(617), r589(618), r590(619),

		r591(620), r592(621), r593(622), r594(623), r595(624), r596(625), r597(626), r598(627), r599(628), r600(629), r601(630), r603(632), r604(633), r606(635), r607(636), r608(637), r609(638), r610(639), r611(640), r612(641), r613(642), r614(643), r615(644), r616(645), r617(646), r618(647), r619(648), r620(649), r621(650), r622(651), r623(652), r624(653), r625(654), r626(655), r627(656), r628(657), r631(660), r632(661), r633(662), r634(663), r635(664), r636(665), r637(666), r640(669), r641(670), r642(671), r643(672), r644(673), r645(674), r646(675), r647(676), r648(677), r649(678), r650(679), r651(680), r652(681), r654(683), r655(684), r656(685), r657(686), r658(687), r659(688), r660(689), r661(690), r662(691), r663(692), r664(693), r665(694), r666(695), r667(696), r668(697), r669(698), r670(699), r671(700), r672(701), r673(702), r674(703), r676(705), r677(706), r678(707), r685(714), r686(715), r690(719), r691(720), r692(721), r693(722), r695(724), r697(726), r698(727), r709(738), r721(750), r722(751), r725(754), r729(757), r730(758), r731(759), r732(760), r733(761), r735(49), r736(763), r739(766), r740(767), r742(769), r744(771), r745(772), r747(774), r749(776), r751(778), r752(779), r753(780), r755(782), r767(793), r774(94), r786(810), r788(812), r792(816), r795(819), r800(824), r803(827), r805(829), r806(830), r808(832), r809(833), r810(834), r812(836), r813(837), r814(838), r815(839), r816(840), r817(32), r818(841), r819(842), r820(843), r124(165), r256(292), r215(252), r255(291), r252(289), r254(290), r251(288), r835(858)
	Growth form (detailed) (Gómez-Gras & Fabricius)	r122(163), r143(184), r145(186), r147(188), r135(176), r192(230), r216(253), r257(293), r258(294), r292(326), r295(328), r297(330), r309(340), r311(342), r314(345), r316(347), r331(362), r332(363), r333(364), r335(366), r372(403), r336(367), r337(368), r338(369), r339(370), r340(371), r341(372), r342(373), r343(374), r344(375), r345(376), r346(377), r348(379), r349(380), r350(381), r352(383), r353(384), r354(385), r355(386), r356(387), r357(388), r358(389), r359(390), r360(391), r361(392), r362(393), r363(394), r364(395), r365(396), r366(397), r367(398), r368(399), r369(400), r370(401), r371(402), r374(405), r375(406), r380(411), r381(412), r382(413), r383(414), r384(415), r385(416), r378(409), r389(420), r392(422), r393(423), r394(424), r395(425), r396(426), r397(427), r398(428), r399(429), r400(430), r401(431), r402(432), r403(433), r404(434), r405(435), r406(436), r409(439), r410(440), r411(441), r412(442), r413(443), r414(444), r415(445), r416(446), r417(447), r418(448), r419(449), r797(821), r422(452), r423(453), r424(454), r425(455), r426(456), r427(457), r428(458), r431(461), r432(462), r433(463), r434(464), r435(465), r436(466), r438(468), r439(469), r440(470), r327(358), r441(471), r442(472), r443(473), r444(474), r445(475), r446(476), r447(477), r448(478), r451(480), r452(481), r453(482), r454(483), r455(484), r456(485), r457(486), r458(487), r459(488), r460(489), r461(490), r462(491), r463(492), r465(494), r466(495), r468(497), r469(498), r470(499), r450(479), r471(500), r467(496), r473(502), r474(503), r475(504), r476(505), r477(506), r478(507), r479(508), r480(509), r481(510), r482(511), r483(512), r484(513), r485(514), r486(515), r821(844), r487(516), r488(517), r489(518), r490(519), r491(520), r492(521), r493(522), r494(523), r495(524), r497(526), r498(527), r500(529), r499(528), r502(531), r503(532), r504(533), r505(534), r506(535), r507(536), r508(537), r509(538), r510(539), r511(540), r512(541), r513(542), r514(543), r790(817), r830(853), r831(854), r832(855), r515(544), r516(545), r517(546), r518(547), r519(548), r520(549), r521(550), r522(551), r523(552), r524(553), r525(554), r526(555), r527(556), r528(557), r529(558), r530(559), r531(560), r532(561), r533(562), r534(563), r535(564), r536(565), r537(566), r538(567), r539(568), r540(569), r541(570), r542(571), r543(572), r544(573), r545(574), r546(575), r547(576), r548(577), r549(578), r550(579), r551(580), r552(581), r553(582), r554(583), r555(584), r556(585), r557(586), r558(587), r559(588), r560(589), r561(590), r562(591), r563(592), r564(593), r565(594), r566(595), r567(596), r568(597), r569(598), r570(599), r571(600), r572(601), r573(602), r574(603), r575(604), r576(605), r577(606), r578(607), r579(608), r580(609), r581(610), r582(611), r583(612), r584(613), r585(614), r586(615), r587(616), r588(617), r589(618), r590(619), r591(620), r592(621), r593(622), r594(623), r595(624), r596(625), r597(626), r598(627), r599(628), r600(629), r601(630), r603(632), r604(633), r606(635), r607(636), r608(637), r609(638), r610(639), r611(640), r612(641), r613(642), r614(643), r615(644), r616(645), r617(646), r618(647), r619(648), r620(649), r621(650), r622(651), r623(652), r624(653), r625(654), r626(655), r627(656), r628(657), r631(660), r632(661), r633(662), r634(663), r635(664), r636(665), r637(666), r640(669), r641(670), r642(671), r643(672), r644(673), r645(674), r646(675), r647(676), r648(677), r649(678), r650(679), r651(680), r652(681), r654(683), r655(684), r656(685), r657(686), r658(687), r659(688), r660(689), r661(690), r662(691), r663(692), r664(693), r665(694), r666(695), r667(696), r668(697), r669(698), r670(699), r671(700), r672(701), r673(702), r674(703), r676(705), r677(706), r678(707), r685(714), r686(715), r690(719), r691(720), r692(721), r693(722), r695(724), r697(726), r698(727), r709(738), r721(750), r722(751), r725(754), r729(757), r730(758), r731(759), r732(760), r733(761), r735(49), r736(763), r739(766), r740(769), r742(771), r744(773), r745(774), r747(776), r749(778), r751(780), r752(781), r753(782), r755(784), r767(793), r774(94), r786(810), r788(812), r792(816), r795(819), r800(824), r803(827), r805(829), r806(830), r808(832), r809(833), r810(834), r812(836), r813(837), r814(838), r815(839), r816(840), r817(32), r818(841), r819(842), r820(843), r124(165), r256(292), r215(252), r255(291), r252(289), r254(290), r251(288), r835(858)
	Number of pinnules per tentacle	r74(122), r136(177), r147(188), r175(214), r343(374), r344(375), r348(379), r351(382), r369(400), r370(401), r371(402), r702(731)
	Number of tentacles per polyp	r376(407), r817(32)
	Peduncle length	r646(675), r651(680), r691(720), r811(835)
	Polyp density	r21(79), r627(656), r628(657), r633(662), r638(667), r649(678), r665(694), r672(701), r673(702), r677(706), r693(722), r695(724), r713(742), r717(746), r747(774), r762(789), r790(814), r791(815), r800(824), r811(835), r816(840)
	Polyp diameter	r40(98), r74(122), r132(173), r136(177), r158(198), r170(209), r175(214), r275(310), r292(326), r309(340), r314(345), r336(367), r337(368), r339(370), r340(371), r341(372), r342(373), r343(374), r344(375), r354(385), r369(400), r375(406), r333(364), r389(420), r397(427), r401(431), r402(432), r409(439), r411(441), r412(442), r416(446), r434(464), r435(465), r443(473), r444(474), r457(486), r465(494), r466(495), r467(496), r451(480), r478(507), r479(508), r480(509), r481(510), r483(512), r484(513), r485(514), r486(515), r821(844), r487(516),

		r490(519), r491(520), r492(521), r496(525), r497(526), r498(527), r500(529), r502(531), r503(532), r504(533), r506(535), r510(539), r511(540), r512(541), r513(542), r514(543), r515(544), r516(545), r519(548), r520(549), r521(550), r522(551), r524(553), r526(555), r527(556), r534(563), r535(564), r537(566), r538(567), r542(571), r544(573), r547(576), r551(580), r560(589), r562(591), r563(592), r564(593), r565(594), r566(595), r567(596), r568(597), r569(598), r570(599), r576(605), r578(607), r579(608), r584(613), r585(614), r586(615), r587(616), r591(620), r593(622), r594(623), r597(626), r603(632), r604(633), r607(636), r609(638), r612(641), r613(642), r614(643), r616(645), r617(646), r618(647), r619(648), r623(652), r626(655), r628(657), r636(665), r643(672), r646(675), r647(676), r648(677), r649(678), r650(679), r651(680), r658(687), r660(689), r663(692), r664(693), r665(694), r666(695), r667(696), r669(698), r670(699), r671(700), r672(701), r674(703), r685(714), r690(719), r692(721), r697(726), r698(727), r702(731), r713(742), r717(746), r727(755), r742(769), r743(770), r747(774), r762(789), r790(814), r805(829), r808(832), r809(833), r811(835), r814(838), r815(839), r816(840), r124(165), r255(291), r140(181), r425(455)
	Polyp dimorphism	r340(371), r341(372), r342(373), r333(364), r343(374), r354(385), r364(395), r366(397), r367(398), r368(399), r439(469), r442(472), r446(476), r460(489), r461(490), r464(493), r468(497), r450(479), r451(480), r473(502), r474(503), r482(511), r483(512), r497(526), r498(527), r502(531), r507(536), r511(540), r512(541), r513(542), r519(548), r527(556), r534(563), r539(568), r540(569), r541(570), r543(572), r547(576), r549(578), r551(580), r552(581), r555(584), r564(593), r566(595), r568(597), r578(607), r579(608), r584(613), r593(622), r594(623), r595(624), r610(639), r612(641), r613(642), r617(646), r618(647), r620(649), r621(650), r631(660), r632(661), r646(675), r648(677), r651(680), r657(686), r658(687), r659(688), r660(689), r662(691), r670(699), r685(714), r691(720), r692(721), r693(722), r697(726), r731(759), r736(763), r740(767), r815(839), r817(32), r124(165)
	Polyp height	r132(173), r136(177), r147(188), r158(198), r170(209), r175(214), r216(253), r291(325), r309(340), r331(362), r336(367), r337(368), r338(369), r339(370), r340(371), r341(372), r342(373), r345(376), r348(379), r349(380), r351(382), r356(387), r357(388), r360(391), r363(394), r364(395), r367(398), r369(400), r370(401), r371(402), r379(410), r380(411), r378(409), r389(420), r397(427), r401(431), r402(432), r411(441), r416(446), r425(455), r430(460), r434(464), r435(465), r436(466), r442(472), r444(474), r457(486), r461(490), r465(494), r466(495), r467(496), r468(497), r469(498), r451(480), r473(502), r476(505), r478(507), r479(508), r480(509), r477(506), r481(510), r483(512), r485(514), r486(515), r821(844), r487(516), r488(517), r489(518), r490(519), r491(520), r492(521), r496(525), r497(526), r498(527), r502(531), r503(532), r504(533), r505(534), r506(535), r510(539), r511(540), r512(541), r513(542), r514(543), r790(817), r830(853), r831(854), r832(855), r515(544), r516(545), r518(547), r519(548), r520(549), r522(551), r523(552), r524(553), r526(555), r530(559), r531(560), r534(563), r535(564), r537(566), r538(567), r539(568), r542(571), r544(573), r547(576), r549(578), r550(579), r551(580), r560(589), r562(591), r563(592), r564(593), r565(594), r566(595), r567(596), r568(597), r573(602), r576(605), r578(607), r584(613), r586(615), r587(616), r590(619), r591(620), r593(622), r594(623), r597(626), r598(627), r600(629), r602(631), r603(632), r604(633), r606(635), r607(636), r612(641), r615(644), r617(646), r618(647), r619(648), r621(650), r622(651), r623(652), r626(655), r628(657), r631(660), r636(665), r637(666), r643(672), r646(675), r647(676), r648(677), r649(678), r650(679), r651(680), r658(687), r660(689), r662(691), r663(692), r664(693), r665(694), r666(695), r667(696), r669(698), r670(699), r671(700), r672(701), r673(702), r674(703), r677(706), r685(714), r690(719), r691(720), r692(721), r695(724), r697(726), r698(727), r709(738), r713(742), r742(769), r747(774), r762(789), r767(793), r800(824), r805(829), r808(832), r809(833), r812(836), r813(837), r814(838), r815(839), r816(840), r124(165)
	Polyp retractability	r333(364), r397(427), r401(431), r409(439), r410(440), r411(441), r412(442), r413(443), r424(454), r430(460), r434(464), r435(465), r438(468), r439(469), r440(470), r442(472), r444(474), r446(476), r447(477), r448(478), r450(479), r460(489), r461(490), r463(492), r464(493), r468(497), r469(498), r470(499), r451(480), r471(500), r467(496), r473(502), r474(503), r475(504), r476(505), r477(506), r478(507), r479(508), r480(509), r482(511), r483(512), r484(513), r485(514), r486(515), r487(516), r497(526), r498(527), r499(528), r500(529), r501(530), r502(531), r508(537), r509(538), r511(540), r517(546), r527(556), r528(557), r529(558), r533(562), r538(567), r539(568), r540(569), r541(570), r542(571), r543(572), r544(573), r547(576), r549(578), r552(581), r555(584), r557(586), r558(587), r562(591), r563(592), r564(593), r565(594), r566(595), r568(597), r569(598), r570(599), r571(600), r572(601), r575(604), r579(608), r582(611), r583(612), r584(613), r585(614), r590(619), r591(620), r593(622), r594(623), r598(627), r600(629), r603(632), r607(636), r610(639), r612(641), r614(643), r615(644), r616(645), r617(646), r618(647), r620(649), r621(650), r624(653), r625(654), r627(656), r628(657), r632(661), r633(662), r642(671), r646(675), r648(677), r651(680), r656(685), r657(686), r658(687), r659(688), r660(689), r662(691), r664(693), r666(695), r667(696), r668(697), r670(699), r671(700), r685(714), r691(720), r692(721), r693(722), r695(724), r697(726), r731(759), r742(769), r747(774), r790(814), r792(816), r797(821), r800(824), r812(836), r813(837), r814(838), r815(839), r817(32), r124(165), r835(858)
	Polyp whorls/pairs per length unit	r665(694), r672(701), r677(706), r816(840)
	Polyps per polyp leaf	r646(675), r651(680)
	Polyps per whorl	r637(666), r665(694), r666(695), r671(700), r672(701), r677(706), r678(707), r692(721), r725(754), r816(840)
	Polyp leaves presence	r653(682), r657(686)
	Projected side area	r29(87), r84(132), r118(159), r732(760), r735(49), r741(768), r774(94), r819(843)
	Rachis length	r646(675), r651(680), r691(720), r717(746), r731(759), r752(779)
	Symmetry of rachis	r657(686), r691(720), r731(759)

	Tentacle length	r74(122), r132(173), r136(177), r147(188), r175(214), r316(347), r336(367), r337(368), r342(373), r369(400), r375(406), r378(409), r397(427), r402(432), r425(455), r461(490), r468(497), r497(526), r498(527), r502(531), r504(533), r513(542), r516(545), r519(548), r535(564), r538(567), r547(576), r551(580), r565(594), r566(595), r568(597), r578(607), r584(613), r607(636), r618(647), r621(650), r648(677), r658(687), r659(688), r663(692), r669(698), r670(699), r690(719), r691(720), r697(726), r702(731), r717(746), r813(837), r814(838), r815(839)
	Trunk diameter	r21(79), r29(87), r20(78), r66(114), r67(115), r68(116), r33(91), r35(93), r70(118), r75(123), r77(125), r82(130), r91(139), r94(141), r95(142), r99(145), r117(158), r175(214), r216(253), r244(281), r280(314), r332(363), r335(366), r336(367), r338(369), r339(370), r341(372), r342(373), r368(399), r386(417), r333(364), r378(409), r410(440), r412(442), r797(824), r423(453), r425(455), r434(464), r442(472), r444(474), r447(477), r448(478), r450(479), r451(480), r452(481), r453(482), r455(484), r460(489), r465(494), r467(496), r470(499), r475(504), r476(505), r479(508), r481(510), r482(511), r485(514), r486(515), r821(848), r487(516), r488(517), r489(518), r490(519), r491(520), r492(521), r493(522), r494(523), r495(524), r500(529), r503(532), r504(533), r505(534), r506(535), r510(539), r512(541), r513(542), r514(543), r515(544), r516(545), r517(546), r518(547), r519(548), r520(549), r521(550), r522(551), r528(557), r529(558), r531(560), r533(562), r536(565), r537(566), r543(572), r544(573), r545(574), r547(576), r548(577), r551(580), r552(581), r555(584), r558(587), r559(588), r560(589), r562(591), r564(593), r566(595), r567(596), r569(598), r571(600), r572(601), r573(602), r575(604), r576(605), r577(606), r579(608), r580(609), r581(610), r582(611), r583(612), r584(613), r586(615), r587(616), r588(617), r589(618), r593(622), r594(623), r595(624), r598(627), r599(628), r600(629), r601(630), r603(632), r604(633), r606(635), r608(637), r611(640), r612(641), r614(643), r615(644), r616(645), r617(646), r618(647), r619(648), r621(650), r625(654), r626(655), r628(657), r633(662), r634(663), r635(664), r636(665), r640(669), r642(671), r643(672), r649(678), r650(679), r651(680), r657(686), r660(689), r663(692), r664(693), r665(694), r666(695), r669(698), r670(699), r671(700), r672(701), r673(702), r677(706), r690(719), r692(721), r693(722), r695(724), r725(754), r757(784), r760(787), r790(814), r805(829), r808(832), r815(839), r816(840), r819(842), r124(165)
	Type of growth (Gómez-Gras & Fabricius)	r122(163), r143(184), r145(186), r147(188), r135(176), r192(230), r216(253), r257(293), r258(294), r263(298), r292(326), r295(328), r297(330), r309(340), r311(342), r314(345), r316(347), r331(362), r332(363), r333(364), r335(366), r372(403), r336(367), r337(368), r338(369), r339(370), r340(371), r341(372), r342(373), r343(374), r344(375), r345(376), r346(377), r348(379), r349(380), r350(381), r352(383), r353(384), r354(385), r355(386), r356(387), r357(388), r358(389), r359(390), r360(391), r361(392), r362(393), r363(394), r364(395), r365(396), r366(397), r367(398), r368(399), r369(400), r370(401), r371(402), r374(405), r375(406), r380(411), r381(412), r382(413), r383(414), r384(415), r385(416), r378(409), r389(420), r392(422), r393(423), r394(424), r395(425), r396(426), r397(427), r398(428), r399(429), r400(430), r401(431), r402(432), r403(433), r404(434), r405(435), r406(436), r409(439), r410(440), r411(441), r412(442), r413(443), r414(444), r415(445), r416(446), r417(447), r418(448), r419(449), r797(821), r422(452), r423(453), r424(454), r425(455), r426(456), r427(457), r428(458), r431(461), r432(462), r433(463), r434(464), r435(465), r436(466), r438(468), r439(469), r440(470), r327(358), r441(471), r442(472), r443(473), r444(474), r445(475), r446(476), r447(477), r448(478), r451(480), r452(481), r453(482), r454(483), r455(484), r456(485), r457(486), r458(487), r459(488), r460(489), r461(490), r462(491), r463(492), r465(494), r466(495), r468(497), r469(498), r470(499), r450(479), r471(500), r467(496), r473(502), r474(503), r475(504), r476(505), r477(506), r478(507), r479(508), r480(509), r481(510), r482(511), r483(512), r484(513), r485(514), r486(515), r821(844), r487(516), r488(517), r489(518), r490(519), r491(520), r492(521), r493(522), r494(523), r495(524), r497(526), r498(527), r500(529), r499(528), r502(531), r503(532), r504(533), r505(534), r506(535), r507(536), r508(537), r509(538), r510(539), r511(540), r512(541), r513(542), r514(543), r790(814), r830(853), r831(854), r832(855), r515(544), r516(545), r517(546), r518(547), r519(548), r520(549), r521(550), r522(551), r523(552), r524(553), r525(554), r526(555), r527(556), r528(557), r529(558), r530(559), r531(560), r532(561), r533(562), r534(563), r535(564), r536(565), r537(566), r538(567), r539(568), r540(569), r541(570), r542(571), r543(572), r544(573), r545(574), r546(575), r547(576), r548(577), r549(578), r550(579), r551(580), r552(581), r553(582), r554(583), r555(584), r556(585), r557(586), r558(587), r559(588), r560(589), r561(590), r562(591), r563(592), r564(593), r565(594), r566(595), r567(596), r568(597), r569(598), r570(599), r571(600), r572(601), r573(602), r574(603), r575(604), r576(605), r577(606), r578(607), r579(608), r580(609), r581(610), r582(611), r583(612), r584(613), r585(614), r586(615), r587(616), r588(617), r589(618), r590(619), r591(620), r592(621), r593(622), r594(623), r595(624), r596(625), r597(626), r598(627), r599(628), r600(629), r601(630), r603(632), r604(633), r606(635), r607(636), r608(637), r609(638), r610(639), r611(640), r612(641), r613(642), r614(643), r615(644), r616(645), r617(646), r618(647), r619(648), r620(649), r621(650), r622(651), r623(652), r624(653), r625(654), r626(655), r627(656), r628(657), r631(660), r632(661), r633(662), r634(663), r635(664), r636(665), r637(666), r640(669), r641(670), r642(671), r643(672), r644(673), r645(674), r646(675), r647(676), r648(677), r649(678), r650(679), r651(680), r652(681), r654(683), r655(684), r656(685), r657(686), r658(687), r659(688), r660(689), r661(690), r662(691), r663(692), r664(693), r665(694), r666(695), r667(696), r668(697), r669(698), r670(699), r671(700), r672(701), r673(702), r674(703), r676(705), r677(706), r678(707), r685(714), r686(715), r690(719), r691(720), r692(721), r693(722), r695(724), r697(726), r698(727), r709(738), r721(750), r722(751), r725(754), r729(757), r730(758), r731(759), r732(760), r733(761), r735(49), r736(763), r739(766), r740(767), r742(769), r744(771), r745(772), r747(774), r749(776), r751(778), r752(779), r753(780), r755(782), r767(793), r774(94), r786(810), r788(812), r792(816), r795(819), r800(824), r803(827), r805(829), r806(830), r808(832), r809(833), r810(834), r812(836), r813(837), r814(838), r815(839), r816(840), r817(32), r818(841), r819(842), r820(843), r124(165), r256(292), r215(252), r255(291), r252(289), r254(290), r251(288), r835(858)
	Polyp whorl diameter	r637(666), r677(706), r816(840)
Physiol ogical	Axis radial growth rate	r15(73), r16(74), r17(75), r18(76), r22(80), r23(81), r31(89), r32(90), r34(92), r35(93), r48(106), r50(108), r63(113), r66(114), r20(78), r67(115), r33(91), r69(117), r70(118), r71(119), r72(120), r73(121), r75(123), r94(141), r95(142), r99(145), r107(152), r119(160), r264(299), r778(803), r799(823), r14(72)
	Calcification rate	r88(136), r699(728), r772(798), r784(808), r787(811)

	Capture rate	r701(730), r706(735), r708(737), r711(740), r712(741), r713(742), r715(744), r716(745), r718(747), r756(783)
	Colony age	r6(65), r15(73), r16(74), r17(75), r18(76), r21(79), r22(80), r23(81), r26(84), r27(85), r29(87), r30(88), r37(95), r45(103), r48(106), r50(108), r60(112), r66(114), r20(78), r67(115), r33(91), r75(123), r77(125), r81(129), r82(130), r84(132), r91(139), r94(141), r95(142), r99(145), r103(149), r105(151), r107(152), r264(299), r345(376), r721(750), r799(823), r801(825)
	Dark respiration	r714(743)
	Gross photosynthesis	r714(743)
	Growth rate	r5(64), r6(65), r7(66), r8(67), r13(71), r10(68), r11(69), r12(70), r16(74), r17(75), r18(76), r21(79), r23(81), r24(82), r38(96), r25(83), r26(84), r27(85), r28(86), r29(87), r31(89), r35(93), r36(94), r37(95), r828(855), r40(98), r41(99), r42(100), r43(101), r44(102), r45(103), r47(105), r48(106), r49(107), r55(111), r60(112), r70(118), r73(121), r82(130), r84(132), r87(135), r88(136), r89(137), r90(138), r91(139), r93(140), r95(142), r99(145), r101(147), r102(148), r103(149), r104(150), r105(151), r108(153), r113(154), r118(159), r134(175), r133(174), r137(178), r149(190), r158(198), r167(206), r171(210), r192(230), r196(234), r209(246), r246(283), r264(299), r630(659), r680(709), r699(728), r722(751), r729(757), r768(794), r774(94), r794(818), r796(820), r819(842), r14(72), r834(857)
	Longevity	r24(82), r45(103), r227(264), r77(125), r345(376), r356(387), r737(764)
	Photosynthesis/respiration ratio (P/R)	r319(350), r714(743)
	Symbiodinium clade	r120(161), r296(329), r297(330), r299(332), r300(333), r301(334), r302(335), r303(336), r304(337), r306(339), r309(340), r310(341), r313(344), r314(345), r315(346), r317(348), r320(351), r321(352), r322(353), r323(354), r324(355), r325(356), r326(357), r532(561)
	Symbiont density	r714(743), r762(789), r763(790)
	Tissue thickness	r85(133), r175(214), r316(347)
	Zooxanthellate	r74(122), r83(131), r96(143), r120(161), r121(162), r122(163), r132(173), r133(174), r136(177), r140(181), r78(126), r138(179), r145(186), r146(187), r147(188), r128(169), r150(191), r137(178), r154(194), r156(196), r135(176), r164(203), r824(851), r165(204), r166(205), r243(280), r167(206), r825(852), r168(207), r173(212), r176(215), r174(213), r177(216), r236(273), r187(225), r188(226), r68(116), r192(230), r193(231), r90(138), r194(232), r199(237), r127(168), r206(243), r210(247), r216(253), r218(255), r141(182), r226(263), r227(264), r229(266), r233(270), r245(282), r263(298), r296(329), r297(330), r298(331), r299(332), r300(333), r301(334), r302(335), r304(337), r305(338), r306(339), r309(340), r310(341), r311(342), r312(343), r313(344), r314(345), r315(346), r316(347), r317(348), r318(349), r319(350), r320(351), r321(352), r322(353), r323(354), r324(355), r325(356), r326(357), r327(358), r328(359), r330(361), r333(364), r372(403), r472(501), r348(379), r346(377), r345(376), r344(375), r343(374), r349(380), r350(381), r351(382), r352(383), r353(384), r354(385), r355(386), r356(387), r357(388), r358(389), r359(390), r360(391), r361(392), r362(393), r363(394), r364(395), r365(396), r366(397), r367(398), r368(399), r369(400), r370(401), r371(402), r380(411), r381(412), r382(413), r383(414), r384(415), r385(416), r386(417), r387(418), r388(419), r378(409), r389(420), r412(442), r434(464), r435(465), r439(469), r444(474), r446(476), r459(488), r460(489), r461(490), r463(492), r464(493), r468(497), r469(498), r471(500), r473(502), r532(561), r538(567), r555(584), r568(597), r570(599), r578(607), r620(649), r658(687), r662(691), r693(722), r697(726), r714(743), r718(747), r727(755), r763(790), r812(836), r813(837), r817(32), r124(165), r214(251), r215(252), r125(166), r41(99), r213(250)
Reproductive	Age at maturity	r45(103), r60(112), r68(116), r70(118), r93(140), r101(147), r133(174), r138(179), r149(190), r153(193), r165(204), r167(206), r179(218), r185(223), r191(229), r204(241), r201(239), r183(222), r211(248), r152(192), r218(255), r233(270), r234(271), r244(281), r246(283), r258(294), r263(298), r292(326), r775(800), r212(249), r252(289)
	Asexual reproduction mode	r146(187), r265(300), r267(302), r271(306), r291(325), r249(286), r104(150), r294(327), r183(222), r344(375), r333(364), r407(437), r460(489), r733(761), r746(773), r796(820), r801(825), r820(843), r254(290)
	Colony fecundity	r67(115), r68(116), r227(264), r239(276), r242(279), r292(326), r252(289)
	Eggs per area	r150(191), r225(262), r218(255), r238(275), r268(303), r285(319)
	Frequency of reproduction	r143(184), r144(185), r138(179), r147(188), r149(190), r150(191), r94(141), r165(204), r168(207), r180(219), r181(220), r182(221), r185(223), r60(112), r68(116), r192(230), r194(232), r127(168), r199(237), r201(239), r211(248), r216(253), r223(260), r224(261), r227(264), r228(265), r229(266), r237(274), r240(277), r242(279), r243(280), r245(282), r190(228), r257(293), r259(295), r263(298), r277(312), r284(318), r292(326), r254(290), r252(289), r256(292), r250(287)
	Larval length	r24(82), r90(138), r132(173), r143(184), r153(193), r135(176), r165(204), r166(205), r169(208), r171(210), r176(215), r178(217), r180(219), r182(221), r41(99), r186(224), r187(225), r192(230), r200(238), r201(239), r202(240), r205(242), r131(172), r210(247), r231(268), r245(282), r244(281), r261(297), r272(307), r274(309), r285(319), r287(321), r512(541), r603(632), r216(253), r212(249)
	Larval swimming speed	r24(82), r270(305), r286(320)
	Larval width	r24(82), r90(138), r153(193), r165(204), r166(205), r180(219), r41(99), r186(224), r187(225), r201(239), r202(240), r210(247), r274(309), r512(541)
	Mode of larval development	r24(82), r40(98), r68(116), r77(125), r88(136), r93(140), r94(141), r125(166), r128(169), r126(167), r127(168), r129(170), r130(171), r131(172), r132(173), r136(177), r137(178), r143(184), r138(179), r145(186), r146(187), r147(188), r149(190), r150(191), r153(193), r155(195), r156(196), r157(197), r158(198), r160(200), r135(176), r161(201), r162(202), r824(851), r164(203), r165(204), r166(205), r243(280), r167(206), r825(852), r168(207), r169(208), r172(211), r173(212), r174(213), r176(215), r139(180), r178(217), r180(219), r181(220), r182(221),

		r41(99), r186(224), r187(225), r188(226), r189(227), r192(230), r193(231), r90(138), r194(232), r195(233), r196(234), r201(239), r202(240), r210(247), r211(248), r212(249), r216(253), r218(255), r219(256), r221(258), r223(260), r224(261), r225(262), r226(263), r227(264), r228(265), r229(266), r230(267), r232(269), r233(270), r238(275), r239(276), r240(277), r241(278), r242(279), r245(282), r244(281), r250(287), r257(293), r259(295), r263(298), r269(304), r273(308), r274(309), r277(312), r278(313), r281(315), r282(316), r283(317), r285(319), r286(320), r287(321), r290(324), r292(326), r416(446), r479(508), r491(520), r512(541), r603(632), r622(651), r650(679), r672(701), r769(795), r775(800), r255(291), r215(252), r213(250), r251(288), r252(289), r254(290), r256(292), r133(174)
	Oocyte size at maturity	r60(112), r68(116), r93(140), r94(141), r132(173), r133(174), r137(178), r141(182), r143(184), r144(185), r138(179), r146(187), r147(188), r149(190), r150(191), r153(193), r154(194), r155(195), r158(198), r159(199), r160(200), r135(176), r161(201), r826(853), r162(202), r157(197), r164(203), r165(204), r166(205), r243(280), r167(206), r825(852), r168(207), r172(211), r156(196), r175(214), r176(215), r178(217), r179(218), r181(220), r182(221), r185(223), r186(224), r187(225), r188(226), r189(227), r192(230), r193(231), r194(232), r195(233), r196(234), r198(236), r200(238), r201(239), r202(240), r205(242), r199(237), r206(243), r207(244), r127(168), r131(172), r183(222), r209(246), r210(247), r211(248), r212(249), r216(253), r152(192), r218(255), r219(256), r220(257), r221(258), r222(259), r223(260), r224(261), r225(262), r226(263), r227(264), r228(265), r229(266), r232(269), r233(270), r237(274), r238(275), r239(276), r240(277), r242(279), r245(282), r244(281), r190(228), r246(283), r248(285), r250(287), r257(293), r258(294), r259(295), r260(296), r261(297), r263(298), r270(305), r272(307), r273(308), r276(311), r277(312), r278(313), r280(314), r282(316), r284(318), r286(320), r288(322), r290(324), r291(325), r292(326), r638(667), r695(724), r742(769), r775(800), r791(815), r215(252), r254(290), r255(291), r213(250), r214(251), r251(288), r252(289), r256(292)
	Oogenic cycle duration	r133(174), r143(184), r144(185), r138(179), r146(187), r149(190), r153(193), r155(195), r158(198), r159(199), r160(200), r135(176), r161(201), r157(197), r165(204), r166(205), r825(852), r168(207), r176(215), r179(218), r139(180), r185(223), r186(224), r187(225), r188(226), r190(228), r192(230), r90(138), r194(232), r196(234), r200(238), r127(168), r202(240), r183(222), r211(248), r216(253), r218(255), r224(261), r225(262), r229(266), r232(269), r238(275), r242(279), r243(280), r245(282), r257(293), r263(298), r273(308), r280(314), r284(318), r290(324), r292(326), r775(800), r214(251), r215(252), r254(290), r212(249)
	Planktonic phase duration in larvae	r93(140), r94(141), r153(193), r165(204), r166(205), r131(172), r219(256), r227(264)
	Polyp fecundity	r67(115), r68(116), r90(138), r94(141), r133(174), r143(184), r144(185), r150(191), r153(193), r128(169), r155(195), r156(196), r159(199), r160(200), r135(176), r157(197), r162(202), r165(204), r166(205), r168(207), r174(213), r176(215), r180(219), r185(223), r186(224), r187(225), r188(226), r190(228), r193(231), r196(234), r198(236), r200(238), r127(168), r201(239), r202(240), r204(241), r199(237), r183(222), r209(246), r211(248), r216(253), r152(192), r220(257), r223(260), r224(261), r229(266), r230(267), r232(269), r233(270), r237(274), r240(277), r242(279), r245(282), r244(281), r246(283), r248(285), r257(293), r258(294), r259(295), r260(296), r263(298), r272(307), r273(308), r276(311), r277(312), r278(313), r280(314), r282(316), r284(318), r285(319), r288(322), r289(323), r290(324), r291(325), r292(326), r217(254), r603(632), r622(651), r638(667), r742(769), r791(815), r215(252), r255(291), r41(99), r214(251), r252(289), r256(292), r250(287)
	Propagule size on release	r94(141), r133(174), r137(178), r138(179), r147(188), r165(204), r166(205), r178(217), r182(221), r187(225), r192(230), r196(234), r131(172), r227(264), r285(319), r286(320)
	Reproductive period	r132(173), r133(174), r136(177), r137(178), r143(184), r144(185), r138(179), r146(187), r128(169), r147(188), r148(189), r149(190), r150(191), r153(193), r154(194), r155(195), r158(198), r159(199), r160(200), r135(176), r161(201), r162(202), r164(203), r165(204), r166(205), r243(280), r167(206), r825(852), r168(207), r170(209), r176(215), r178(217), r139(180), r179(218), r181(220), r182(221), r185(223), r186(224), r187(225), r188(226), r60(112), r68(116), r190(228), r192(230), r193(231), r90(138), r194(232), r195(233), r129(170), r196(234), r198(236), r200(238), r127(168), r201(239), r202(240), r199(237), r207(244), r208(245), r93(140), r211(248), r216(253), r218(255), r219(256), r221(258), r224(261), r225(262), r227(264), r229(266), r230(267), r238(275), r241(278), r242(279), r245(282), r244(281), r246(283), r248(285), r250(287), r257(293), r263(298), r272(307), r273(308), r277(312), r280(314), r281(315), r282(316), r283(317), r284(318), r285(319), r287(321), r288(322), r290(324), r291(325), r292(326), r775(800), r214(251), r254(290), r255(291), r41(99), r212(249), r251(288), r252(289), r256(292)
	Sexual system	r60(112), r67(115), r68(116), r77(125), r93(140), r94(141), r132(173), r133(174), r137(178), r140(181), r141(182), r143(184), r144(185), r138(179), r145(186), r147(188), r128(169), r149(190), r150(191), r153(193), r154(194), r155(195), r156(196), r157(197), r159(199), r160(200), r135(176), r161(201), r162(202), r164(203), r165(204), r166(205), r243(280), r167(206), r825(852), r168(207), r170(209), r172(211), r174(213), r176(215), r139(180), r178(217), r179(218), r181(220), r182(221), r185(223), r186(224), r187(225), r188(226), r189(227), r190(228), r192(230), r193(231), r194(232), r195(233), r41(99), r127(168), r198(236), r200(238), r201(239), r130(171), r202(240), r206(243), r207(244), r131(172), r208(245), r183(222), r210(247), r211(248), r212(249), r216(253), r152(192), r218(255), r219(256), r221(258), r222(259), r223(260), r224(261), r225(262), r226(263), r227(264), r228(265), r229(266), r232(269), r233(270), r237(274), r238(275), r239(276), r240(277), r242(279), r245(282), r244(281), r246(283), r248(285), r257(293), r258(294), r259(295), r263(298), r273(308), r277(312), r278(313), r280(314), r290(324), r292(326), r345(376), r346(377), r370(401), r371(402), r501(530), r517(546), r564(593), r585(614), r775(800), r215(252), r255(291), r251(288), r125(166), r213(250), r214(251), r252(289), r254(290), r256(292)
	Size at maturity	r40(98), r60(112), r93(140), r101(147), r133(174), r138(179), r149(190), r153(193), r167(206), r179(218), r186(224), r187(225), r190(228), r191(229), r194(232), r197(235), r204(241), r201(239), r183(222), r209(246), r211(248), r152(192), r218(255), r224(261), r228(265), r232(269), r233(270), r234(271), r237(274), r239(276), r242(279), r244(281), r246(283), r248(285), r258(294), r259(295), r263(298), r272(307), r273(308), r287(321), r289(323), r292(326), r775(803), r254(290), r212(249), r251(288), r252(289)

	Spawning date	r24(82), r136(177), r153(193), r135(176), r196(234), r198(236), r202(240), r205(242), r210(247), r238(275), r266(301), r270(305), r281(315), r288(322)
	Spermatogenic cycle duration	r143(184), r144(185), r149(190), r153(193), r155(195), r158(198), r159(199), r135(176), r161(201), r166(205), r186(224), r187(225), r190(228), r194(232), r200(238), r202(240), r211(248), r232(269), r238(275), r242(279), r243(280), r245(282), r257(293), r273(308), r284(318), r290(324), r292(326), r254(290), r212(249), r256(292), r250(287)
Stoichiometric	Total biomass	r28(86), r709(738), r752(779), r754(781), r761(788), r801(825)

Table S4. Hierarchical structure of the morphological classification system proposed for octocorals. There are seven simple Types of Growth (Trait 1) based on patterns of space occupation and represented in the table with different colors. These are subdivided into some further basic (Trait 2) and detailed (Trait 3) growth forms for finer scoring, as indicated by numbers and color tone gradients. Overall, this classification system aims to simplify the use of octocoral morphologies in ecological studies by classifying hundreds of similar growth forms used in the scientific literature, into a relatively manageable group of morphologies that have ecological meaning. For a detailed description of each trait category, see Tables S5-S11.

TRAIT 1: Type of growth	TRAIT 2: Growth form (basic)	TRAIT 3: Growth form (detailed)
**1.1 to 1.3 ERECT-BRANCHED**		
	1.1 Arborescent	
		1.1.1. Branched planar (simple)
		1.1.2. Branched planar (multi-fan)
		1.1.3. Bushy/Tree-like (TYPE: rigid/semirigid)
		1.1.4. Bushy/Tree-like (TYPE: soft)
		1.1.5. Bottlebrush
		1.1.6. Sparsely branched
		1.1.7. Arborescent with terminal branching
		1.1.8. Arborescent lobate
		1.1.9. Arborescent with domed capitula
	1.2 Erect branched (tangled)	
		1.2.1. Erect tangled (stoloniferous)
		1.2.2. Erect tangled (with axial skeletal support)
	1.3. Feather-like*	
		1.3.1. Feather-like
**2.1 to 2.2 ERECT-UNBRANCHED**		
	2.1 Erect unbranched (simple)	
		2.1.1. Flagelliform/Filiform
		2.1.2. Erect unbranched (pen-like)
		2.1.3. Digitiform
	2.2 Erect unbranched (capitate)	
		2.2.1. Erect unbranched with domed capitulum
		2.2.2. . Erect unbranched with disc-shaped capitulum
		2.2.3. Erect unbranched with umbellate cluster of polyps
**3. MASSIVE**		
	3.1. Massive	
		3.1.1. Massive
**4.1 to 4.6 SUB-MASSIVE**		
	4.1. Sub-Massive (lobate/digitate)	
		4.1.1. Sub-massive (lobate/digitate)
	4.2. Sub-Massive (columnar)	
		4.2.1. Sub-massive (columnar)
	4.3. Sub-Massive (branched)	
		4.3.1. Sub-massive (branched)
	4.4. Sub-Massive (foliose)	
		4.4.1. Sub-massive (foliose)
	4.5. Sub-Massive (with domed capitulum)	
		4.5.1. Sub-massive (with domed capitulum)
	4.6. Sub-Massive (plate-like)	
		4.6.1. Sub-massive (plate-like)
**5.1 to 5.2 HORIZONTAL UNBRANCHED**		
	5.1 Membranous (sheet-like)	
		5.1.1. Membranous (sheet-like)
	5.2. Encrusting	
		5.2.1. Encrusting (simple)
		5.2.2. Encrusting (digitate/lobate/folded)
		5.2.3. Encrusting (with verrucae)
**6.1. HORIZONTAL BRANCHED**		
	6.1 Encrusting (stoloniferous)	
		6.1.1. Encrusting (stoloniferous)
	6.2 Horizontal branched with axial skeletal support	
		6.2.1. Horizontal branched with axial skeletal support
**7.1 SOLITARY / PSEUDO-SOLITARY**		
	7.1. Solitary / Pseudo-solitary	
		7.1. Solitary / Pseudo-solitary

Table S5. Definition of each trait category for the Growth Form (basic) and Growth Form (detailed) traits, within the Erect Branched Type of Growth. An illustrative example of each detailed growth form has been included as well. References are shown under the table.

**1.1 to 1.3 ERECT BRANCHED**<br>Erect colonies having branches or branches-like structures (i.e., polyp leaves in sea pens), and with or without a stem/stalk. Colonies presenting this type of growth present a high vertical profile and have relatively small attachment areas in comparison with their main body, which is mostly in the water column.	
**1.1 Arborescent**<br>Erect branched colonies with a definite stem/stalk (Bayer, 1983). They can be rigid/semirigid (e.g., gorgonians supported by an internal skeletal axis) or flabby (e.g., soft corals with hydroskeleton). Moreover, branching can vary from very sparse to profuse, and occur from single to multiple planes.	
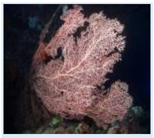	**1.1.1. Branched planar**: Arborescent colonies in which the branches grow mostly or totally in one plane (2D). Typical examples of this growth form are sea fans supported by a central skeleton (i.e., gorgonians forming anastomosed or irregular fans, lyrate/candelabriform forms...etc). However, in cases such as the family Siphonogorgiidae, large planar colonies can be supported by dense layers of surface sclerites that confer rigidity.<br>(Photo by D. Gómez-Gras; *Annella sp.*)
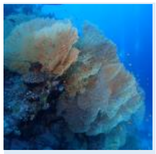	**1.1.2. Branched planar (multi-fan):** Arborescent colonies presenting multiple fans that can co-occur across different planes (3D).<br>(Photo by D. Gómez-Gras; *Annella mollis*)
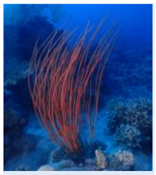	**1.1.3a. Bushy/Tree-like (TYPE rigid/semirigid):** Arborescent, firm colonies (e.g., with axial skeletal support) that present abundant branches arising in multiple directions (3D), resembling a bush or a tree. Strictly speaking, bushes would only refer to colonies with branches emerging immediately above the colony base and not forming an obvious main stem, whereas trees would require an obvious dominant stem (e.g., Bayer, 1983). Nevertheless, given the morpho-functional similarities of these two growth forms, we have grouped them together for this classification. Examples of this group include many gorgonian species such as *Plexaurella nutans or Ellisella ceratophyta.*<br>(Photo by D. Gómez-Gras; *Ellisella ceratophyta*)
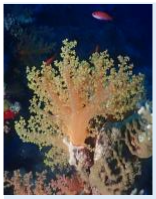	**1.1.3b. Bushy/Tree-like (TYPE soft):** Arborescent, flabby colonies (e.g., with hydroskeleton) with abundant branches arising in multiple directions (3D) resembling a bush or a tree. Strictly speaking, bushes would only refer to colonies with branches emerging immediately above the colony base not forming an obvious main stem, whereas trees would require an obvious dominant stem (e.g., Bayer, 1983). Nevertheless, given the morpho-functional similarities of these two growth forms, we have grouped them together for this classification. Typical examples of this group include soft coral species of the family Nephtheidae. *Bushy/tree-like soft corals with totally retractable branches (e.g., the genus *Ceeceenus*) are also included in this group.<br>(Photo by D. Gómez-Gras; *Dendronephthya sp.)*
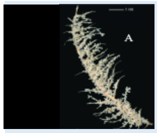	**1.1.4. Bottlebrush:** Arborescent colonies with numerous, crowded, short branchlets arising all around the main stem resembling a bottlebrush (e.g., some *Thouarella* species)<br>(Image from Guardiola-Zapata & López-González. 2010; *Thouarella undulata*)
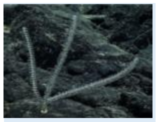	**1.1.5. Sparsely branched:** Arborescent colonies with very few branches (e.g., *Olindagorgia gracilis, Narella macrocalyx…*)<br>(Image source: Okeanos explorer; D2-EX1504-L4-11-21:25:50; NOAA; *Narella macrocalyx*)
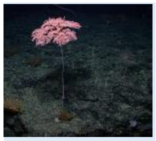	**1.1.6. Arborescent with terminal branching:** Arborescent (tree-like) colonies with an obvious dominant stem and a canopy of branches that only occurs at the distal, upper part of the colony (e.g., adult colonies of *Metallogorgia melanotrichos, Iridogorgia).* The main stem may be coiled such as the case of the genus *Iridigorgia*.<br>(Image source: NOAA; *Metallogorgia melanotrichos)*
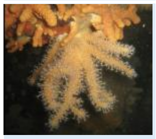	**1.1.7. Arborescent lobate:** Arborescent 3D colonies with lobate/digitate ramifications emerging from a single or bramble-like stalk. Most octocorals with this growth forms are soft (e.g., *Alcyonium palmatum*, *Sclerophytum polydactylum*…), but there are exceptions such as the case of *Nephthyigorgia*, where the lobes are brittle and rigid due to a dense layer of surface sclerites.<br>(Photo by: Matthieu Sontag; CC BY 3.0, *Alcyonium glomeratum*)

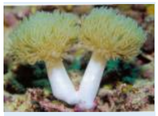

1.1.8. Arborescent with domed capitula: Arborescent 3D colonies with ramified stalks terminating in dome—shaped capitula bearing polyps (e.g., some Xeniids)

(Photo by: Roger Steene, in Fabricius and Alderslade, 2001; *Xenia sp.*)

1.2 Erect branched (tangled)

Erect branched colonies without a main stem or stalk, but with branches emerging in irregular or tangled patterns from the substrate

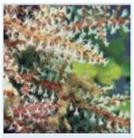

1.2.1. Erect tangled (stoloniferous): Erect colonies with primary and subordinate polyps emerging from branching stolons, leading in some cases to tangled, bushy appearances. A typical example is *C. riseii.*

(Photo by: K. Fabricius, in Fabricius and Alderslade, 2001; *Carijoa riseii*)

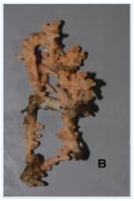

1.2.2. Erect tangled (with unconsolidated axis): Erect colonies in which a tangled mass of branches with unconsolidated axis emerges from membranes across the substratum (e.g., *Anthothela grandiflora*)

(Photo source: Moore et al. 2017, *Anthothela grandiflora*)

1.3 Feather-like *

Erect colonies having bilateral symmetry and conspicuous polyp leaves "like-branches" emerging from the rachis, resembling a quill pen. The axis is typically calcified conferring some rigidity to the overall structure

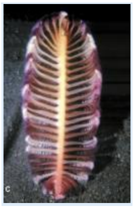

1.3.1. Feather-like: Erect colonies having bilateral symmetry and conspicuous polyp leaves "like-branches" emerging from the rachis, resembling a quill pen. The axis is typically calcified conferring some rigidity to the overall structure

(Photo by: K. Fabricius, in Fabricius and Alderslade, 2001; *Virgularia gustaviana)*

References

1. Bayer FM, Grasshoff M, and Verseveldt J (1983) Illustrated trilingual glossary of morphological and anatomical terms applied to Octocorallia. E.J. Brill.
2. Fabricius, K. and P. Alderslade (2001). Soft corals and sea fans: A comprehensive guide to the tropical shallow water genera of the central-west Pacific, the Indian Ocean and the Red Sea. Australian Institute of Marine Science, and the Museum and Art Gallery of the Northern Territory, 2001.
3. López González, P.J. & Zapata Guardiola, R. (2010). Four new species of *Thouarella* (anthozoa: octocorallia: primnoidae) from antarctic waters. *Scientia Marina*, 74 (1), 131-146.
4. Moore K, Alderslade P, Miller KJ (2017). A taxonomic revision of Anthothela (Octocorallia: Scleraxonia: Anthothelidae) and related genera, with the addition of new taxa, using morphological and molecular data. Zootaxa. 4304(1):1. 10.11646/zootaxa.4304.1.1

Table S6. Definition of each trait category for the Growth Form (basic) and Growth Form (detailed) traits, within the Erect Unbranched Type of Growth. An illustrative example of each detailed growth form has been included as well. References are shown under the table.

2.1 to 2.2 ERECT UNBRANCHED
Erect colonies that are devoid of branches and present a small area of attachment to the substrate in comparison with the height of the colony.

2.1. Erect unbranched
Erect colonies that are devoid of lateral parts. They can be slender or stout, but they are always higher than wide, occupying space mostly in the vertical plane.

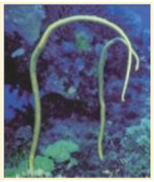

2.1.1. Flagelliform/filiform: Stout and/or thin, unbranched, whip-like (or thread-like) colonies that may reach important heights (e.g., up to 2 m in some cases).

(Photo by K. Fabricius; in Fabricius and Alderslade, 2001; *Junceella sp.)*

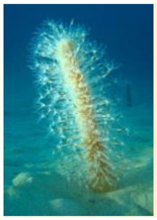

2.1.2. Erect unbranched (pen-like): Elongate and slender unbranched colonies with cylindrical form (pen-like). For simplicity reasons, we also include here those cylindrical to clavate or clavate forms that are elongate.

(Original photo by Runde Seefeder, CC-BY-SA-3.0; *Veretillum cynomorium*)

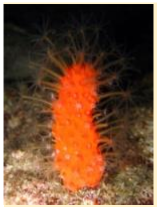

2.1.3. Digitiform: Erect unbranched, finger-like colonies that are cylindrical in shape. For simplicity reasons, we also include here those finger-like colonies with a thickened distal part (cylindrical to clavate or club-shaped)

(Original photo by Anders Poulsen, CC-BY-SA-3.0 Deed; *Paraminabea aldersladei*)

2.2 Erect unbranched (capitate)
Erect colonies that present a stem/stalk that is devoid of branches and a more or less wide, distinct capitulum bearing polyps at the distal part of the colony. The stem/stalk can be long or short, and the capitulum may be slightly or much wider than the stem/stalk, but in general, colonies in this group are still taller than wide with an area of attachment is relatively small.

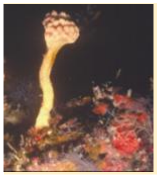

2.2.1 Erect unbranched with domed capitulum: Erect unbranched capitate colonies with a broad distal, dome-shape captiulum on a distinctly narrower stalk. Typical examples of this group are species of the genus *Anthomasthus, Nidalia*, or some *Xenias*

(Photo by Gary Williams, in Fabricius and Alderslade, 2001; *Nidalia sp.*)

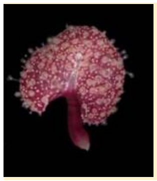

2.2.2 Erect unbranched with disc-shape capitulum: Erect unbranched capitate colonies with polyps restricted to the distal region of the colony, in a disc-shaped capitulum. The stalk is slender (e.g., *Renilla reniformis*).

(Image from DeVictor & Morton, 2010; *Renilla reniformis*)

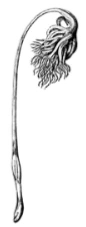

2.2.3 Erect unbranched with umbellate cluster of polyps: Elongate and slender unbranched capitate colonies with polyps restricted to the distal region of the colony, usually forming a dense umbellate cluster.

(Drawing from Kükenthal, 1920; *Umbellula anctartica*)

References

1. DeVictor, S.T.; Morton, S.L. (2010). Identification guide to the shallow water (0–200 m) octocorals of the South Atlantic Bight. Zootaxa. 2599: 1–62.
2. Fabricius, K. and P. Alderslade (2001). Soft corals and sea fans: A comprehensive guide to the tropical shallow water genera of the central-west Pacific, the Indian Ocean and the Red Sea. Australian Institute of Marine Science, and the Museum and Art Gallery of the Northern Territory, 2001.
3. Kükenthal, W. (1915). Pennatularia. Das Tierreich 43: 1–132.

Table S7. Definition of each trait category for the Growth Form (basic) and Growth Form (detailed) traits, within the Massive Type of Growth. An illustrative example of each detailed growth form has been included as well. References are shown under the table.

**3.1 MASSIVE**<br>Colonies voluminous, as high as wide and occupying a significant amount of space in all directions (hemispherical). The area of attachment to the substrate is also large.	
**3.1. Massive (hemispherical)**<br>Massive colonies growing in a domed, hemispherical way	
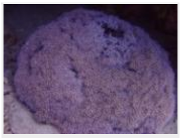	**3.1.1. Massive (hemispherical):** Massive colonies growing in a domed, hemispherical way. A typical example of this growth form in octocorals is *Tubipora musica*, with a domed growth form derived from vertical tubes joined by raised stolonic platforms.<br><br>(Photo by Tim Sheerman-Chase, CC BY 2.5; *Tubipora musica)*

Table S8. Definition of each trait category for the Growth Form (basic) and Growth Form (detailed) traits, within the Sub-Massive Type of Growth. An illustrative example of each detailed growth form has been included as well. References are shown under the table.

4.1 to 4.5. SUB-MASSIVE
Colonies also voluminous, somewhat as high as wide and occupying a significant amount of space in all directions, but not as robust as the Massive type. Instead, sub-massive colonies may have somewhat lumpy, columnar, lobate, plate-like or partially branched appearance. The area of attachment to the substrate is also relatively large.

4.1 Sub-massive (lobate/digitate)
Sub-massive colonies consisting of several slender (finger-like) or stout lobes extending both horizontally and vertically.

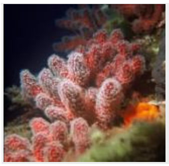

4.2.1. Sub-massive (lobate/digitate): Sub-massive colonies consisting of several slender (finger-like) or stout lobes extending both horizontally and vertically.

(Photo by Albert Kok, Public domain; *Alcyonium palmatum)*

4.2 Sub-massive (columnar)
Sub-massive colonies that emerge from a base in pillar forms and do not branch

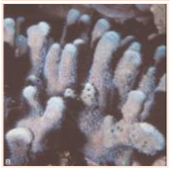

4.2.1. Columnar: Sub-massive colonies that emerge from a base in pillar forms and do not branch

(Photo by K. Fabricius, in Fabricius and Alderslade, 2001; *Heliopora coerulea)*

4.3 Sub-massive (branched)
Sub-massive colonies consisting of open branches emerging from an encrusting base

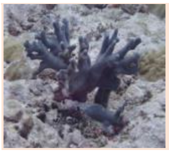

4.4.1. Sub-massive (branched): Sub-massive colonies consisting of open branches emerging from an encrusting base

(Photo by Ashton Williams, CC BY-NC-SA 4.0; *Heliopora coerulea)*

4.4 Sub-massive (foliose)
Sub-massive colonies that have a leaf-like or plate-like appearance

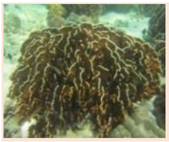

4.4.1. Sub-massive (foliose): Sub-massive colonies that have a leaf-like or plate-like appearance

(Photo by Floyd E. Hayes, CC BY-NC 4.0, *Heliopora coerulea)*

4.5 Sub-massive (with domed capitulum)
Sub-massive colonies that have a wide stalk and a domed polyparium leading to a somewhat hemispherical appearance.

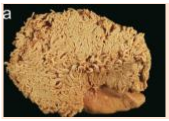

4.5.1. Sub-massive (with domed capitulum): Sub-massive colonies that have a wide stalk and a domed polyparium leading to a somewhat hemispherical appearance (e.g., *Yamazatum iubatum*; described as capitate xeniids with polyps arising from a firm dome shaped capitulum)

(Image from Benayahu, 2010; *Yamazatum iubatum)*

4.6 Sub-massive (plate-like)
Sub-massive colonies that have a large plate-like (or cup-like) polyparium on a distinctly narrower stalk, leading to colonies that are somewhat as wide as high in a typical mushroom shape.

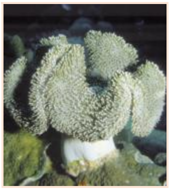

4.6.1. Plate-like: Sub-massive colonies that have a large plate-like (or cup-like) polyparium on a distinctly narrower stalk, leading to colonies that are somewhat as wide as high in a typical mushroom shape.

Photo by Roger Steene (in Fabricius and Alderslade, 2001); *Sarcophyton sp.)*

References

1. Benayahu, Y. (2010). A new genus of a soft coral of the family Xeniidae (Cnidaria: Octocorallia) from Japan. *Galaxea, Journal of Coral Reef Studies.* 12(2): 53-64., *available online at* https://www.jstage.jst.go.jp/article/galaxea/12/2/12_2_53/_article
2. Fabricius, K. and P. Alderslade (2001). Soft corals and sea fans: A comprehensive guide to the tropical shallow water genera of the central-west Pacific, the Indian Ocean and the Red Sea. Australian Institute of Marine Science, and the Museum and Art Gallery of the Northern Territory, 2001.

Table S9. Definition of each trait category for the Growth Form (basic) and Growth Form (detailed) traits, within the Horizontal Unbranched Type of Growth. An illustrative example of each detailed growth form has been included as well. References are shown under the table.

**5.1 to 5.2 HORIZONTAL UNBRANCHED**<br>Unbranched colonies with low profile that mostly extend horizontally, with very low vertical growth. They are also anchored to the substrate across a proportionally large area.	
**5.1 Membranous (sheet-like)**<br>Colonies consisting of a thin membrane covering the substrate (Bayer, 1983)	
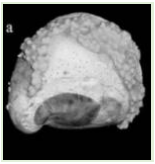	**5.1.1. Membranous (sheet-like):** Colonies consisting of a thin membrane covering the substrate (Bayer, 1983)<br>(Image from Van Ofwegene et al. 2006; *Incrustatus comauensis)*
**5.2 Encrusting**<br>Colonies consisting of a thick fleshy layer covering the substrate (Bayer, 1983). The surface may be flat or present relief.	
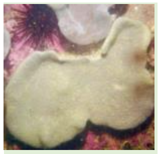	**5.2.1. Encrusting (flat):** mostly flat surface (sometimes plate-like or disc-shaped)<br>(Photo by K. Fabricius, in Fabricius and Alderslade, 2001; *Lobophytum sp.)*
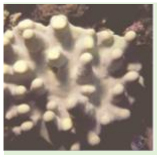	**5.2.2. Encrusting (digitate/lobate/folded):** Encrusting colonies with finger-like lobes, thick lobes or folded surfaces.<br>(Photo by K. Fabricius, in Fabricius and Alderslade, 2001; *Lobophytum sp.)*
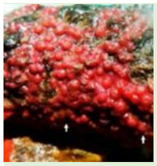	**5.2.3. Encrusting (with verrucae):** Encrusting colonies with verrucae<br>(Image from de la Cruz Francisco et al. 2019; *Callipodium rubens*)

<u>References</u>

1. Bayer FM, Grasshoff M, and Verseveldt J (1983) Illustrated trilingual glossary of morphological and anatomical terms applied to Octocorallia. E.J. Brill.
2. de la Cruz -Francisco V, Argüelles-Jiménez J, García Téllez ND, Rodríguez-Muñoz S, and León Méndez RG (2019). Presencia inusual de *Callipodium rubens* (Anthozoa: Cctocorallia) en raíces sumergidas de Rhizophora mangle en una laguna costera de Veracruz, México. Revista Colombiana de Ciencia Animal - RECIA, 11(2), 715. https://doi.org/10.24188/recia.v11.n2.2019.715
3. Fabricius, K. and P. Alderslade (2001). Soft corals and sea fans: A comprehensive guide to the tropical shallow water genera of the central-west Pacific, the Indian Ocean and the Red Sea. Australian Institute of Marine Science, and the Museum and Art Gallery of the Northern Territory, 2001.
4. van Ofwegen LP, Häussermann V, Försterra G (2006) A new genus of soft coral (Octocorallia: Alcyonacea: Clavulariidae) from Chile. Zootaxa 1219:47–57

Table S10. Definition of each trait category for the Growth Form (basic) and Growth Form (detailed) traits, within the Horizontal Branched Type of Growth. An illustrative example of each detailed growth form has been included as well. References are shown under the table.

6.1 to 6.2 HORIZONTAL BRANCHED
Branched colonies that mostly extend horizontally, with none or very low vertical growth.

6.1 Encrusting (stoloniferous)
Colonies having separate polyps budding off encrusting horizontal, branching stolons.

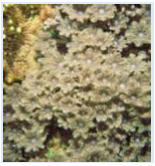

6.1.1. Encrusting (stoloniferous): Colonies having separate polyps budding off encrusting horizontal, branching stolons.

(Photo by K. Fabricius, in Fabricius and Alderslade 2001; *Clavularia sp.)*

6.2 Horizontal branched (with axial skeletal support)
Colonies forming axially supported branches that extend horizontally through the substrate, sometimes as creepers.

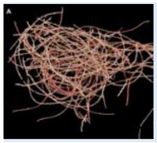

6.2.1. Horizontal branched (with axial skeletal support): Colonies forming axially supported branches that extend horizontally through the substrate, sometimes tangled.

(Image from Horvath, 2011; *Leptogorgia filicrispa*)

References

1. Horvath EA (2011). An unusual new "sea fan" from the northeastern Pacific Ocean (Cnidaria: Octocorallia: Gorgoniidae). Proceedings of the Biological Society of Washington, 124(1):45-52. https://doi.org/10.2988/10-27.1
2. Fabricius, K. and P. Alderslade (2001). Soft corals and sea fans: A comprehensive guide to the tropical shallow water genera of the central-west Pacific, the Indian Ocean and the Red Sea. Australian Institute of Marine Science, and the Museum and Art Gallery of the Northern Territory, 2001.

Table S11. Definition of each trait category for the Growth Form (basic) and Growth Form (detailed) traits, within the Solitary/Pseudo-solitary Type of Growth. An illustrative example of each detailed growth form has been included as well. References are shown under the table.

7.1. SOLITARY / PSEUDOSOLITARY
Octocorals growing as a single polyp or consisting of a single autozooid and numerous smaller siphonozooids

7.1 Solitary/Pseudosolitary
Octocorals growing as a single polyp or consisting of a single autozooid and numerous smaller siphonozooids

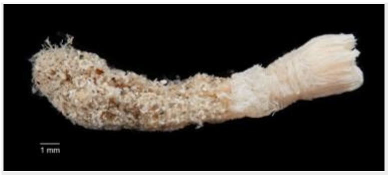

7.1.1. Solitary/Pseudosolitary: Octocorals growing as a single polyp or consisting on a single autozooid and numerous smaller siphonozooids

(Original photo by Claudia Ratti, CCby4.0Deed; *Taiaroa tauhou)*

Supplementary Figures

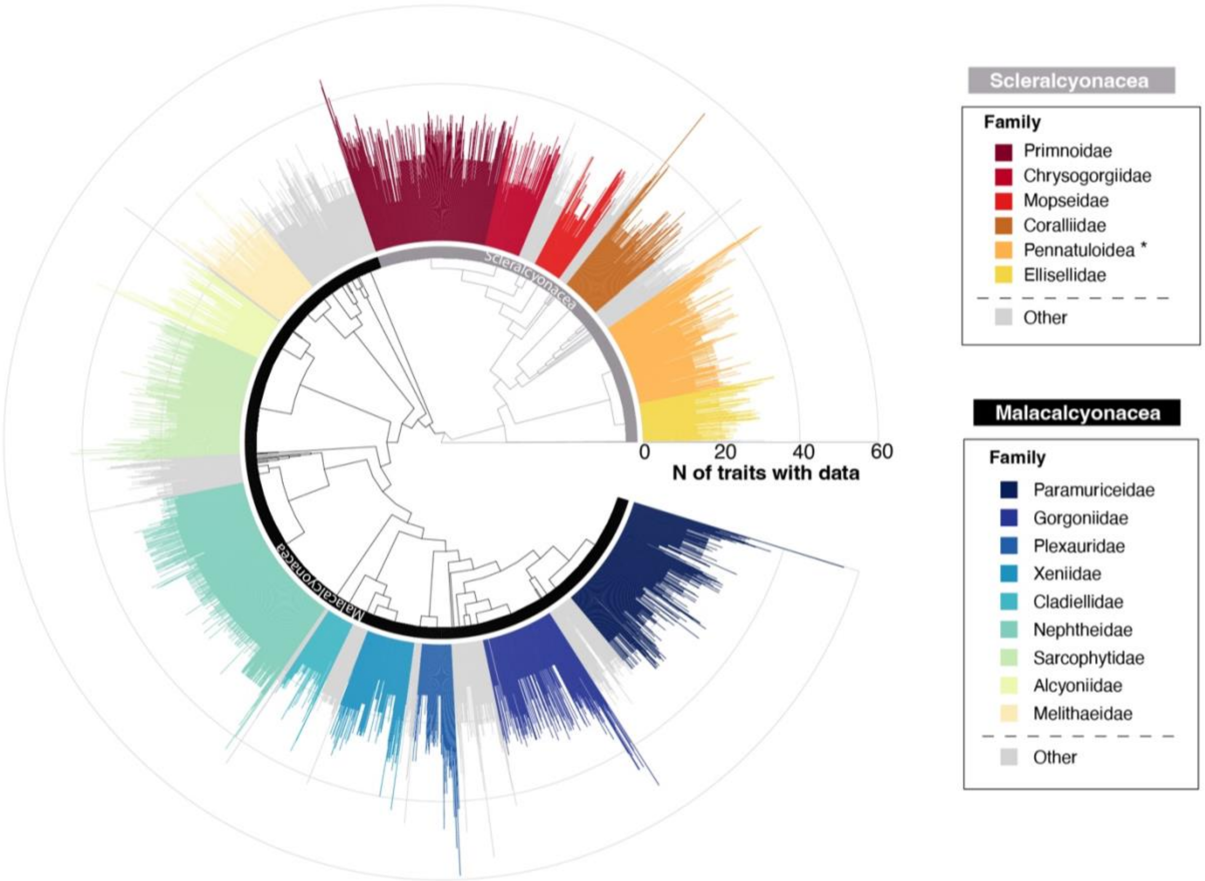

Figure S1. Species-complete tree with phylogenetic distribution of trait data coverage as number of different traits with data per species. The 15 largest families of the Class Octocorallia are labelled by colour. The two orders within the class, Scleralcyonacea and Malacalcyonacea, are also indicated. The phylogenetic tree added to this figure corresponds to an adaptation of the family-resolved Maximum likelihood tree of Octocorallia inferred from 1059 bp alignment of mitochondrial gene mtMutS (McFadden et al. 2022), with species being incorporated as polytomies. Data for species belonging to families that are currently *incertae sedis* have not been included in the figure.

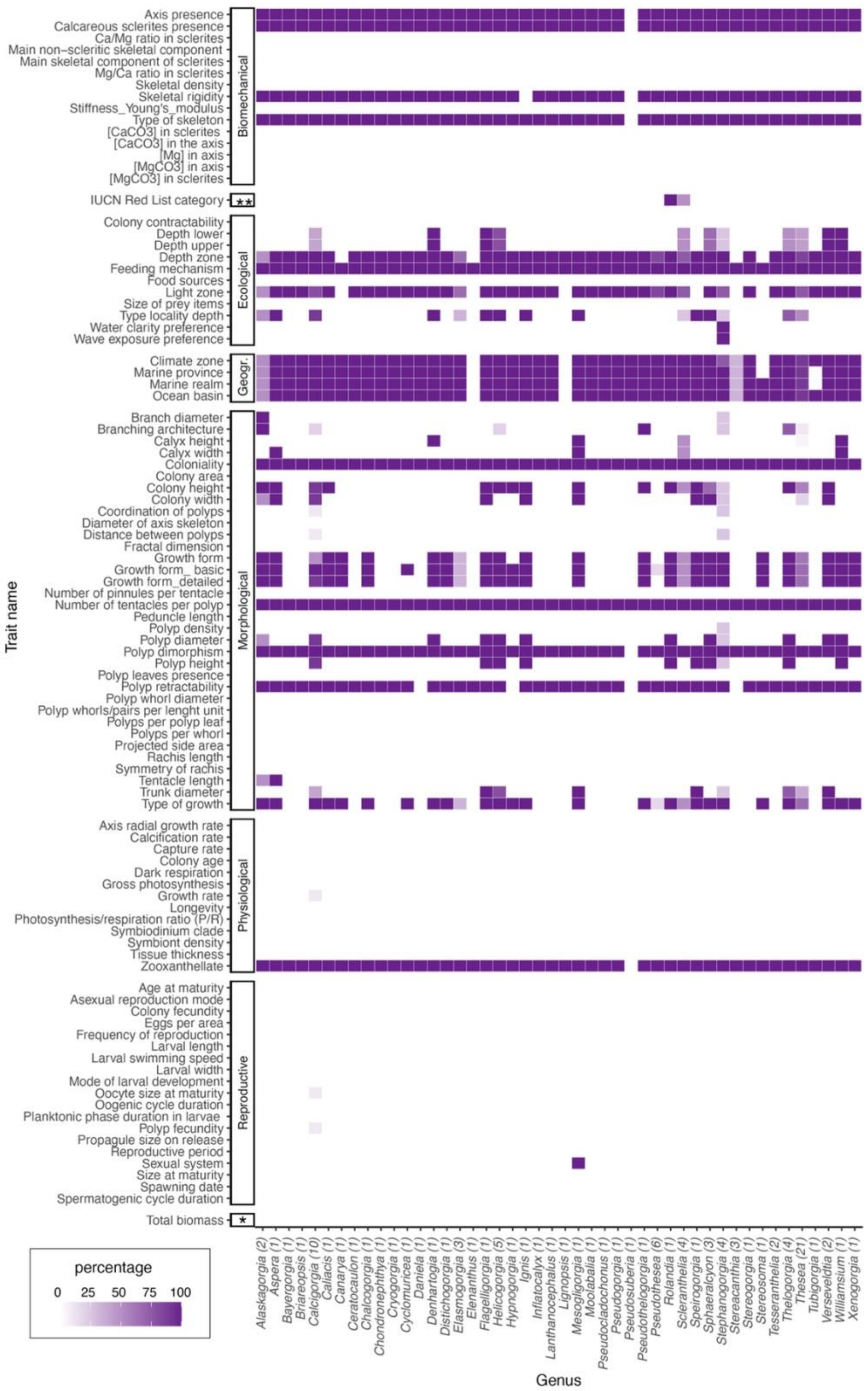

Figure S2. Distribution of trait data coverage across genera whose family assignation is *incertae sedis*. Parentheses indicate the number of species within a given genus. Purple color gradient indicates the % of species with data for a given trait, within a given genus. (**) refers to Conservation trait category, while (*) refers to Stoichiometric trait category. See **Table S1** for the trait definitions.
